# The somatic mutation profiles of 2,433 breast cancers refines their genomic and transcriptomic landscapes

**DOI:** 10.1038/ncomms11479

**Published:** 2016-05-10

**Authors:** Bernard Pereira, Suet-Feung Chin, Oscar M. Rueda, Hans-Kristian Moen Vollan, Elena Provenzano, Helen A. Bardwell, Michelle Pugh, Linda Jones, Roslin Russell, Stephen-John Sammut, Dana W. Y. Tsui, Bin Liu, Sarah-Jane Dawson, Jean Abraham, Helen Northen, John F. Peden, Abhik Mukherjee, Gulisa Turashvili, Andrew R. Green, Steve McKinney, Arusha Oloumi, Sohrab Shah, Nitzan Rosenfeld, Leigh Murphy, David R. Bentley, Ian O. Ellis, Arnie Purushotham, Sarah E. Pinder, Anne-Lise Børresen-Dale, Helena M. Earl, Paul D. Pharoah, Mark T. Ross, Samuel Aparicio, Carlos Caldas

**Affiliations:** 1 Cancer Research UK Cambridge Institute, Li Ka Shing Centre, University of Cambridge, Robinson Way, Cambridge CB2 0RE, UK; 2 Department of Oncology, University of Cambridge, Cambridge CB2 2QQ, UK; 3 Department of Cancer Genetics, Institute for Cancer Research, Oslo University Hospital Radiumhospitalet, Montebello, Oslo 0310, Norway; 4 The K.G. Jebsen Center for Breast Cancer Research, Institute for Clinical Medicine, Faculty of Medicine, University of Oslo, Oslo 0318, Norway; 5 Cambridge Breast Unit, Addenbrooke's Hospital, Cambridge University Hospital NHS Foundation Trust and NIHR Cambridge Biomedical Research Centre, Cambridge CB2 2QQ, UK; 6 Cambridge Experimental Cancer Medicine Centre, Cambridge University Hospitals NHS, Hills Road, Cambridge CB2 0QQ, UK; 7 Inivata, Li Ka Shing Centre, Robinson Way, Cambridge CB2 0RE, UK; 8 Peter MacCallum Cancer Centre, Melbourne, Victoria 3002, Australia; 9 Illumina, Chesterford Research Park, Little Chesterford, Essex CB10 1XL, UK; 10 Division of Cancer and Stem Cells, School of Medicine, University of Nottingham and Nottingham University Hospital NHS Trust, Nottingham NG5 1PB, UK; 11 Department of Pathology and Molecular Medicine, Queen's University/Kingston General Hospital, 76 Stuart Street, Kingston, Ontario, Canada K7L 2V7; 12 Department of Molecular Oncology, British Columbia Cancer Research Centre, Vancouver, British Columbia, Canada V5Z 1L3; 13 Research Institute in Oncology and Hematology, 675 McDermot Avenue, Winnipeg, Mannitoba, Canada R3E 0V9; 14 NIHR Comprehensive Biomedical Research Centre at Guy's and St Thomas' NHS Foundation Trust and Research Oncology, Cancer Division, King's College London, London SE1 9RT, UK; 15 Strangeways Research Laboratory, University of Cambridge, 2 Worts' Causeway, Cambridge CB1 8RN, UK

## Abstract

The genomic landscape of breast cancer is complex, and inter- and intra-tumour heterogeneity are important challenges in treating the disease. In this study, we sequence 173 genes in 2,433 primary breast tumours that have copy number aberration (CNA), gene expression and long-term clinical follow-up data. We identify 40 mutation-driver (Mut-driver) genes, and determine associations between mutations, driver CNA profiles, clinical-pathological parameters and survival. We assess the clonal states of Mut-driver mutations, and estimate levels of intra-tumour heterogeneity using mutant-allele fractions. Associations between *PIK3CA* mutations and reduced survival are identified in three subgroups of ER-positive cancer (defined by amplification of 17q23, 11q13–14 or 8q24). High levels of intra-tumour heterogeneity are in general associated with a worse outcome, but highly aggressive tumours with 11q13–14 amplification have low levels of intra-tumour heterogeneity. These results emphasize the importance of genome-based stratification of breast cancer, and have important implications for designing therapeutic strategies.

## Abstract (web summary)

Much effort has recently been devoted to understanding the genomics of breast cancer. In this study, the authors integrate somatic mutation data with previously published copy number aberration and gene expression information for nearly 2,500 breast cancer samples.

## Graphical abstract

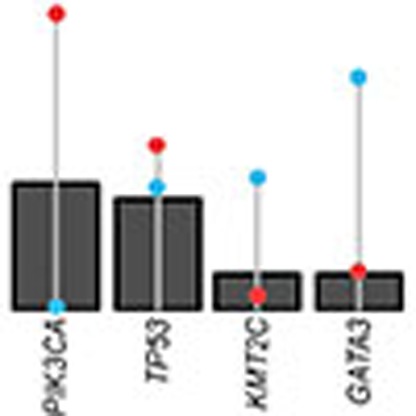

Human breast cancers are heterogeneous, and recent efforts have focused on characterizing both intra- and inter-tumour heterogeneity in a clinically relevant manner^1^. Current clinical management of the disease comprises morphological assessment (size, grade, lymph node status), and testing for oestrogen receptor (ER) and human epidermal growth factor receptor 2 (HER2). However, there is still much variation in the clinical outcomes of patients stratified based on these parameters. For instance, more than 75% of patients have ER+ breast cancers, but their outcomes and responses to therapy are extremely varied^2^. This clinical heterogeneity may be explained by the diversity of genomic drivers that underlie the disease.

To address this issue, we recently performed an integrated analysis of somatic copy number aberrations (CNAs) and gene expression profiles in 2,000 primary tumours (METABRIC)^3^,^4^. We observed that CNAs influencing expression in *cis* explained the greatest proportion of variance in gene expression, and were therefore likely to be enriched for driver genes. These driver CNAs were used in an unsupervised clustering approach to classify the tumours into 10 Integrative Clusters (IntClusts) that have distinctive copy number profiles and clinical courses. Readily identifiable driver genes include *CCND1* and *PAK1* at 11q13–14 in IntClust2, *ERBB2* at 17q12 in IntClust5, *ZNF703* at 8p11 in IntClust6 and *MYC* at 8q24 in IntClust9. IntClust10 encompasses a group of genomically unstable (mostly triple-negative) tumours that express basal markers. IntClust4 comprises tumours with relatively few CNAs (CNA-devoid). The Cancer Genome Atlas's (TCGA) Pan-Cancer analyses^5^, which demonstrated that CNAs dominate the genomic landscape of breast cancer, support the biological relevance of a copy number-based classification. This novel driver-based molecular taxonomy has also been robustly validated in 7,500 tumours^6^.

Although CNAs dominate the breast cancer genome, somatic SNVs and indels in driver genes are also important, and contribute to tumour biology. However, most breast cancer genes are somatically mutated at low frequencies and characterization of driver genes by high-throughput mutation profiling is consequently difficult when sample size is limited. This is exacerbated by stratification using clinical and molecular parameters. Large-scale efforts by the TCGA^7^ and the International Cancer Genome Consortium (ICGC)^8^ have contributed greatly towards enumerating breast cancer genes but analysis of clinical associations in these data sets is limited by the scarcity of long-term patient follow-up data and the stringent criteria used for sample selection (tumour size, malignant cellularity).

We have now sequenced 173 of the most frequently mutated breast cancer genes in 2,433 primary tumours to both identify driver genes and understand their clinical significance. The 173 genes sequenced include those that harboured mutations in at least 2 samples in 5 large-scale sequencing studies published in 2012 (refs 7, 8, 9, 10, 11) and also some targeted by homozygous deletions (HDs) we previously identified^3^. Crucially, long-term follow-up data were available for the majority of the cohort (2,319 patients, median=115 months). Our results outline the mutation profiles of key breast cancer genes and the associations between genomic and clinical features. The large number of samples with clinical annotation allowed characterization of mutation patterns and intra-tumour heterogeneity within specific subsets of tumours, and identification of associations with patient outcome within these subgroups.

## Results

### The mutation landscape of 173 genes in 2,433 breast tumours

We sequenced the exons of 173 genes (∼1.2 Mbp) in 2,433 primary breast tumours (Supplementary Table 1), reaching an average sequencing depth of at least 112 × in 80% of samples (median=152 × , Supplementary Fig. 1). Using a custom pipeline (Methods), we identified 32,476 somatic mutations, with 13,084 predicted to affect protein sequence. These coding mutations included 11,006 SNVs (10,193 missense, 808 nonsense, 5 read-through) and 1,635 small insertions or deletions (indels: 1,315 frameshift, 320 in-frame). We also detected 443 variants (268 SNVs, 175 indels) predicted to affect canonical splice sites. Each tumour had an average of 13 mutations (5 coding), with 131 tumours harbouring at least 30 mutations and 38 tumours devoid of any mutation (76 devoid of coding mutations). Details of mutations affecting all genes are provided in Supplementary Data 1.

As previously described^7^, *PIK3CA* (coding mutations in 40.1% of the samples) and *TP53* (35.4%) dominated the mutation landscape. Only five other genes harboured coding mutations in at least 10% of the samples: *MUC16* (16.8%); *AHNAK2* (16.2%); *SYNE1* (12.0%); *KMT2C* (also known as *MLL3;* 11.4%) and *GATA3* (11.1%) (Supplementary Fig. 2a,b). *MUC16*, *AHNAK2* and *SYNE1* are frequently mutated in several cancers (http://www.cbioportal.org^12^,^13^), but their roles in tumorigenesis are as yet uncertain. *In vitro* experiments have suggested a tumour-promoting role for *MUC16* in ovarian cancers^14^, but the gene also has a high background mutation rate^15^.

Predicted pathogenic germline mutations (Supplementary Fig. 2c) in *BRCA1* and *BRCA2* were identified in 1.36% and 1.64% of the cohort, respectively, and 2.22% of tumours harboured pathogenic *CHEK2* germline mutations. *TP53* pathogenic germline mutations were found in 0.82% of the tumours.

### A ratiometric method identifies 40 Mut-driver genes

**Figure 1:**
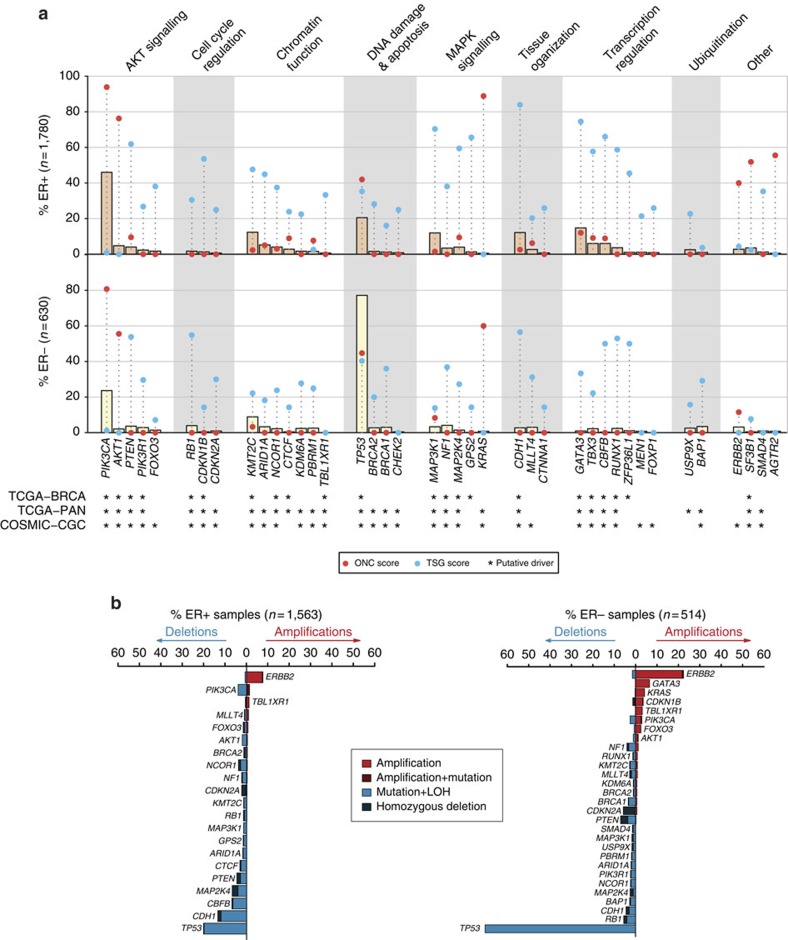
Identification of 40 mutation-driver genes in 2,433 primary breast cancer samples. (**a**) Bars depict proportions of ER+ and ER− samples harbouring mutations in mutation-driver (Mut-driver) genes. Red and blue points indicate for each gene, the proportions of recurrent (oncogene; ONC score) and inactivating (tumour suppressor gene; TSG score) mutations, respectively. ‘' indicates genes previously highlighted in other studies: COSMIC, Cancer gene census from the Catalogue of Somatic Mutations in Cancer; TCGA-BRCA, TCGA breast cancer study; TCGA-PAN, TCGA pan-cancer analysis. ER status available for 2,410 tumours. MAPK, mitogen-activated protein kinase. The genes are grouped by pathway or function. (**b**) Bars depict proportion of tumours with copy number alterations (CNAs) in genes altered in at least 1% of ER+ or ER− samples. The percentages of tumours with amplifications, simultaneous amplification and mutation events, homozygous deletions and simultaneous mutations and LOH events are shown. LOH was defined as any CNA in which with either the major or minor allele was entirely deleted as determined by ASCAT (Methods).

Genes mutated in cancer comprise drivers, whose deregulation contributes directly to tumour progression, and passengers, which typically provide little or no selective advantage when mutated. To identify candidate driver genes, we used the scheme proposed by Vogelstein *et al*.^16^. This method is derived from the observed mutation patterns of well-characterized driver genes, and identifies candidate oncogenes and tumour suppressors based on the proportion of recurrent mutations (nonsynonymous SNVs, in-frame indels, oncogene score (ONC)) or inactivating mutations (frameshift indels, nonsense SNVs, splice site mutations, tumour suppressor gene score (TSG)) they harbour. In addition, we required that the candidates had a minimum of five recurrent or inactivating mutations. Exploration of a range of relevant thresholds demonstrated that the recommended cut-off of 20% was robust (Supplementary Fig. 3a). As originally proposed, genes with an ONC score of at least 20% that also had a TSG score of at least 5% were classified as tumour suppressors, as oncogenes rarely harbour inactivating mutations. After stratifying by ER status, we identified 40 genes (22 in ER+ only, 3 in ER− only, 15 shared) that are here on referred to as Mut-drivers genes (Fig. 1a, Supplementary Data 2). The spectra of mutations for the 40 genes are depicted in Supplementary Fig. 4. Only 6 out of 40 Mut-driver genes identified were oncogenes.

The list of Mut-driver genes includes well-established breast cancer oncogenes and tumour suppressors such as *PIK3CA* and *TP53*. *TP53*, categorized as a tumour suppressor, had high ONC and TSG scores in both ER+ (ONC=42%, TSG=35%) and ER− (ONC=45%, TSG=40%) tumours. The list also provides further evidence for the importance of other breast cancer genes that have more recently been reported. For example, *TBX3* had high TSG scores in ER+ (TSG=58%, 6.0% of ER+ samples with coding mutations) and ER− (22%, 2.2%) tumours, and was also significantly mutated relative to the background mutation rate in the TCGA data set^7^. Inactivating mutations in the binding partners *CBFB* (ER+: TSG=66%, 6.0%; ER−: 50%, 0.32%) and *RUNX1* (also known as *AML1*; ER+: 59%, 3.7%; ER−: 53%, 2.4%) were also common. In addition, we observed recurrent K700E mutations in *SF3B1* (ER+: ONC=52%, 3.5%) which have recently been associated with differential splicing activity in breast tumours^17^. *AGTR2* harboured 6 P271L mutations (ER+: ONC=56%, 0.51%), and was also found to be mutated at a significant frequency in a recent study of 77 breast tumours^11^. The presence of these genes in the Mut-driver list provides further support for their roles in breast cancer.

We identified other Mut-driver genes that have not been formally associated with breast cancer^7^, but have been reported in other cancer types or in pan-cancer analyses. There were high proportions of inactivating mutations in the SWI/SNF complex members *ARID1A* (ER+: TSG=45%, 5.2%; ER−: 18%, 3.3%) and *PBRM1* (ER+: TSG=3%, 1.6%; ER−: 25%, 2.5%). Overall, 22.6% of tumours harboured a coding mutation in one of the seven Mut-driver genes involved in chromatin function (*KMT2C*, *ARID1A*, *NCOR1*, *CTCF*, *KDM6A*, *PRBM1* and *TBL1XR1*). We also identified canonical activating mutations in members of the Ras pathway. *KRAS* codon 12 mutations were present in 11 samples, and the gene had high ONC scores in both ER+ and ER− cancers (ER+: 89%, 0.5%, ER−: 60%, 0.8%). We also observed mutations in *HRAS* at codons 61 and 12 (two and one samples, respectively), and a single *BRAF* V600E mutation in an ER− tumour, although the two genes did not meet Mut-driver criteria. These classical activating mutations are characteristic in other cancer types^18^,^19^ and are actionable mutations in those settings^20^.

The mutation patterns of some Mut-driver genes differed by ER status. For example, *SMAD4* was mutated in 1.2% of ER+ and 1.0% ER− tumours, but had a high TSG score only in ER+ cases (ER+=35%, ER−=0%; Supplementary Fig. 4). *SMAD4* expression has previously been linked with apoptosis in an ER+ context^21^. Similarly, *ERBB2* mutations were found in similar proportions in ER+ (2.8%) and ER− tumours (3.2%), but there was a higher proportion of mutations at codon 755 in ER+ tumours (13 out of 53 ER+, 1 out of 22 ER−; *P*=0.05331, Fisher's exact test) and the gene had a higher ONC score than in the ER− cohort (ER+: ONC=40%, ER−: ONC=12%). On the other hand, although *PIK3CA* had a high ONC score in both ER+ (94%) and ER− (81%) tumours, the domains in which the recurrent mutations occurred differed (Supplementary Fig. 4). ER+ tumours had more *PIK3CA* mutations in codons 345 (62 out of 942 ER+, 2 out of 162 ER−; *P*=0.003396, Fisher's exact test) and codons 542 or 545 (helical domain; 259 out of 942 ER+, 32 out of 162 ER−; *P*=0.04245). In contrast, *PIK3CA* mutations in codon 1047 (kinase domain) were more common in ER− tumours (83 out of 162 ER− versus 382 out of 942 ER+ *P*=0.01243,). Mutations in the helical and kinase domains have been shown to have different functions and biochemical interactions^22^,^23^.

We also analyzed CNAs affecting the Mut-driver genes, as these represent alternative mechanisms of somatic gene deregulation. Figure 1b shows the proportions of tumours with amplifications (defined here as 5+ gene copies) and HDs or loss of heterozygosity (LOH) of the Mut-driver genes affected by CNAs in at least 1% of either ER+ or ER− tumours. Amplification of the known oncogenes *KRAS*, *PIK3CA* and *AKT1* was more common in ER− tumours (3.9%, 2.7%, 1.2% respectively). We identified LOH events in 96.0% tumours that harboured *CDH1* mutations, and in 85.4% of *TP53*-mutant tumours (Supplementary Fig. 5).

### HDs identify candidate tumour suppressor genes

We previously reported several genes targeted by HDs^3^, and sequenced 40 of these to identify inactivating mutations (nonsense SNVs, frameshift indels, splice site mutations) and provide further evidence for their roles in breast cancer^24^ (Supplementary Fig. 6, Supplementary Data 1). Of the 40 genes, 8 were independently identified as Mut-driver tumour suppressor genes using the ratiometric method described above: *FOXO3*, *CTNNA1*, *FOXP1*, *MEN1*, *CHEK2* in ER+ tumours; *CDKN2A*, *KDM6A* and *MLLT4* in both ER+ and ER− tumours. These genes were not mutated at significant rates in the TCGA data set, possibly due to smaller sample size. *CDKN2A* (also known as *P16-INK4A)* was the most common target of HDs (53 out of 2,087 tumours with copy number data). Although *CDKN2A* mutations are relatively uncommon in breast cancer^25^, 6 of our tumours had inactivating mutations. *FOXO3* had 9 HDs and 14 inactivating mutations, and *FOXP1* had 3 HDs and 7 inactivating mutations. Low expression of these two genes has been previously linked with poor outcome in breast cancer^26^,^27^.

We noted that *CDH1*, *CTNNA1* and *MLLT4* encode proteins that are involved in cell adhesion at adherens junctions. *CDH1*, *MLLT4* and *CTNNA1* were deleted in 29, 7 and 1 tumours, respectively, and harboured inactivating mutations in 169, 20 and 4 tumours, respectively. E-cadherin, encoded by *CDH1*, is linked to the actin cytoskeleton by alpha-catenin (encoded by *CTNNA1*). Previous analyses have suggested that *CTNNA1* is a tumour suppressor gene^24^, and it had a TSG score of 26% in ER+ tumours. *MLLT4* (TSG scores: ER+=20%, ER−=31%) encodes afadin, which forms a bridge between nectin and F-actin^28^,^29^. These mutation data strongly indicate that these functionally related genes are breast cancer tumour suppressors.

Of the remaining 32 genes in the HD list, 30 harboured at least 1 inactivating mutation. Two of these genes were associated with immune function. *JAK1*, with 4 HDs, 4 inactivating mutations and 4 missense SNV/LOH events, has previously been linked with immune evasion in gynaecological tumours^30^. *NT5E*, which encodes CD73, has been linked with immune modulation and anthracycline response^31^, and had 3 HDs, 1 inactivating mutation and 4 missense SNV/LOH events in our data set.

### Associations with clinical and pathological parameters

**Figure 2:**
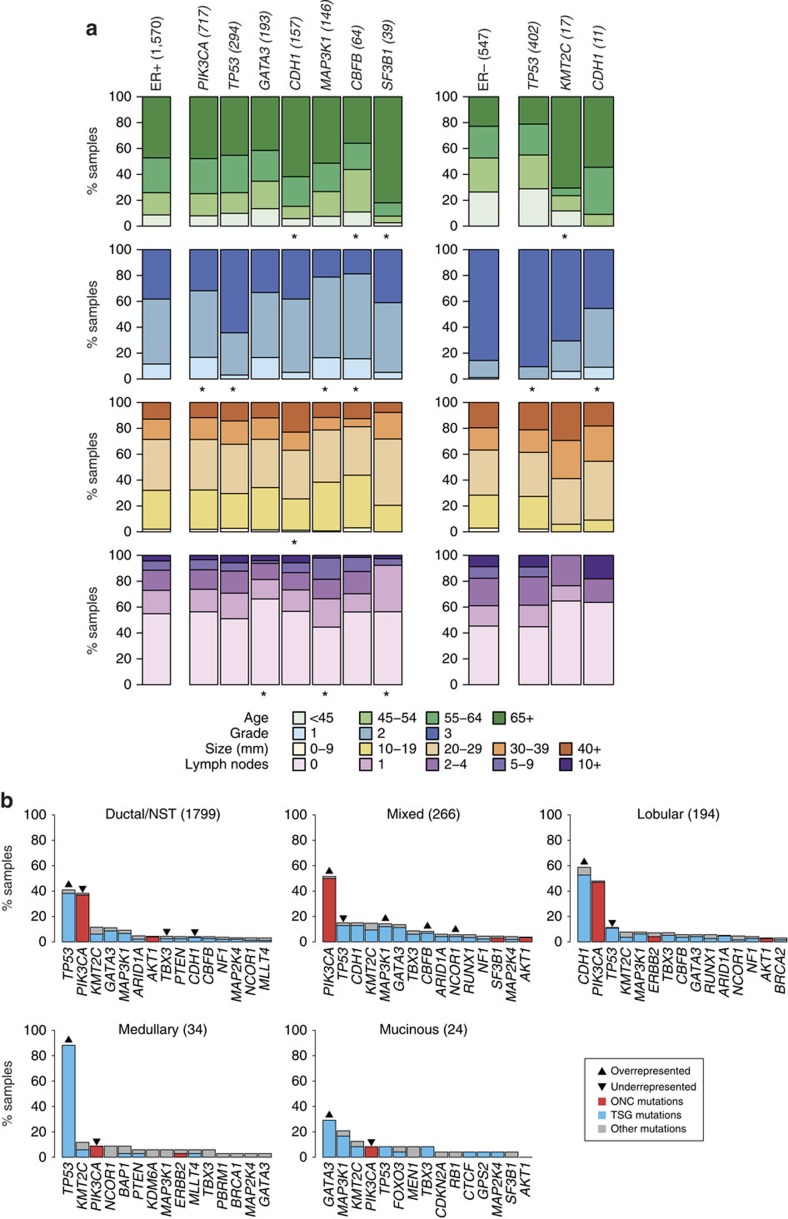
Associations between mutations and clinical-pathological variables. (**a**) The associations between functional mutations in Mut-driver genes and patient age, tumour grade, size and number of lymph nodes involved are depicted for ER+ (left) and ER− (right) samples. Bars depict the categorical distributions of each variable in samples harbouring a functional mutation in the specified gene. The single bars on the left of each panel show the distributions of the variables for either all ER+ or ER− samples. The numbers of samples with mutations in the genes are shown in brackets. For each gene, we looked for a difference in the distributions of a variable between wild-type and mutant samples. All genes for which at least one association was found (*χ*^2^-test; FDR=0.05) are shown, and ‘' indicates the significant associations. The analysis was performed for genes mutated in at least 1% of ER+ or ER− samples. (**b**) Bars depict prevalence of mutations in Mut-driver genes across histological subtypes. The 15 most frequently mutated genes in each subtype are shown. The coloured part of each bar indicates functional mutations, which were defined as recurrent mutations that contribute to an oncogene's ONC score (red), or inactivating mutations that contribute to a tumour suppressor gene's TSG score (see main text). Both recurrent and inactivating mutations were considered for *TP53*. Up arrows and down arrows indicate over/under-representation of mutations, respectively, in the specified gene relative to all other samples (Fisher's exact test; FDR=0.05). NST, no special type.

We used a multivariable logistic regression model (Methods) to identify associations between mutations in Mut-driver genes and clinical-pathological parameters (Fig. 2a). We focused only on functional mutations, which were defined as those that contributed towards a gene's ONC (recurrent mutations) or TSG (inactivating mutations) score for oncogenes or tumour suppressors, respectively (Supplementary Table 2). For *TP53*, we used both recurrent and inactivating mutations.

Functional mutations in *PIK3CA* (odds ratio (OR)=0.58; 95% confidence interval (CI)=0.49–0.69), *GATA3* (OR=0.77, CI=0.6–0.99), *MAP3K1* (OR=0.52, CI=0.4–0.68)*, KMT2C* (OR=0.69, CI=0.52–0.94) and *CBFB* (OR=0.56, CI=0.38–0.83) were associated with lower grade in ER+ tumours. Inactivating mutations in *GATA3* (OR=0.63, CI=0.45–0.89) and *CBFB* (OR=0.48, CI=0.28–0.81) were more common in patients diagnosed at younger ages, whereas patients presenting with mutations in *CDH1* (OR=1.9, CI=1.2–3), *KMT2C* (OR=2.1, CI=1.3–3.6) and *SF3B1* (OR=4.5, CI=1.6–19) tended to be older. Mutations in *TP53* were associated with higher grade in both ER+ (OR=3.3, CI=2.6–4.2, *P*<0.001) and ER− (OR=3.6, CI=2.1–6.2, *P*<0.001) tumours. Mutations in *CDH1* (OR=0.12, CI=0.034–0.38) and *ERBB2* (OR=0.16, CI=0.042–0.59) were uncommon in ER− cancers, but were associated with lower grade.

HER2 status is a key parameter in the clinical management of breast cancer. The TCGA previously noted that the mutation profiles of HER2+ tumours varied by ER status^7^. Similarly, we observed more functional mutations in *TP53* in HER2+/ER− tumours (67.5%) than in HER2+/ER+ tumours (42.6%), and more functional mutations in *GATA3* in the HER2+/ER+ cohort (8.2%; HER2+/ER−=0.5%) (Supplementary Fig. 7). However, the prevalence of functional *PIK3CA* mutations was similar between the two groups (HER2+/ER+=29.5%, HER2+/ER−=30.1%), although there were significantly fewer *PIK3CA* mutations in HER2+/ER+ tumours than in HER2−/ER+ tumours (46.5%). *PIK3CA* mutations may have independent driver properties in a HER2+ context^32^ and have been implicated in resistance to anti-HER2 therapies^33^.

The relative frequencies of functional mutations in tumours classified into five histological types are presented in Fig. 2b. Mucinous (8.3%) and medullary (8.8%) carcinomas had significantly fewer functional *PIK3CA* mutations relative to other subtypes (lobular=46.9%, mixed=50.0%, ductal=36.9%). Inactivating mutations in *CDH1* were observed in 52.6% of lobular carcinomas, and *CDH1* was also the most frequently mutated gene in lobular carcinomas in the TCGA data set^34^. Loss of E-cadherin function is a hallmark of invasive lobular carcinoma^35^. Only 3.4% of ductal/NST carcinomas had inactivating *CDH1* mutations. HD of *CDH1* was observed in 18 ductal/NST carcinomas (1.1%) and in 4 lobular carcinomas (2.1%). These results suggest that *CDH1* may act as a tumour suppressor in a small fraction of ductal/NST cancers.

### Associations between somatic alterations

**Figure 3:**
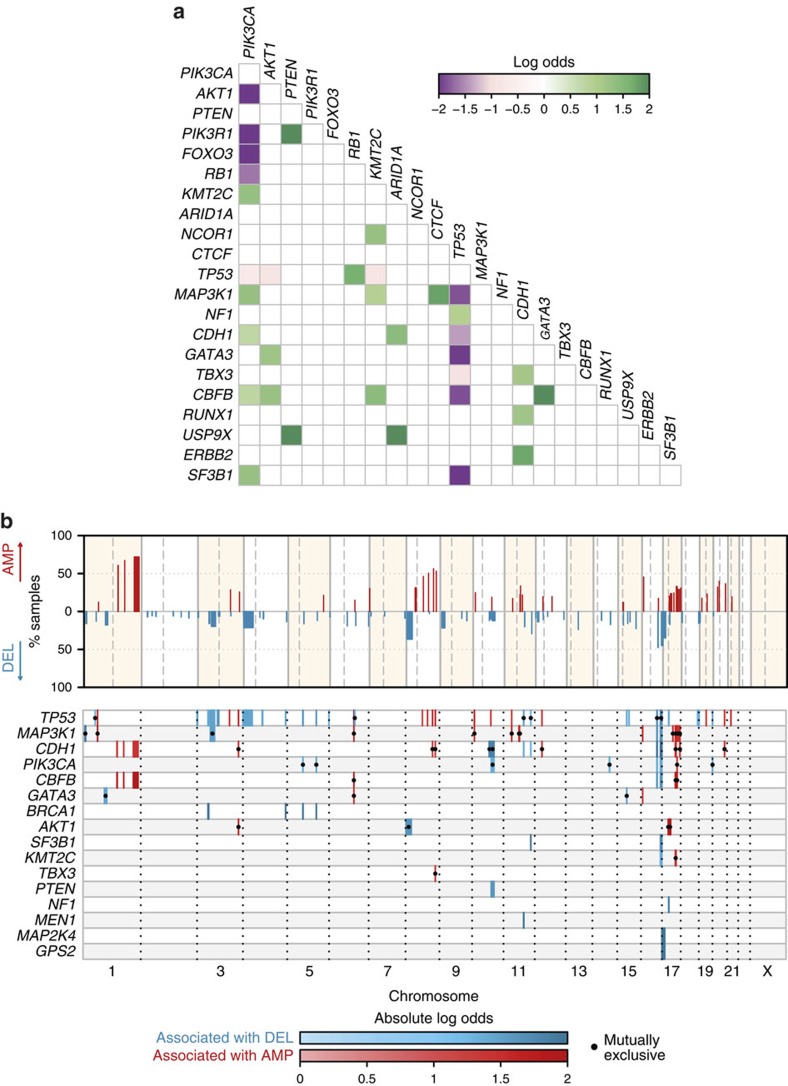
Patterns of association between somatic events. (**a**) Pairwise association plot for 40 Mut-driver genes in 2,433 samples. Purple squares represent negative associations (mutually exclusive mutations); green squares represent positively associated events (co-mutation). The colour scale represents the magnitude of the association (log odds). We considered all genes mutated in at least 0.5% of the entire cohort, and only associations at FDR=0.1 are shown (Fisher's exact test). (**b**) Association plot of CNAs and Mut-driver gene mutations. Top panel: significantly recurrent copy number aberrations (CNAs) identified by GISTIC2 are shown across the genome, along with the percentage of samples affected by the particular CNA. Bottom panel: plot showing Mut-driver gene mutations associated with CNAs. Associations (Ass.) with amplifications and deletions are coloured red and blue respectively, and the colour scale corresponds to the magnitude of the association (log odds). Associations with dots represent mutual exclusivity and those without dots represent co-occurrence. Only genes with at least one significant association (Fisher's exact test; FDR=0.01) are shown, and only associations with absolute log odds ⩾log(2) were considered.

To explore patterns of co-mutation and mutual exclusivity, we examined pairwise associations between somatic events using Fisher's exact test. Significant pairwise interactions (false discovery rate (FDR)=0.1) between functional mutations in Mut-driver genes are depicted in Fig. 3a. Mutual exclusivity between mutations in *PIK3CA* and *AKT1* (OR=0.017, CI=0.00044–0.1), between *PIK3CA* and *PIK3R1* (OR=0.092, CI=0.0022–0.59), and between *PIK3CA* and *FOXO3* (OR=0.1, CI=0.0025–0.68) reflect functional redundancy within the Akt signalling pathway. Surprisingly, 15 out of 57 tumours harbouring *PTEN* inactivating mutations also had recurrent *PIK3CA* mutations. Three out of 18 tumours with *PIK3R1* functional mutations also had *PTEN* mutations. Overall, 45.2% of all tumours had a functional mutation in at least one member of the Akt signalling pathway (*PIK3CA*, *AKT1*, *PIK3R1*, *PTEN* and *FOXO3*). Mutual exclusivity between mutations in *TP53* and each of *CDH1* (OR=0.23, CI=0.14–0.36), *GATA3* (OR=0.13, CI=0.069–0.23) and *SF3B1* (OR=0.049, CI=0.0012–0.29) reflect opposite associations with tumour grade and histological type (Fig. 2). Co-mutation of *TP53* and *RB1* (OR=5.3, CI=2.2–14) is common in triple-negative cancers^10^. We observed co-mutation of *CDH1* and *PIK3CA* (OR=2.1, CI=1.6–2.9), *CDH1* and *TBX3* (OR=3.2, CI=1.7–5.7), and *CDH1* and *RUNX1* (OR=3.3, CI=1.5–6.6) as has previously been noted in lobular carcinomas^34^. We observed co-mutation of *ERBB2* and *CDH1* (OR=5.7, CI=2.7–12). Mutations in *ERBB2* have been observed in relapsed *CDH1*-mutant lobular carcinomas^36^. Co-mutation of *CBFB* and *GATA3* (OR=7.7, CI=4.6–13) may reflect their role in ER-mediated gene activation^37^.

We also explored associations between mutations and recurrent CNAs identified by GISTIC (Fig. 3b). Most tumour suppressor genes displayed classic mutation/LOH associations, including *PTEN* and 10q23.1 deletion (OR=3.4; 95% CI=1.7–6.6), and *GPS2* and 17p13.1 deletion (OR=7.1; CI=2.3–29). Positive associations were also observed between genes more frequently mutated in ER+ breast cancer and concurrent 1q gain/16q loss (probably due to t(1q;16p) translocation). These associations are particularly strong for mutations in *CDH1* (OR=2, CI=1.4–3.1) and *CBFB* (OR=5, CI=2.3–12), both of which reside at the 16q22 locus^38^. Mutual exclusivity was observed between *AKT1* mutations and amplification of *ERBB2* at the 17q12 locus (OR=0.091; CI=0.011–0.34). *ERBB2* signalling is known to activate the PI3K/Akt signalling pathway^39^.

### Landscape of Mut-driver mutations across the IntClusts

**Figure 4:**
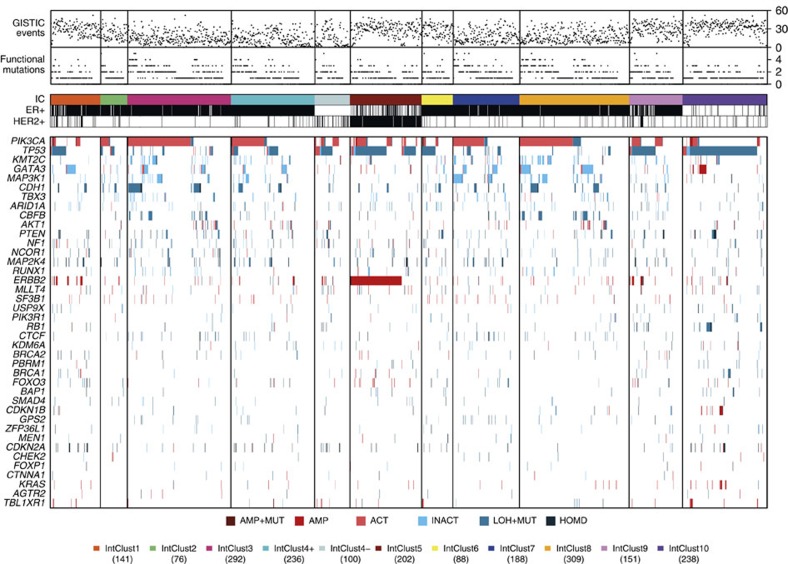
Genomic profiles of the Integrative Clusters. Tumours with both mutation and copy number data available (*n*=2,021) are grouped by IntClust along the *x*-axis, and alterations in the 40 Mut-driver genes are indicated by coloured bars. For each tumour, the number of functional mutations in Mut-driver genes and the number of recurrent CNAs (as defined by GISTIC2) events are also shown. AMP, amplification; ACT, activating mutation; HOMD, homozygous deletion; INACT, inactivating mutation; LOH+MUT, mutation and hemizygous deletion.

The mutation and copy number landscapes of the 40 Mut-driver genes across the IntClusts are depicted in Fig. 4. The number of mutations in Mut-driver genes in individual tumours was similar across IntClusts, but the relative frequencies of gene mutations varied significantly (Supplementary Table 3, Supplementary Figs 8 and 9). For example, *TP53* has functional mutations in 84.6% of IntClust10, 64.2% of IntClust5, 50.5% of IntClust4-, and 44.7% and 40.7% of IntClusts9 and 6, respectively, both of which largely comprise ER+ tumours. In contrast, *TP53* mutations occurred in only 10.0% of IntClust3, 14.0% of IntClust7 and 4.4% of IntClust8, which are subtypes composed almost exclusively of ER+ good prognosis tumours. IntClust1 (29.0%), IntClust2 (24.1%) and IntClust4+ (21.1%) had intermediate frequencies of functional *TP53* mutations.

**Figure 5:**
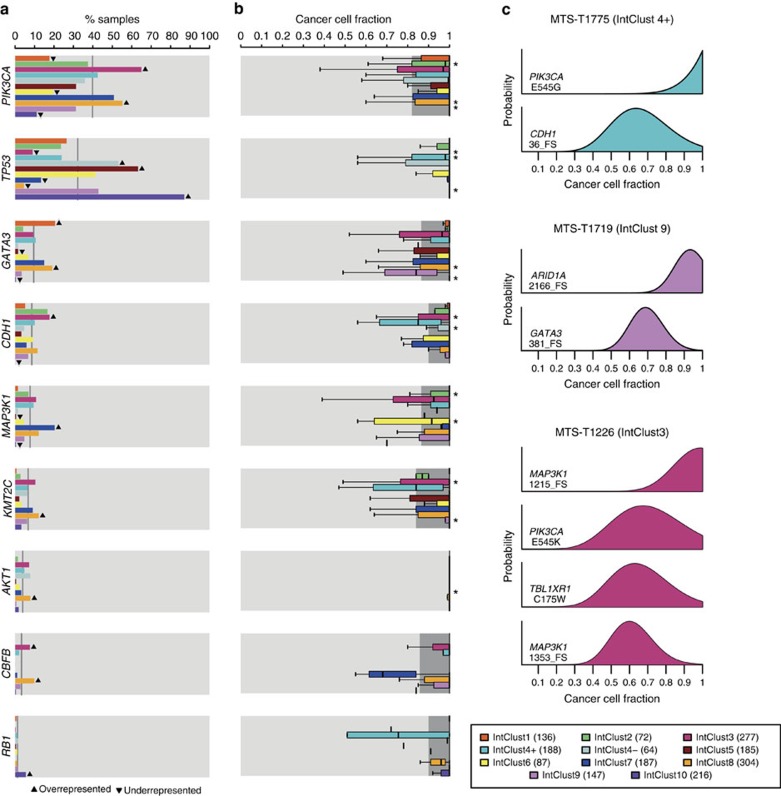
Prevalence and clonal states of Mut-driver mutations across the Integrative Clusters. (**a**) Bars showing prevalence of mutations for the nine Mut-driver genes that were either under- or over-represented in one of the IntClusts relative to all other samples (Fisher's exact test; FDR=0.05). Up arrows and down arrows indicate over/under-representation of mutations, respectively, in the specified IntClust. The grey lines represent mutation prevalence of the indicated gene for all samples in the cohort. (**b**) Box plots depicting cancer cell fractions (CCFs) of mutations in the nine genes across the IntClusts. CCFs were estimated as described in Methods, and we compared the CCF distribution of a gene's mutations in each IntClust with that of all other tumours. The dark grey shading represents interquartile ranges and outliers are not shown for the purpose of clarity. ‘*' indicates a significantly different CCF distribution (two-sample Wilcoxon test, *P*=0.05). (**c**) Example plots of CCF distributions in individual samples. Three samples (MTS-T1775, MTS-T1719 and MTS-T1226) were considered, and the IntClust to which they belong are also indicated. FS, frameshift indel.

To formally identify interactions between IntClust and driver mutations, we looked for Mut-driver genes that were mutated in significantly higher or lower proportions of tumours in at least one IntClust relative to the remaining samples. This analysis identified nine genes at FDR=0.01 (Fig. 5a, Supplementary Fig. 8). Mutations in *CBFB* (3.3% across all tumours) were more frequent in IntClust3 (7.8%) and IntClust8 (9.7%), and less common in IntClust7 (1.0%), although patients within all these subtypes have relatively good outcomes (Supplementary Fig. 10a). *GATA3* mutations (8.9% across all tumours) were more frequent in IntClust1 (20.0%) and IntClust8 (19.5%), although patients in IntClust1 have worse outcomes.

One of the key features of the IntClust classification is stratification of ER+ tumours (Supplementary Fig. 10b, Supplementary Table 4). We therefore explored the distributions of mutations in Mut-driver genes in the ER+ subsets of the IntClusts (Supplementary Fig. 11). There was a high prevalence of functional *TP53* mutations in ER+ tumours belonging to IntClust10 (59.3 versus 18.7% in all ER+ tumours). The CNA profiles of the 27 IntClust10+ are more similar to triple-negative basal-like cancers than to ER+ breast cancers.

Finally, we grouped the 40 Mut-driver genes into pathways (Supplementary Fig. 12), and scored the tumours as pathway-altered if at least 1 of the genes in the pathway was affected by mutation or CNA. IntClusts 3 and 7 comprise tumours with similar clinical characteristics (Supplementary Fig. 10), but IntClust7 had a higher frequency of alterations in the MAP kinase (MAPK) signalling pathway (IntClust3=20.5%, IntClust7=24.6%), and there were more alterations in tissue organization genes in IntClust3 (IntClust3=24.0%, IntClust7=10.6%). IntClust1 had fewer alterations in the Akt signalling pathway (30.5%), despite comprising mostly ER+ tumours. Alterations in DNA damage response genes were present in 89.9% of IntClust10 (mostly triple-negative, basal-like) tumours, and were also frequent in IntClust9 tumours (mostly ER+ and poor prognosis; 52.3%). Tumours in IntClust10 also had more alterations in cell cycle regulation (20.2%) and ubiquitination (6.7%) genes, but had fewer alterations in the Akt signalling pathway (27.7%).

### Clonal states of Mut-driver genes

To characterize the clonal states of Mut-driver gene mutations across the IntClusts, we used variant allele fractions (VAF) and copy number data to obtain estimates of the fraction of cancer cells harbouring mutations^40^ (Fig. 5b, Supplementary Figs 13–15). The distributions of the cancer cell fractions (CCFs) of functional mutations in the Mut-driver genes were centred largely around 1, indicating their presence in nearly all tumour cells and likely early occurrence in tumour evolution (Supplementary Fig. 13). However, differences between IntClusts were apparent. In general, tumours in IntClusts constituted by patients with better prognosis (IntClusts 3, 7, 8) had lower proportions of clonal mutations in driver genes relative to IntClusts with patients with worse outcomes (IntClusts 2, 10) (Supplementary Fig. 14). Nearly all activating *PIK3CA* mutations in IntClusts10 (median CCF=1, interquartile range (IQR)=1–1, 26 mutations) and IntClust9 (median=1, IQR=0.98–1, 49 mutations) were present at CCFs close to 1, whereas some *PIK3CA* mutations in IntClust3 tumours were present in lower CCFs (median=0.96, IQR=0.75–1, 215 mutations). IntClusts 2 and 6 are relatively small subgroups comprising ER+ cancers with poor outcome, but *MAP3K1* mutations in IntClust2 (median=1, IQR=0.91–1, seven mutations) were present in higher CCFs than in IntClust6 (median=0.90, IQR=0.69–0.99, six mutations).

Characteristic examples are displayed in Fig. 5c, which shows the probability distributions of mutation CCFs in individual tumours. MTS-T1775 (IntClust4+) has a clonal *PIK3CA* mutation (CCF=1, CI=1–1) and a *CDH1* inactivating mutation present in a lower CCF (0.64; CI=0.39–1). This pattern appears to be characteristic of IntClust4+ tumours, which frequently appear to harbour more subclonal *CDH1* mutations than those in IntClusts 3, 7 and 8. MTS-T1719 (IntClust9) has a clonal inactivating *ARID1A* mutation (CCF=0.93, CI=0.77–1) and a subclonal inactivating *GATA3* mutation (0.69, CI=0.53–0.88). MTS-T1226 (IntClust3) has three functional mutations: a clonal *PIK3CA* mutation (CCF=0.67, CI=0.38–1), a clonal inactivating *MAP3K1* mutation (CCF=0.99, CI=0.73–1) and a subclonal inactivating *MAP3K1* mutation (CCF=0.60, CI=0.41–0.85). The presence of two *MAP3K1* mutations in this instance suggests biallelic *MAP3K1* inactivation in a subset of cells. Overall, there were 199 samples (10.4%) harbouring >1 functional mutation in a Mut-driver gene across the cohort. Multiple mutations in a gene were most frequently observed in *MAP3K1* (53 out of 152 *MAP3K1* mutants with >1 functional mutation; Supplementary Data 1) as previously noted^11^.

### Prognostic associations of mutations in Mut-driver genes

**Figure 6:**
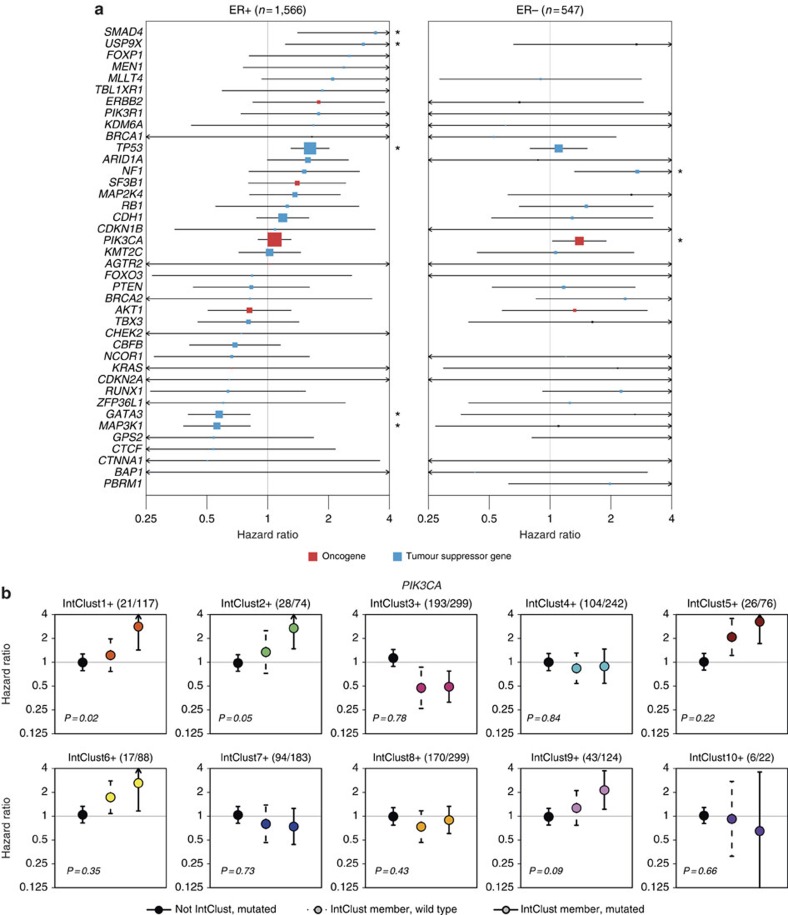
Associations between mutations in the 40 Mut-driver genes and survival. (**a**) Multivariable Cox proportional hazards models were constructed to assess the associations between functional mutations in Mut-driver genes and breast cancer-specific survival (BCSS) in ER+ (left) and ER− (right) cancers. For oncogenes (red), we considered only recurrent mutations, whereas only inactivating mutations were used for tumour suppressor genes (blue). Both classes of mutations were used for *TP53*. The lines represent 95% confidence intervals and sizes of the boxes correspond to the inverse of the interval size. Arrows indicate confidence intervals extending beyond plot range, and ‘' mark genes where mutations are associated BCSS at *P*<0.05. Some genes did not have sufficient mutations in the ER− cohort to obtain a hazard ratio estimate. (**b**) The association between functional *PIK3CA* mutations and BCSS were analyzed in ER+ tumours after stratifying by IntClust. For each IntClust, univariable Cox models were constructed to obtain a hazard ratio estimate for *PIK3CA* mutations in tumours not belonging to the particular IntClust (left; black point, solid line), the effect of IntClust membership for tumours with wild-type *PIK3CA* (middle; coloured point, dashed line), and the simultaneous effects of *PIK3CA* mutation and IntClust membership (right; coloured point, solid line). Lines and arrows represent confidence intervals as in Fig. 6a. The *P* values represent the significance of the interaction between *PIK3CA* mutation and IntClust membership in the Cox model. The fraction of tumours harbouring *PIK3CA* mutations within each IntClust is also indicated in brackets.

To analyze the associations between functional mutations in Mut-driver genes and breast cancer-specific survival (BCSS), we constructed multivariable Cox proportional hazards models (Fig. 6a, Supplementary Table 5) for ER+ and ER− tumours separately, taking into account the effects of patient age, tumour grade, size and lymph node status *(Methods)*. In ER+ tumours, mutations in both *MAP3K1* (hazard ratio (HR)=0.56, CI=0.38–0.82) and *GATA3* (HR=0.58, CI=0.4–0.82) were associated with longer survival. Despite being uncommon, inactivating mutations in *SMAD4* (HR=3.4, CI=1.4–8.3) and *USP9X* (HR=3, CI=1.2–7.2) were associated with worse BCSS. Low levels of nuclear Smad4 and deregulation of other components of the TGF-beta signalling pathway have been associated with poor prognosis in breast cancer^41^. We also observed that inactivating mutations in *NF1* were associated with shorter BCSS in ER− tumours (HR=2.7, CI=1.3–5.5).

*TP53* mutations were associated with worse outcome in ER+ (HR=1.6, CI=1.3–2, *P*=0.0001), but not in ER− disease (HR=1.1, CI=0.8–1.6). Mutations in the DNA-binding domain were associated with the worst outcomes (Supplementary Figs 16). Conversely, mutations in *PIK3CA* were prognostic in patients with ER− tumours (HR=1.4, CI=1.1–1.9), but not in those with ER+ tumours (HR=1.1, CI=0.9–1.3). The association with worse survival in ER− patients was present for *PIK3CA* mutations in both the helical and kinase domains (Supplementary Fig. 17). The number of mutations in Mut-driver genes was not associated with BCSS (Supplementary Fig. 18).

We hypothesized that the prognostic effects of mutations may be influenced by the CNA background. We therefore analyzed the associations between mutations and BCSS after stratifying tumours by IntClust. We tested *PIK3CA* mutations in ER+ cancers as a prototypical example, as previous studies reported contradictory results on the prognostic relevance of *PIK3CA* mutations in ER+ tumours^42^,^43^,^44^. There was no association between mutation domain or mutation CCF and BCSS, and we did not observe differences in patient outcome after stratification by HER2 status (Supplementary Fig. 19). We constructed univariable Cox models with an interaction term to analyze the effects of *PIK3CA* mutations within each IntClust (Fig. 6b). This interaction term provides an indication of whether the presence of mutations within a specific IntClust has an association with BCSS over and beyond the associations of BCSS with IntClust membership or *PIK3CA* mutations in the entire ER+ cohort. Significant interactions were identified in IntClusts1+, 2+ and 9+, suggesting that *PIK3CA* mutations in these specific groups were associated with poor outcome. On the other hand, patients in IntClusts 3+, 4+, 7+ and 8+ with *PIK3CA* mutations had similar outcomes to patients with wild-type *PIK3CA* in their respective groups. These results highlight the importance of genome-based stratification, and suggest that IntClust classification of ER+ tumours might better inform the prognostic and predictive value of *PIK3CA* mutations. Such findings may help interpretation of the results from clinical trials involving phosphoinositide-3-kinase (PI3K) inhibitors^45^. The results of this analysis for *TP53* are shown in Supplementary Fig. 20.

### Intra-tumour heterogeneity across the IntClusts

**Figure 7:**
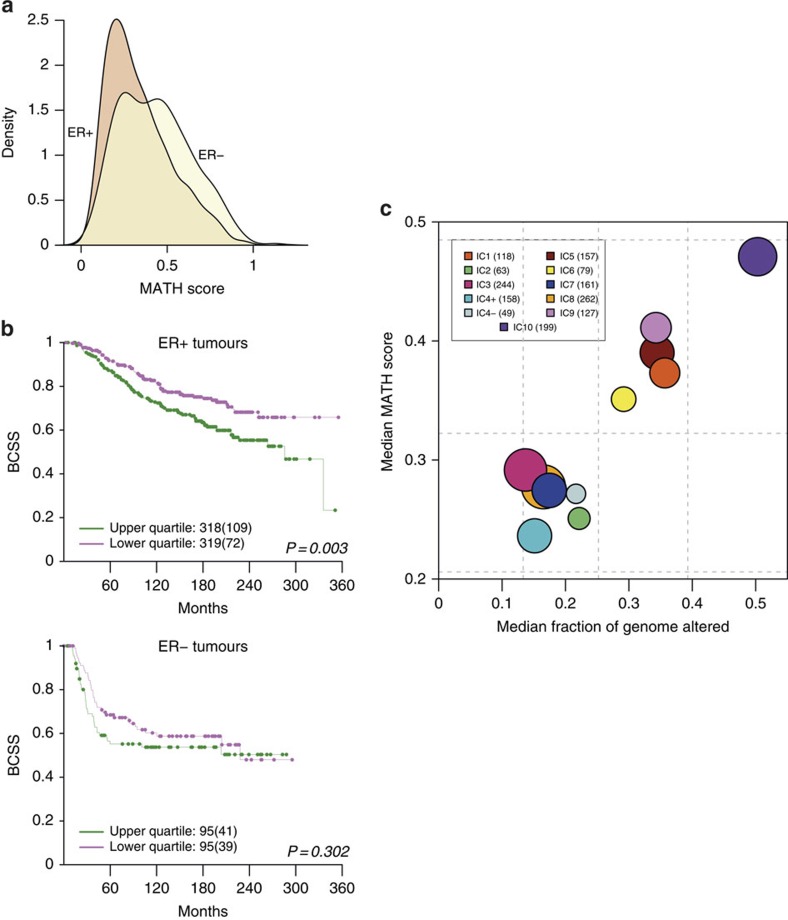
Intra-tumour heterogeneity in breast cancers stratified by IntClust. (**a**) The distributions of mutant-allele tumour heterogeneity (MATH) scores are shown for ER+ and ER− tumours. The score represents a measure of the level of intra-tumour heterogeneity, and was calculated for each tumour as described in Methods. In general, ER+ samples have lower MATH scores than ER− samples, although there are a number of ER+ samples with higher scores. Tumours with fewer than five mutations were excluded from this analysis. (**b**) Kaplan–Meier survival curves (BCSS) are shown for tumours whose MATH scores fall in the lower or upper quartiles of the ER+ (top) and ER− (bottom) distributions. The numbers of samples under consideration are indicated, and the numbers in brackets represent the deaths occurring in each cohort. (**c**) Bubble plot of median MATH scores and CIN scores for each IntClust. The CIN is a measure of the percentage of the genome altered by CNAs. Dashed lines depict the quartiles for both scores (vertical lines, CIN quartiles; horizontal lines, MATH score quartiles) in the cohort as a whole. The areas of the circles are proportional to number of samples in each IntClust.

To quantify intra-tumour heterogeneity, we used the previously established mutant-allele tumour heterogeneity (MATH) score^46^, which is based on the variation in VAFs of all mutations in a tumour (Supplementary Table 6). We excluded samples with fewer than five somatic mutations from this analysis, and computed the MATH scores for the ER+ and ER− tumours separately (Fig. 7a). ER+ tumours generally had lower MATH scores (median=0.29, IQR=0.18–0.44) than ER− tumours (median=0.41, IQR=0.25–0.56). Higher MATH scores were associated with worse outcome in ER+ cancers (Fig. 7b).

The relationship between intra-tumour heterogeneity and chromosomal instability (CIN; defined as the percentage of the genome affected by CNAs) across the IntClusts is depicted in Fig. 7c. Tumours within IntClust10 had the highest MATH scores (median=0.47, IQR=0.31–0.61). Although IntClusts 4- and 10 both comprise triple-negative basal-like tumours, tumours in IntClust4-, which have high levels of lymphocytic infiltration, had low MATH scores (median=0.27, IQR=0.19–0.54). Tumours belonging to the IntClusts with the best outcomes (Supplementary Fig. 10a) had low scores (IntClust3: median=0.29, IQR=0.17–0.45; IntClust7: median=0.27, IQR=0.29–0.40; IntClust8: median=0.28, IQR=0.18–0.39). Patients within IntClusts 1 (median=0.37, IQR=0.26–0.53), 6 (median=0.35, IQR=0.21–0.48) and 9 (median=0.41, IQR=0.25–0.58) have poor outcomes, and their tumours had intermediate MATH scores. Surprisingly, tumours in IntClust2 had low MATH scores (median=0.25, IQR=0.16–0.37) despite patients in this subgroup having poor outcomes. The 11q13–14 amplicon (two gene cassettes centred around *CCND1* and *PAK1*, respectively) is a key driver CNA in IntClust2, and may be responsible for the poor clinical trajectories of patients within this group.

## Discussion

Understanding the clinical implications of driver mutations in breast cancer has been hindered by small sample sizes and lack of long-term clinical follow-up. The large data set reported here indicates that patient classification into clinically relevant subtypes requires profiling both gene mutations (by sequencing) and stratification based on CNAs, which dominate the genomic landscape. The results (available at www.cbioportal.org) represent an important resource characterizing the combined genomic profiles of a large number of primary breast tumours from patients with long-term follow-up data.

A key aim of precision cancer medicine is to tailor clinical management based on the specific events that are relevant to tumour development and progression. To this end, it is important to comprehensively catalogue the drivers for a particular cancer, and to also determine the contexts that define their relevance. We used a ratiometric method to identify 40 Mut-driver genes, and noted that ER stratification revealed differences in the oncogenic properties of some genes, including *SMAD4* and *ERBB2*. Novel genes on the Mut-driver list include *FOXO3*, a transcription factor regulated by Akt signalling, and *AGTR2*, which may be a therapeutic target^47^. The ratiometric method identified Mut-drivers in breast cancers that were previously known drivers in other cancer types (such as *KRAS, ARID1A*, *CDKN2A*, *PBRM1*, *KDM6A*, *MEN1*, *FOXP1*, *USP9X*, *BAP1*, *SMAD4*). This raises the possibility that therapies used in other clinical settings may be applicable to breast cancers with mutations in these genes. For example, mutations in the SWI/SNF components *ARID1A* and *PBRM1* are especially prevalent in bladder^48^ and renal^49^ cancers, and tumours harbouring these mutations may have dependencies on other SWI/SNF components that can be therapeutically exploited^50^. Previous work has shown that disruption of *ARID1B* function in an *ARID1A*-deficient context inhibits proliferation in a number of cancer cell lines^51^.

The degree of inter-tumour heterogeneity present in breast cancer, and ER+ disease in particular, at the genomic^3^, transcriptomic^3^,^52^ and clinical^2^ levels has long been recognized. The IntClust stratification of ER+ tumours into groups with specific CNAs and distinct prognostic courses has been robustly validated^6^. Here we provide further biological insight into this inter-tumour heterogeneity by overlaying the mutation frequencies of 40 breast cancer Mut-driver genes, and by characterizing their clonal states. For example, *KMT2C* mutations are infrequent and usually subclonal in IntClust1, but more common and usually clonal in IntClust8. IntClust10 ER+ tumours had a relatively high prevalence of *TP53* mutations. This observation supports the CNA-based IntClust classification of some ER+ cancers into a subtype containing mostly triple-negative basal-like tumours.

The observation that *PIK3CA* mutations have distinct prognostic associations in ER+ tumours stratified into IntClusts is a key novel finding. Recent reports of mouse models have demonstrated that the same *PIK3CA* mutation can result in different tumour phenotypes depending on cellular background^53^,^54^, and our results may similarly be due to the specific biological properties of tumours within specific IntClusts. While confirmation in further studies is required, these results provide compelling evidence for genome-based stratification in future therapeutic trials of PI3K inhibitors.

The success of targeted therapies, however, will depend on overcoming the problem of resistance, which may occur earlier in tumours with more clonal diversity^55^. We used the MATH score as a measure of intra-tumour heterogeneity and observed that higher MATH scores were associated with reduced survival in ER+ tumours. However, tumours belonging to IntClust2, which comprises patients with poor outcome, have low levels of intra-tumour heterogeneity and CIN. These tumours are characterized by co-amplification of *CCND1* and *PAK1* at 11q13–14, and have previously been shown to be resistant to neo-adjuvant cytotoxic chemotherapy^6^. These observations will need to be externally validated, and should be taken forward to develop better strategies to manage patients whose tumours belong to this small but highly aggressive subgroup.

In conclusion, combining copy number, gene expression and mutation profiles provides a richer understanding of the genomic landscape of breast cancer, and offers new insights into inter- and intra-tumour heterogeneity that should inform the future development of clinical management of patients.

## Methods

### Sample collection

We sequenced a total of 2,433 primary tumours and 650 normal non-cancerous samples comprising normal adjacent breast tissue (523) or peripheral blood cells (127). Overall, there were 548 matched tumour/normal pairs present in our data set. We also sequenced replicates for 221 primary tumours, as well as a number of commonly used breast cancer cell lines.

All samples were obtained with the consent from patients and appropriate approval from ethical committees (REC ref 07/H0308/161; REC ref 12/EE/0484; REC ref 07/Q0106/63). Detailed information about tissue collection for each cohort can be found in their respective publications: METABRIC^3^, NeoTango^56^, Nottingham^57^ and DETECT^58^. Clinical data from the original studies were updated with the latest available records.

### Histological classification and ER status

Information on lymph node status, stage and tumour size was available from original histopathology reports for all studies. Expert breast cancer pathologists reviewed FFPE sections stained with haematoxylin and eosin (H&E) from tumours with available material and scored histological tumour type, grade, tumour cellularity and lymphocytic infiltration.

Immunohistochemistry-based (IHC) scoring of ER status was, where available, used to classify ER−positive (ER+) and ER−negative (ER−) tumours. To confirm this classification for samples which had gene expression data available, we fit a two-component Gaussian mixture model to the expression levels of *ESR1* using the mixtools package^59^ in R, and computed the probabilities of the samples belonging to the two distributions defined by the components. The distribution yielding the higher probability was selected to represent the ER status for each sample. Where the calls between the two systems differed, we used the expression-derived classification if the probability of belonging to the opposite distribution was at least 5 × higher than for the distribution described by IHC; this scheme was chosen so as to assign more weight to the IHC classification, as this is currently the clinical gold standard. We performed a similar analysis with *ERBB2* expression levels to corroborate the IHC-based HER2 calls. For patients without expression data (*n*=416), we used the IHC scores to assign ER and HER2 status. Similarly, gene expression-based classification was used for samples without IHC data.

### DNA extraction

Sample processing, DNA extractions and quality assessment were based on the protocols described in the METABRIC publication^3^.

For UK samples DNA was extracted from 10 30-μm sections from each tumour using the DNeasy Blood & Tissue Kit (Qiagen, UK) on the QIAcube (Qiagen) according to manufacturer's instructions.

For CA samples DNA was extracted from 10–20 8-μm sections from each tumour using the MagAttract DNA M48 Kit (Qiagen) on the BioRobot M48 (Qiagen) according to manufacturer's instructions. DNA was quantified with the Qubit Fluorometer (Thermo Fisher Scientific, MA, USA) and quality assessed by gel electrophoresis.

### Library preparation and sequencing

DNA was quantified using Qubit HS dsDNA assay (Life Technologies, CA) and libraries were prepared from a total of 50 ng of DNA using Illumina's Nextera Custom Target Enrichment kit (Illumina, CA). In brief, a modified Tn5 transposase was used to simultaneously fragment DNA and attach a transposon sequence to both end of the fragments generated. This was followed by a limited cycle PCR amplification (11 cycles) using barcoded oligonucleotides that have primer sites on the transposon sequence generating 96 uniquely barcoded libraries per run. The libraries were then diluted and quantified using Qubit HS dsDNA assay.

Five hundred nanograms from each library were pooled into a capture pool of 12 samples. Enrichment probes (80-mer) were designed and synthesized by Illumina; these probes were designed to enrich for all exons of the target genes, as well for 500 bp up- and downstream of the gene. The capture was performed twice to increase the specificity of the enrichment. Enriched libraries were amplified using universal primers in a limited cycle PCR (11 cycles). The quality of the libraries was assessed using Bioanalyser (Agilent Technologies, CA) and quantified using KAPA Library Quantification Kits (Kapa Biosystems, MA).

Products from four capture reactions (that is, 48 samples) were pooled for sequencing in a lane of Illumina HiSeq 2,000. Sequencing (paired-end, 100 bp) of samples and demultiplexing of libraries was performed by Illumina (Great Chesterford, UK).

### Alignment and quality assessment

The sequenced reads were aligned with Novoalign, and the resulting BAM files were preprocessed using the GATK Toolkit^60^. Sequencing quality statistics were obtained using the GATK's DepthOfCoverage tool and Picard's CalculateHsMetrics. Coverage metrics are presented in Supplementary Fig. 1. Samples were excluded if <25% of the targeted bases were covered at a minimum coverage of 50 × .

The identities of those samples with copy number array data available were confirmed by analyzing the samples' genotypes at loci covered by the Affymetrix SNP6 array. Genotype calls from the sequencing data were compared with those from the SNP6 data that was generated for the original studies. This was to identify possible contamination and sample mix-ups, as this would affect associations with other data sets and clinical parameters.

### Variant calling

To identify all variants in the samples, we used MuTect^61^ (without any filtering) for SNVs and the Haplotype Caller^60^ for indels. All reads with a mapping quality <70 were removed prior to calling. Variants were annotated with ANNOVAR^62^ using the genes' canonical transcripts as defined by Ensembl (Supplementary Data 1). Custom scripts were written to identify variants affecting splice sites using exon coordinates provided by Ensembl. Indels were referenced by the first codon they affected irrespective of length; for example, insertions of two bases and five bases at the same codon were classed together.

To obtain the final set of mutation calls, we used a two-step approach, first removing any spurious variant calls arising as a consequence of sequencing artefacts (generic filtering) and then making use of our normal samples and the existing data to identify somatic mutations (somatic filtering). For both levels of filtering, we used hard thresholds that were obtained, wherever possible, from the data itself. For example, some of our filtering parameters were derived from considering mutations in technical replicates (15 samples sequenced in triplicate)^63^. We compared the distributions of key parameters (including quality scores, depth, VAF) for concordant (present in all three replicates) and discordant (present in only one out of three replicates) variants to obtain thresholds, and used ROC analysis to select the parameters that best identified concordant variants.

### SNV filtering

- Based on our analysis of replicates, SNVs with MuTect quality scores <6.95 were removed.
- We removed those variants that overlapped with repetitive regions of *MUC16* (chromosome 19: 8,955,441–9,044,530). This segment contains multiple tandem repeats (mucin repeats) that are highly susceptible to misalignment due to sequence similarity.
- Variants that failed MuTect's internal filters due to ‘nearby_gap_events' and ‘poor_mapping_regional_alternate_allele_mapq' were removed.
- Fisher's exact test was used to identify variants exhibiting read direction bias (variants occurring significantly more frequently in one read direction than in the other; FDR=0.0001). These were filtered out from the variant calls.
- SNVs present at VAFs smaller than 0.1 or at loci covered by fewer than 10 reads were removed, unless they were also present and confirmed somatic in the Catalogue of Somatic Mutations in Cancer (COSMIC). The presence of well-known *PIK3CA* mutations present at low VAFs was confirmed by digital PCR (see below), and supported the use of COSMIC when filtering SNVs.
- We removed all SNVs that were present in any of the three populations (AMR, ASN, AFR) in the 1,000 Genomes study (Phase 1, release 3) with a population alternate allele frequency of ⩾1%.
- We used the normal samples in our data set (normal pool) to control for both sequencing noise and germline variants, and removed any SNV observed in the normal pool (at a VAF of at least 0.1). However, for SNVs present in more than two breast cancer samples in COSMIC, we used more stringent thresholds, removing only those that were observed in >5% of normal breast tissue or in >1% of blood samples. The different thresholds were used to avoid the possibility of contamination in the normal pool affecting filtering of known somatic mutations. This is analogous to the optional ‘panel of normals' filtering step used by MuTect in paired mode, in which mutations present in normal samples are removed unless present in a list of known mutations^61^.

### Indel filtering

- As for SNVs, we removed all indels falling within tandem repeats of *MUC16* (coordinates given above).
- We removed all indels deemed to be of ‘LowQual' by the Haplotype Caller with default parameters (Phred-scaled confidence threshold=30).
- As for SNVs, we removed indels displaying read direction bias. Indels with strand bias Phred-scaled scores ⩾40 were removed.
- We downloaded the Simple Repeats and Microsatellites tracks from the UCSC Table Browser^14^, and removed all indels overlapping these regions. We also removed all indels that overlapped homopolymer stretches of six or more bases.
- As for SNVs, indels were removed if present in the 1,000 Genomes database at an allele frequency >1%, or if they were present in normal samples in our data set. Thresholds were adjusted as for SNVs if the indel was present in COSMIC. The same thresholds for depth and VAF were used.

### Pathogenic germline variants

We used the ClinVar database^15^ to identify pathogenic germline mutations, using only those SNVs and indels recorded as being ‘probable-pathogenic' or ‘pathogenic', and ‘germline', ‘inherited', ‘paternal', ‘maternal', ‘biparental' or ‘uniparental'. Variants classified as ‘germline' by the unpaired pipeline were classified as ‘pathogenic' using the ClinVar annotation, unless they were also present at allele frequencies >1% in the 1,000 Genomes resource.

In addition, we classified SNVs absent in ClinVar but present in between one and six (1%) normal samples as ‘pathogenic' if they were either inactivating (truncating or affecting splice sites), or identified as being ‘deleterious' or ‘damaging' by Provean^64^ Pathogenic indels present in one to six normal samples but absent from ClinVar were classified as ‘pathogenic' if they were predicted to disrupt the reading frame or disrupt a splice junction.

### *TP53* Sanger sequencing

To assess the sensitivity and specificity of our variant calls, we compared a large number of *TP53* variants called in our study with those from Sanger sequencing (SS). In a previous study, we studied the mutation spectrum of *TP53* in primary breast tumours from METABRIC^12^. Details of the exons sequenced and primers used can be found in Supplementary Table 7. A total of 1,523 cases were evaluated. Of the remaining variants, there was 93% concordance between the 2 sequencing projects (988 wild type in both studies, 429 mutants in both studies). There were 40 cases (3.9%) for which a *TP53* mutation was identified by SS but not in our study, and we identified 66 cases (4.6%) with *TP53* mutations that were not previously called. We note that the increased sensitivity of next-generation sequencing and the depth of coverage obtained for this study may have helped identify variants that were either not identified or not called in the SS project.

### *PIK3CA* digital PCR

To ensure that hotspot variants with low VAF (range: 2–10%) that we retained are true (see variant filtering above), we performed digital TaqMan-based quantitative PCR on 23 cases with *PIK3CA* H1047R mutations using the BioMark qdPCR 37 K Digital Array Chip (Fluidigm, CA). Briefly, 3.5 μl of template DNA was mixed with specific concentrations of primer and probes in the presence of 1 × TaqMan Universal PCR Master Mix (Thermo Fisher Scientific) and 1 × sample loading buffer. The cycling profile was as follows: initiation at 50 °C for 2 min and incubation at 95 °C for 10 min followed by 60 cycles of 95 °C for 15 s and 60 °C for 1 min. The data were analyzed with the Digital PCR Analysis Software v4.0.1 (Fluidigm). In each panel of the chip, we diluted the sample to ensure that there was not more than an average of one template molecule. The quantitative performance of the platform was assessed by using serial twofold dilutions of HCC1954, a breast cancer cell line known to carry the H1047R *PIK3CA* mutation. Each sample was analyzed in duplicate. All 23 cases were positive for *PIK3CA* H1047R mutations with mutations frequency detected by digital PCR ranging 1–17%.

### Copy number analyses

The SNP6 data for 2,087 tumours were reanalysed using ASCAT^65^ to obtain segmented copy number calls and estimates of tumour ploidy and purity. Somatic CNAs were obtained by removing germline CNVs as defined in the original METABRIC study^3^. We defined regions of LOH as those in which there were no copies present of either the major or minor allele, irrespective of total copy number. Recurrent CNAs were identified with GISTIC2 (ref. 66), with log_2_ ratios obtained by dividing the total number of copies by tumour ploidy for each ASCAT segment. Thresholds for identifying gains and losses were set to 0.4 and (−)0.5, respectively; these values were obtained by examining the distribution of log_2_ ratios to identify peaks associated with copy number states. A broad length cut-off of 0.98 was used, and peaks were assessed to rule out probe artefacts and CNVs that may have been originally missed. The percentage of each tumour genome altered was computed after correcting for tumour ploidy.

### Survival analyses

Univariable or multivariable Cox proportional hazards models were used to examine the association between mutations and survival. BCSS was used as the endpoint. Patients with deaths due to other or unknown causes were censored at the date of death, and all other patients were censored at the date of last contact. For the multivariable models, we included as variables: grade size (greater or less than 50 mm), lymph node status (positive or negative) and age (greater or less than 55). This coding scheme was chosen taking into consideration the minimum level of data available across the cohorts in the study. The same scheme was used when performing logistic regression for identifying associations between clinical parameters and mutation presence. To identify associations between events, we used Fisher's exact test for 2 × 2 contingency tables.

### Driver gene identification

To identify genes that may be relevant to breast cancer, we looked for those that harboured multiple recurrent or inactivating mutations, as these are mutation patterns typical of oncogenes and tumour suppressors. Recurrent mutations were defined as missense SNVs and in-frame substitutions that affected the same codon of the annotation transcript, whereas inactivating mutations included nonsense SNVs, frameshift substitutions and variants that affected splice sites. The proportions of recurrent (ONC) and inactivating (TSG) mutations for each gene (out of the total number of mutations) were computed, and a threshold of 0.2 was used (20/20 rule). Genes with an ONC score >0.2 and with a TSG score >0.05 were classified as tumour suppressors. A minimum of five recurrent or inactivating mutations was required for a gene to be selected as putative drivers. The method was adapted from the study by Vogelstein *et al*.^16^

### Clonal states of Mut-driver mutations

To infer the CCFs in which the observed mutations occurred, we used VAFs, and copy number data and tumour purity estimates as obtained from ASCAT. The CCF was defined as in ref. 40:


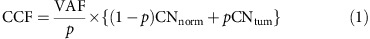


where *p* is the tumour purity estimate, and CN_norm_ and CN_tum_ refer to the normal (that is, 2) and tumour copy number states, respectively. We used the ‘binconf' function in the R package Hmisc (http://CRAN.R-project.org/package=Hmisc) to obtain an estimate and CI for the VAF given the depth and the number of reads harbouring the mutant allele at the mutant locus.

To identify ‘clonal' (present in the entire tumour) or ‘subclonal' (present in only a subset of tumour cells) mutations, we computed 95% CIs for each CCF estimate based on the VAF and depth of coverage at the mutation loci. Mutations were labelled as ‘subclonal' if their CCF CIs did not overlap 1 (ref. 40).

### Intra-tumour heterogeneity

To quantify the level of intra-tumour heterogeneity present in a sample, we used the MATH measure described in ref. 46:


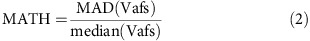


where MAD(Vafs) is the median absolute deviation of the VAFs of all the mutations (coding and noncoding) in a tumour.

### Data availability

All primary data (BAM files) are deposited at the European Genome-phenome Archive (EGA) under study accession number EGAS00001001753, and may be downloaded on request and authorization by the METABRIC Data Access Committee. Somatic mutation calls and ASCAT segment files for 2,433 primary tumours are available at http://github.com/cclab-brca, which also hosts the code used to perform key analyses and produce the figures. A matrix of coding mutations is provided as Supplementary Dataset 3. Clinical data (tumour morphology, ER and HER2 status, patient characteristics, treatment, follow-data, metastasis data and relapse data), gene expression data, copy number data and somatic mutations data for the 1980 patients from the original METABRIC publication can be found on cBioPortal, and are freely available.

## Additional information

**How to cite this article:** Pereira, B. *et al*. The somatic mutation profiles of 2,433 breast cancers refines their genomic and transcriptomic landscapes. *Nat. Commun.* 7:11479 doi: 10.1038/ncomms11479 (2016).

## Supplementary Material

Supplementary Information Supplementary Figures 1-21, Supplementary Tables 1-7 and Supplementary Reference.

Supplementary Data 1 Details of genes & mutations in this study.

Supplementary Data 2 Percentages of samples with coding mutations and ONC and TSG scores for genes in various cohorts.

Supplementary Data 3 Matrix for mutations across all genes and samples. NA=no coding mutation. For inframe indels and missense SNVs, the distinction between recurrent and non-recurrent events is made.
