# Erratum: The somatic mutation profiles of 2,433 breast cancers refine their genomic and transcriptomic landscapes

**DOI:** 10.1038/ncomms11908

**Published:** 2016-06-06

**Authors:** Bernard Pereira, Suet-Feung Chin, Oscar M. Rueda, Hans-Kristian Moen Vollan, Elena Provenzano, Helen A. Bardwell, Michelle Pugh, Linda Jones, Roslin Russell, Stephen-John Sammut, Dana W. Y. Tsui, Bin Liu, Sarah-Jane Dawson, Jean Abraham, Helen Northen, John F. Peden, Abhik Mukherjee, Gulisa Turashvili, Andrew R. Green, Steve McKinney, Arusha Oloumi, Sohrab Shah, Nitzan Rosenfeld, Leigh Murphy, David R. Bentley, Ian O. Ellis, Arnie Purushotham, Sarah E. Pinder, Anne-Lise Børresen-Dale, Helena M. Earl, Paul D. Pharoah, Mark T. Ross, Samuel Aparicio, Carlos Caldas

Nature Communications 7 Article number:1147910.1038/ncomms11479 (2016); Published: 05102016; Updated: 06062016.

The original version of this Article contained an error in the spelling of ‘refine' in the title of the paper. This has now been corrected in both the PDF and HTML.

