# Supplementary material for: The somatic mutation profiles of 2,433 breast cancers refines their genomic and transcriptomic landscapes
Source: Nat Commun. 2016 May 10;7:11479. doi: 10.1038/ncomms11479 (PMC4866047; doi:10.1038/ncomms11479)
Supplement: Supplementary Information — Supplementary Figures 1-21, Supplementary Tables 1-7 and Supplementary Reference. [file ncomms11479-s1.pdf]

Supplementary Fig. 1

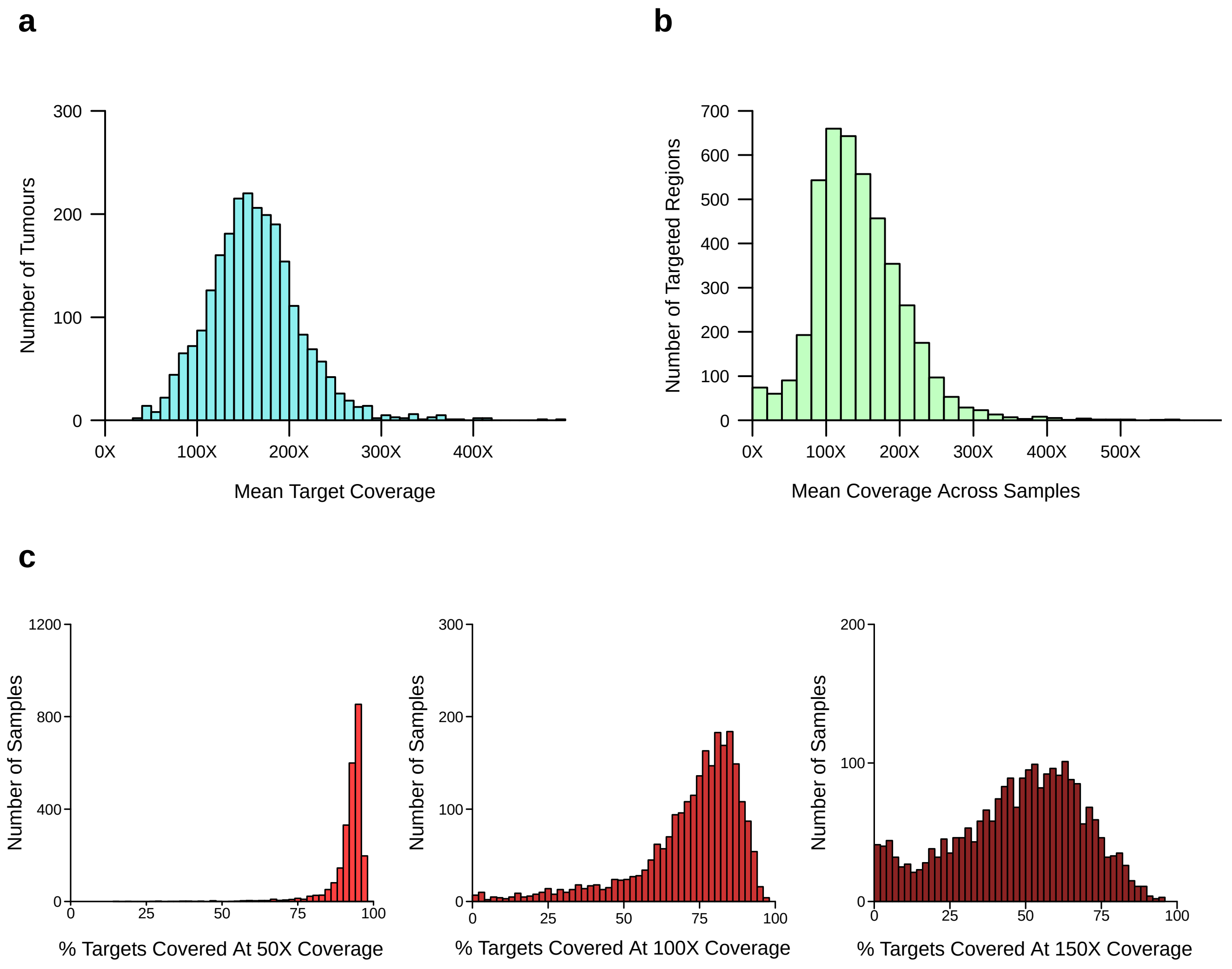

Supplementary Figure 1 – Sequencing of 173 genes in 2433 tumours using the Illumina Nextera Custom Enrichment Kit.

We used the Genome Analysis Toolkit (GATK's) DepthOfCoverage tool to calculate the coverage obtained in the targeted regions for each tumour sample. Only reads with high mapping quality scores were used (***Methods***). The histograms show:

a) The mean depth of coverage across the targeted regions for each sample.

b) The mean coverage obtained for each of the targeted regions across all samples.

c) The percentages of targeted bases covered at 50X, 100X or 150X across all samples.

Supplementary Fig. 2

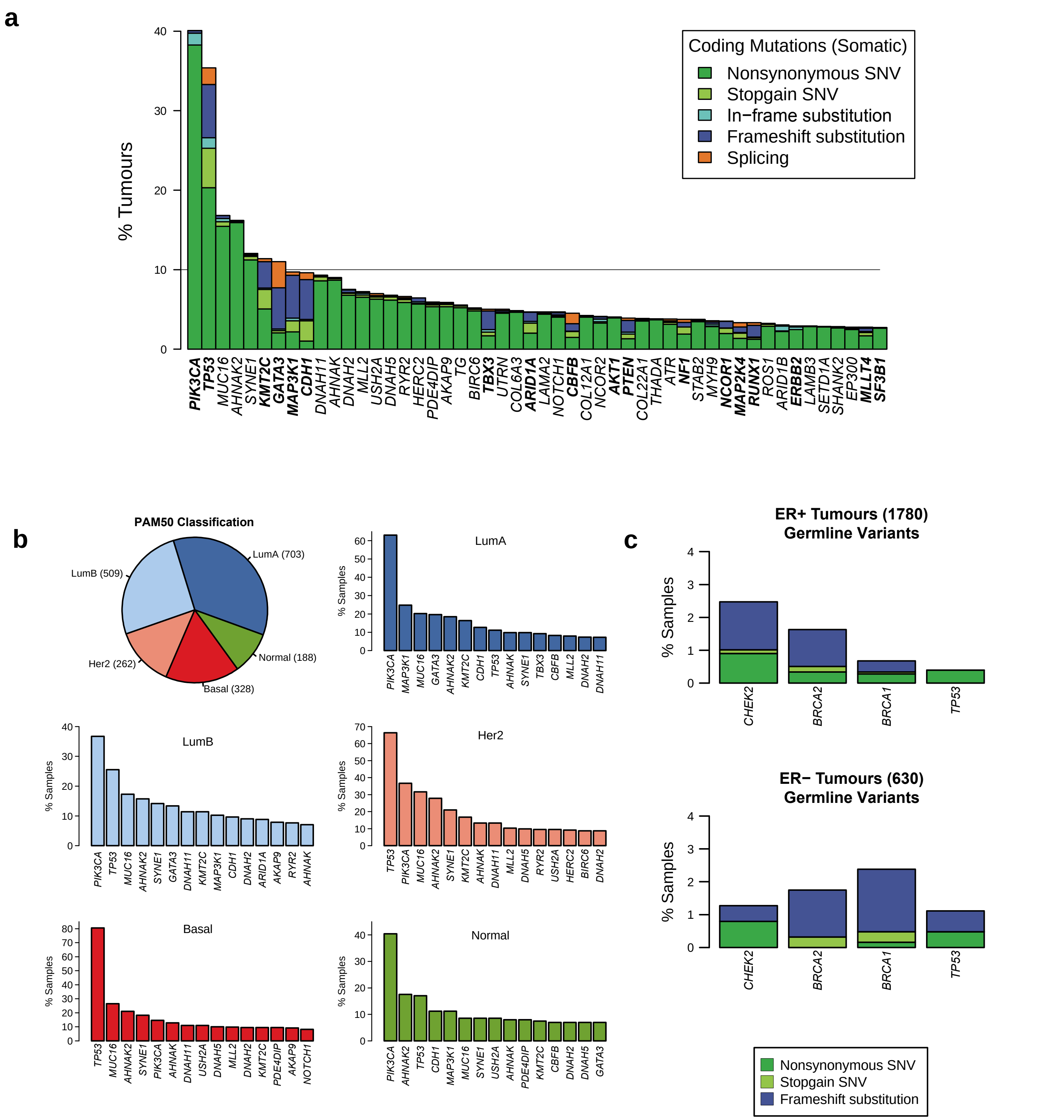

Supplementary Figure 2 – Frequently mutated genes in 2433 primary tumours.

a) The percentages of samples with coding mutations (predicted to affect the protein sequence) in the 50 most frequently mutated genes in our panel of 173 genes. Splicing variants refer to mutations (indels and point mutations) that disrupt a canonical splice site. SNV=single nucleotide variant. Gene names in bold indicate Mut-driver genes (see main text). The 10% frequency is highlighted as a reference.

b) Fractions of samples with mutations in the 15 most frequently mutated genes (based on 173 genes sequenced) across the PAM50 subtypes. The numbers of samples within the subtypes is also shown.

c) Pathogenic variants for *BRCA1*, *BRCA2*, *TP53* and *CHEK2* in ER+ and ER- cancers.

Supplementary Fig. 3

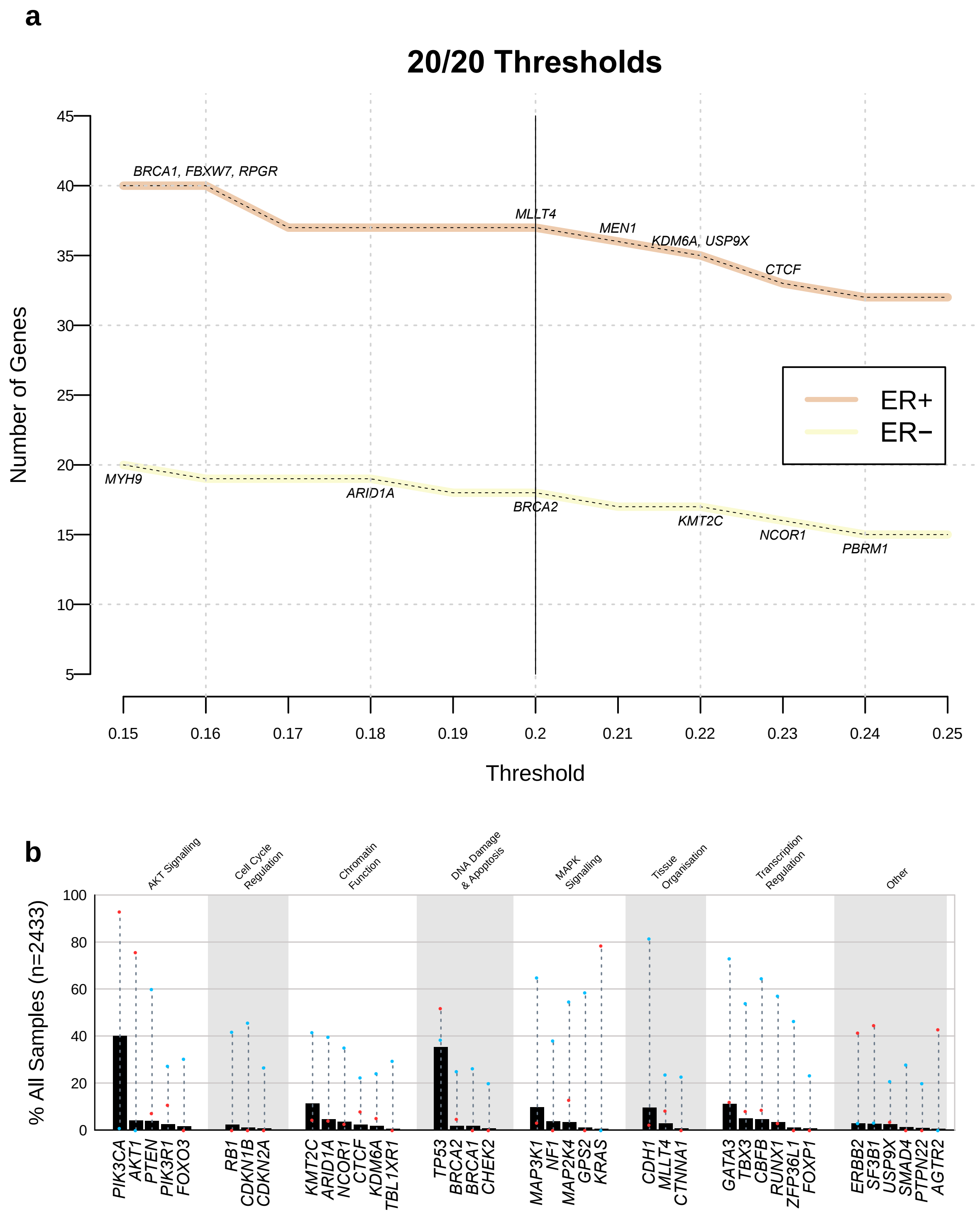

Supplementary Figure 3 – Identifying genes using ratiometric method.

a) Effect of varying the cutoff on the number of genes identified as Mut-drivers. Analyses performed separately for ER+ (n=1780) and ER- (n=635) tumours. Genes that dropped out as the stringency was increased are indicated on the plot. The threshold used (0.2) is indicated by the vertical line.

b) Plot as in ***Figure 1*** but for all samples. Red and blue dots indicate oncogene and tumour suppressor gene scores. Black bars indicate the proportions of samples carrying mutations.

Supplementary Fig. 4

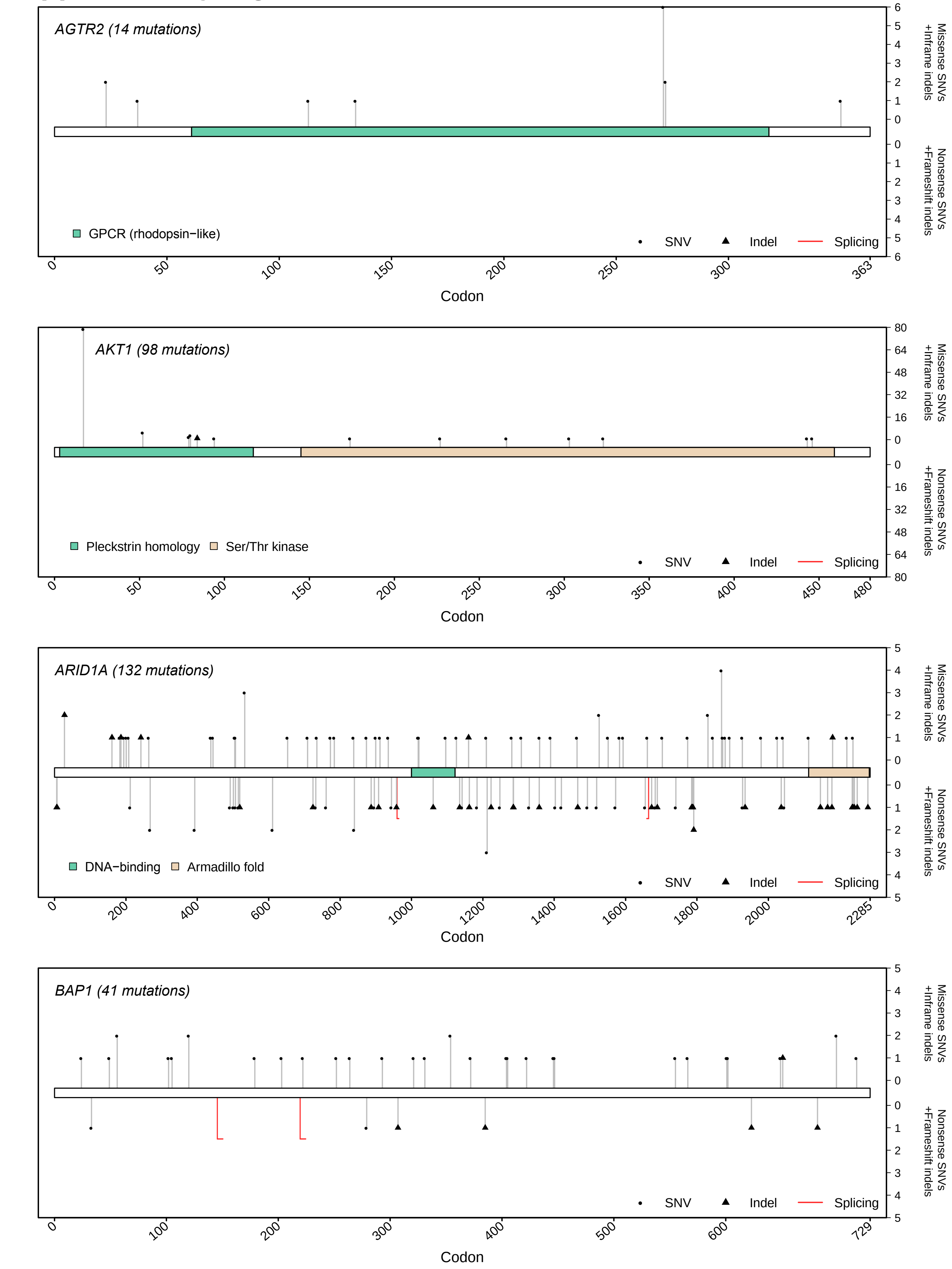

Supplementary Figure 4 – Mutation spectra of the Mut-driver genes

Lollipop plots showing the mutation spectra for 40 Mut-driver genes across their protein domains. Mutations below the gene are inactivating, whereas mutations above the gene indicate nonsynonymous SNVs and in-frame indels. Mutations in splice sites are shown by the red lines below the bar. Splice mutations pointing towards the left are in acceptor sites, and those pointing towards the right are in donor sites. The plots are ordered alphabetically by gene name.

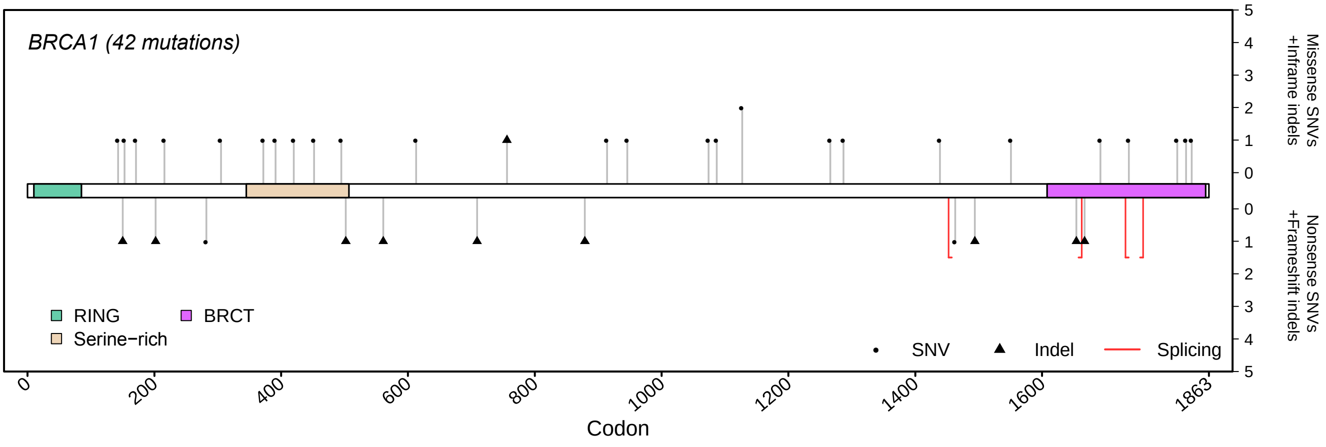
BRCA1 (42 mutations)
RING
BRCT
Serine−rich
SNV
Indel
Splicing
Missense SNVs +Inframe indels
Nonsense SNVs +Frameshift indels
0 200 400 600 800 1000 1200 1400 1600 1863
Codon

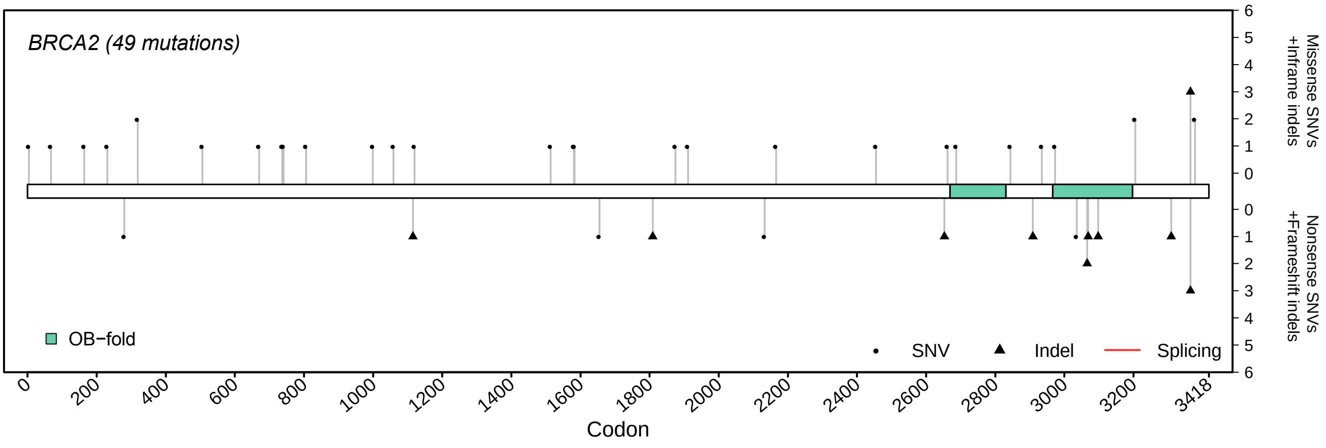
BRCA2 (49 mutations)
OB−fold
SNV
Indel
Splicing
Missense SNVs +Inframe indels
Nonsense SNVs +Frameshift indels
0 200 400 600 800 1000 1200 1400 1600 1800 2000 2200 2400 2600 2800 3000 3200 3418
Codon

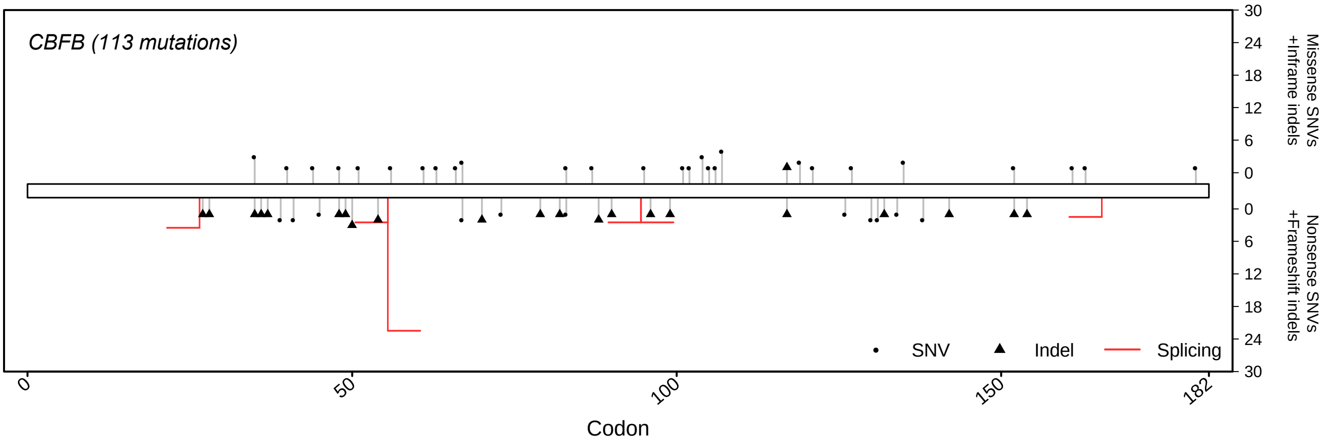
CBFB (113 mutations)
SNV
Indel
Splicing
Missense SNVs +Inframe indels
Nonsense SNVs +Frameshift indels
0 50 100 150 182
Codon

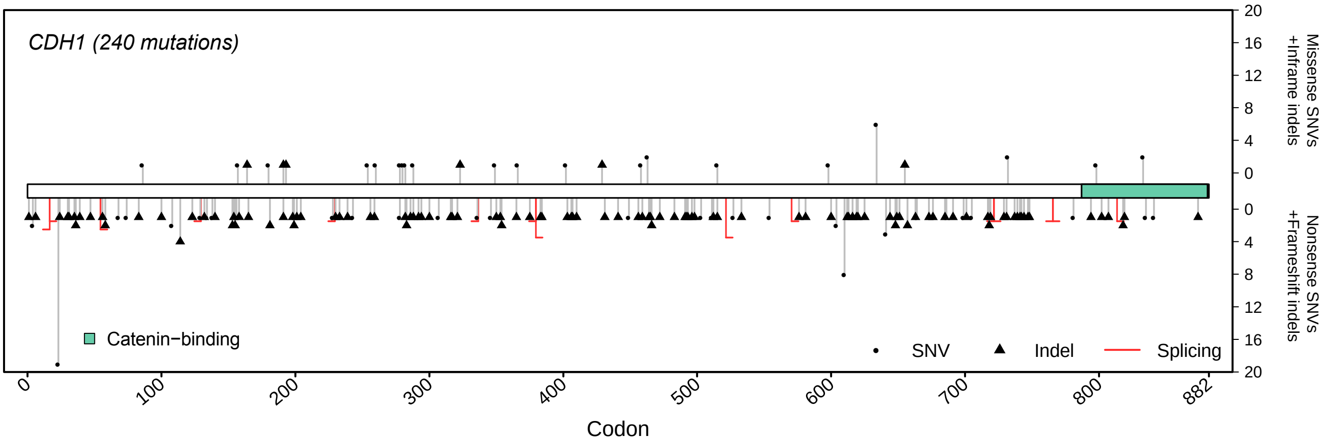
CDH1 (240 mutations)
Catenin−binding
SNV
Indel
Splicing
Missense SNVs +Inframe indels
Nonsense SNVs +Frameshift indels
0 100 200 300 400 500 600 700 800 882
Codon

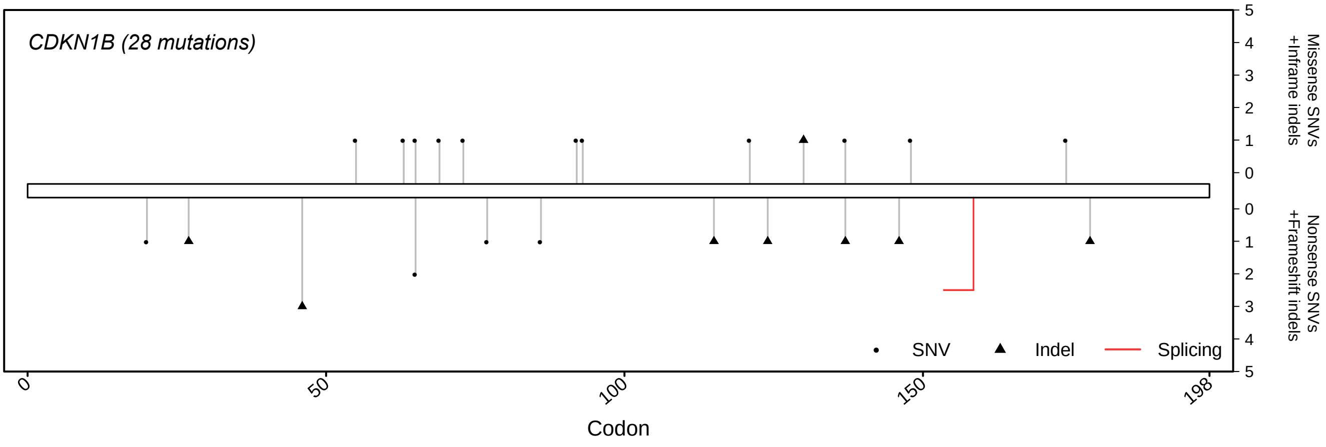
CDKN1B (28 mutations)
Missense SNVs +Inframe indels
Nonsense SNVs +Frameshift indels
SNV
Indel
Splicing
0
50
100
150
198
Codon

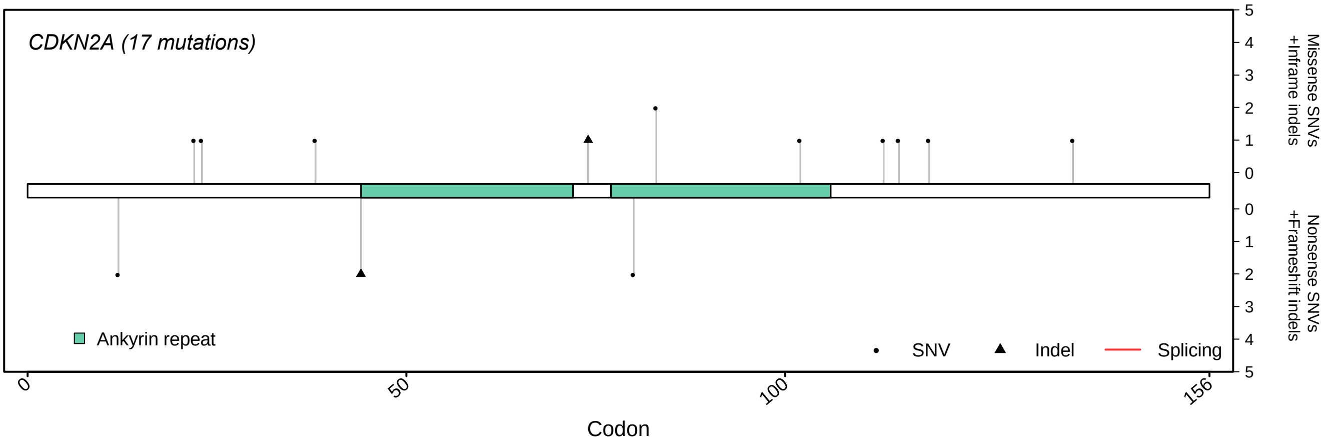
CDKN2A (17 mutations)
Missense SNVs +Inframe indels
Nonsense SNVs +Frameshift indels
Ankyrin repeat
SNV
Indel
Splicing
0
50
100
156
Codon

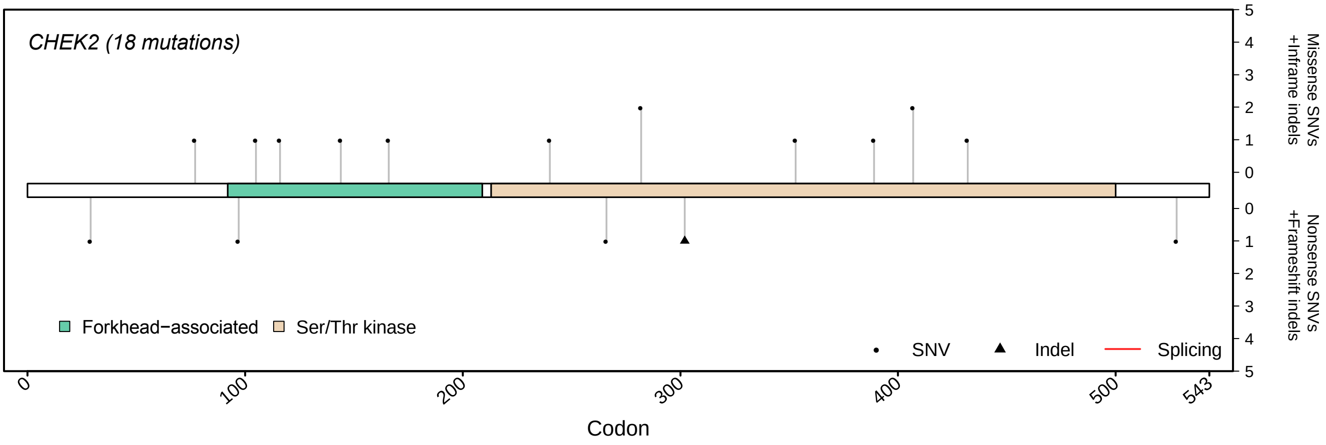
CHEK2 (18 mutations)
Missense SNVs +Inframe indels
Nonsense SNVs +Frameshift indels
Forkhead−associated
Ser/Thr kinase
SNV
Indel
Splicing
0
100
200
300
400
500
543
Codon

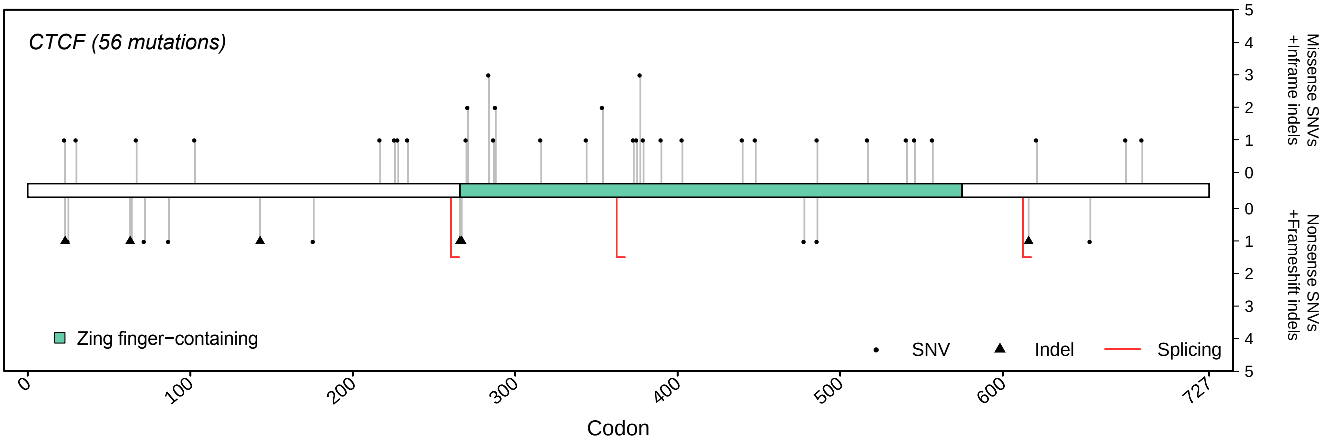
CTCF (56 mutations)
Missense SNVs +Inframe indels
Nonsense SNVs +Frameshift indels
Zing finger−containing
SNV
Indel
Splicing
0
100
200
300
400
500
600
727
Codon

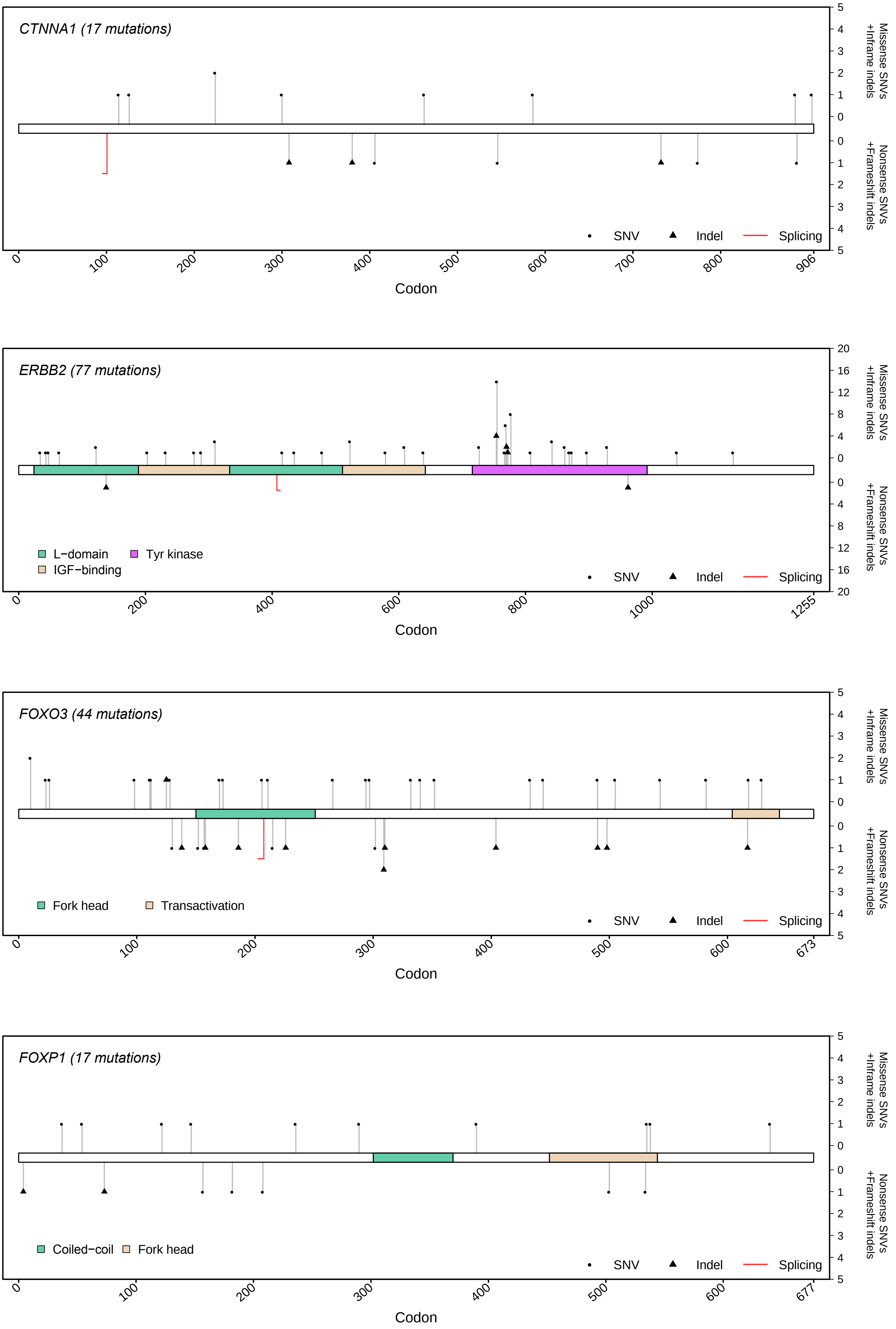

CTNNA1 (17 mutations)
Missense SNVs +Inframe indels
Nonsense SNVs +Frameshift indels
SNV
Indel
Splicing
Codon
ERBB2 (77 mutations)
L−domain
Tyr kinase
IGF−binding
FOXO3 (44 mutations)
Fork head
Transactivation
FOXP1 (17 mutations)
Coiled−coil
Fork head

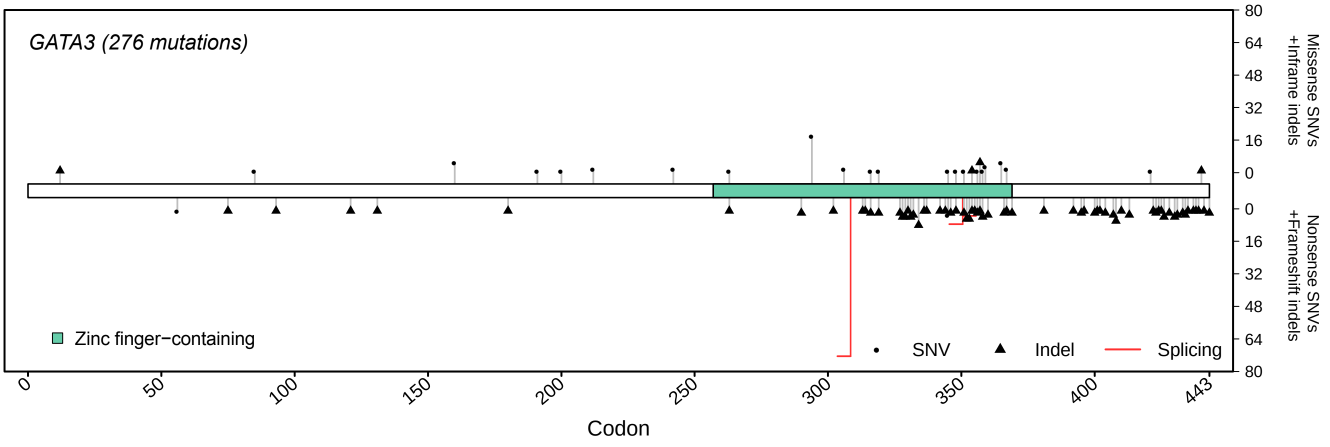

GATA3 (276 mutations)
Zinc finger−containing
SNV
Indel
Splicing
Codon
Missense SNVs +Inframe indels
Nonsense SNVs +Frameshift indels
0 50 100 150 200 250 300 350 400 443
80 64 48 32 16 0 0 16 32 48 64 80

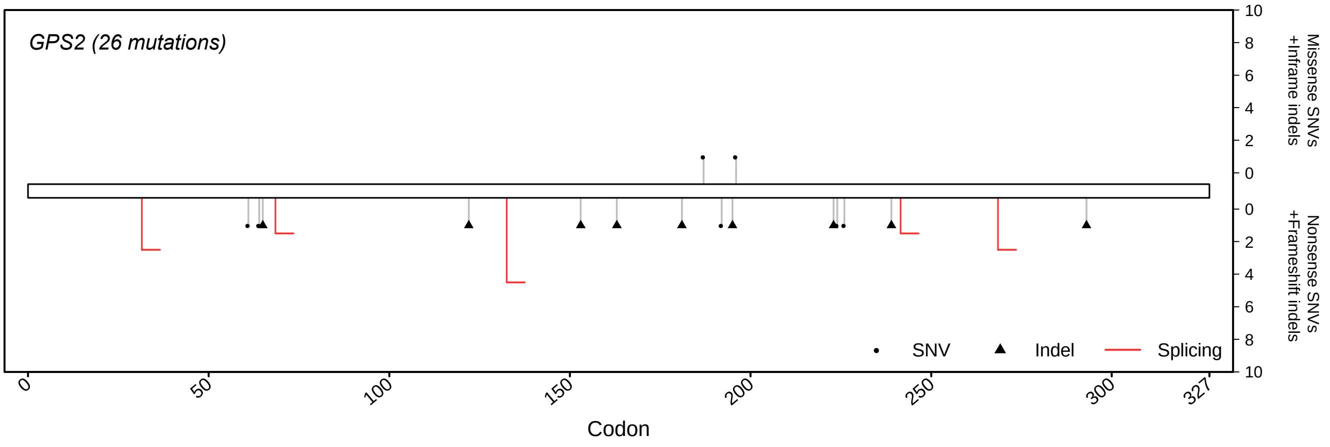

GPS2 (26 mutations)
SNV
Indel
Splicing
Codon
Missense SNVs +Inframe indels
Nonsense SNVs +Frameshift indels
0 50 100 150 200 250 300 327
10 8 6 4 2 0 0 2 4 6 8 10

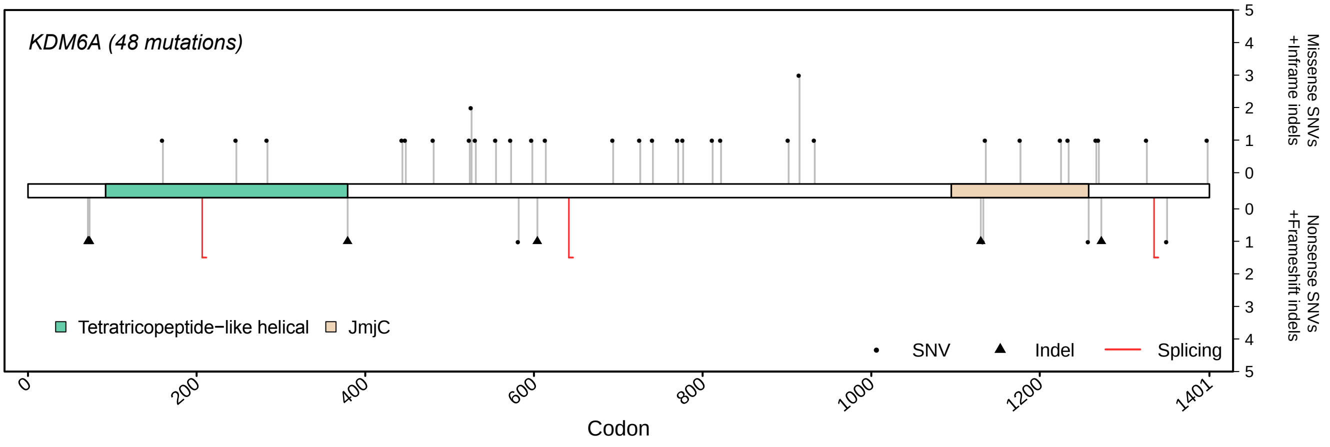

KDM6A (48 mutations)
Tetratricopeptide−like helical
JmjC
SNV
Indel
Splicing
Codon
Missense SNVs +Inframe indels
Nonsense SNVs +Frameshift indels
0 200 400 600 800 1000 1200 1401
5 4 3 2 1 0 0 1 2 3 4 5

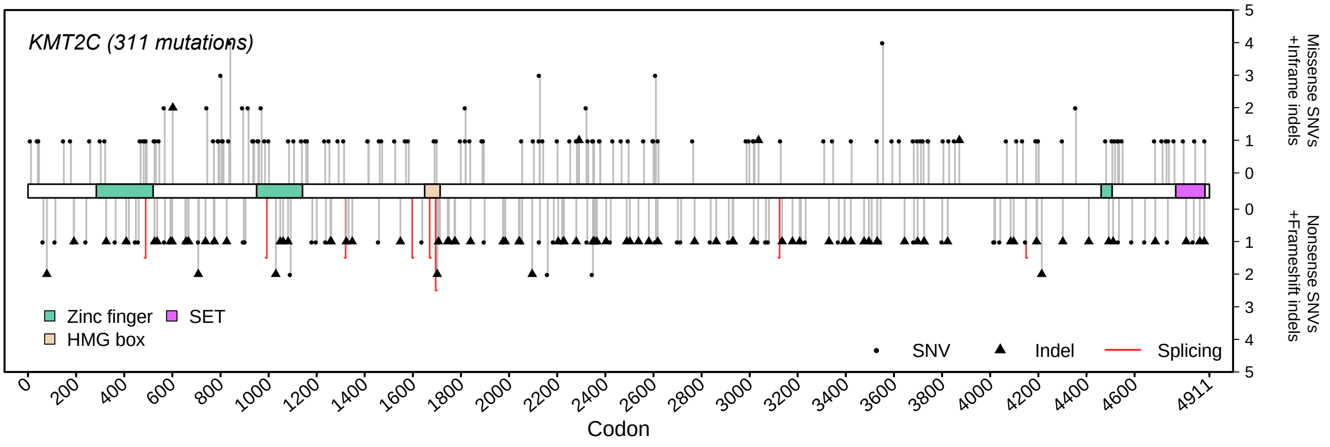

KMT2C (311 mutations)
Zinc finger
SET
HMG box
SNV
Indel
Splicing
Codon
Missense SNVs +Inframe indels
Nonsense SNVs +Frameshift indels
0 200 400 600 800 1000 1200 1400 1600 1800 2000 2200 2400 2600 2800 3000 3200 3400 3600 3800 4000 4200 4400 4600 4911
5 4 3 2 1 0 0 1 2 3 4 5

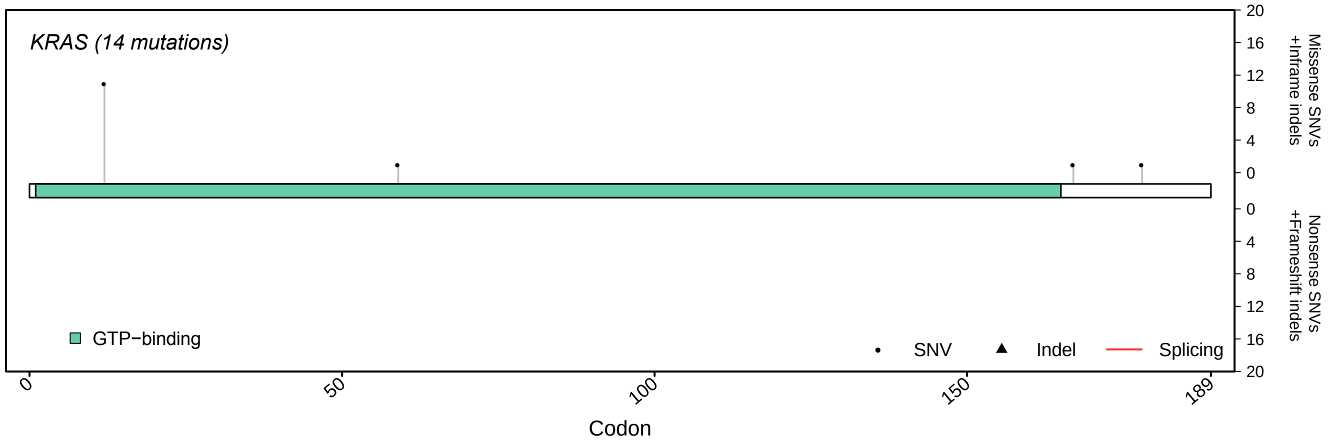

KRAS (14 mutations)
GTP−binding
SNV
Indel
Splicing
Missense SNVs +Inframe indels
Nonsense SNVs +Frameshift indels
Codon

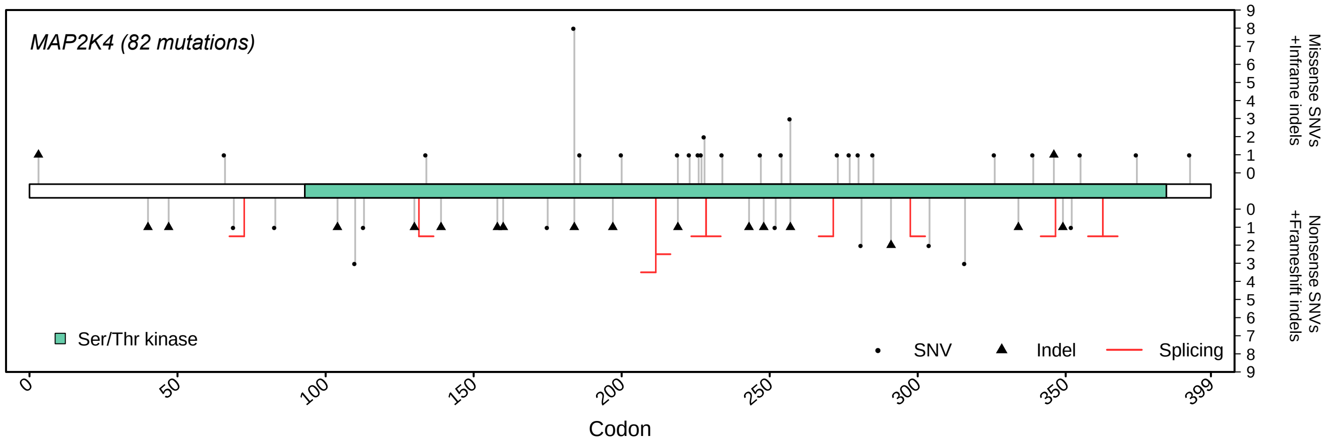

MAP2K4 (82 mutations)
Ser/Thr kinase
SNV
Indel
Splicing
Missense SNVs +Inframe indels
Nonsense SNVs +Frameshift indels
Codon

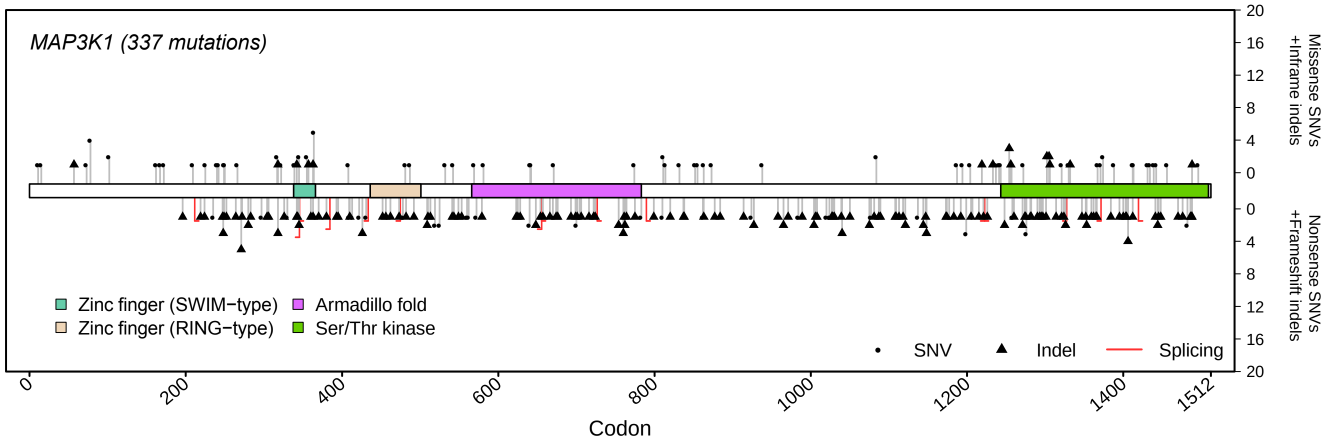

MAP3K1 (337 mutations)
Zinc finger (SWIM−type)
Zinc finger (RING−type)
Armadillo fold
Ser/Thr kinase
SNV
Indel
Splicing
Missense SNVs +Inframe indels
Nonsense SNVs +Frameshift indels
Codon

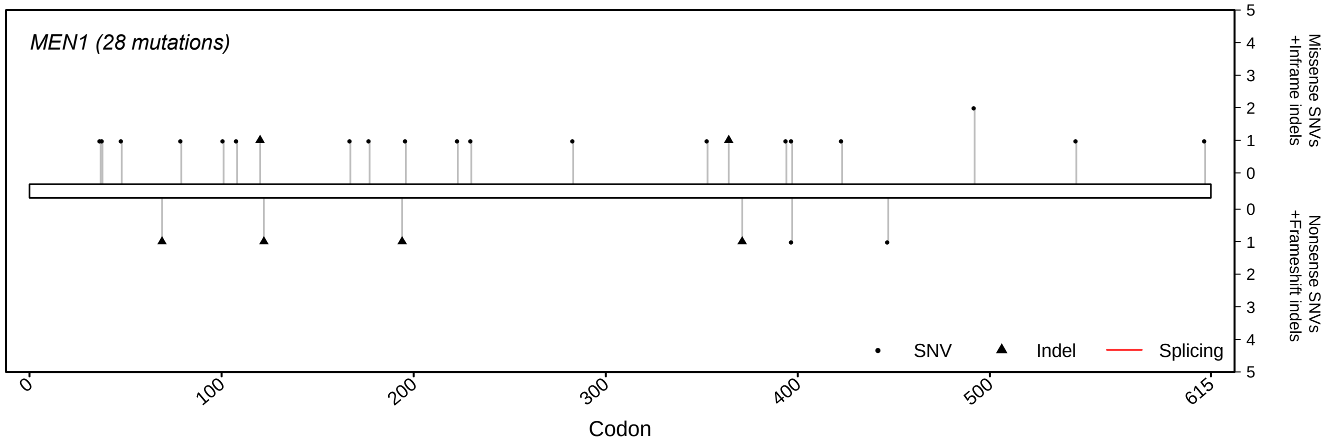

MEN1 (28 mutations)
SNV
Indel
Splicing
Missense SNVs +Inframe indels
Nonsense SNVs +Frameshift indels
Codon

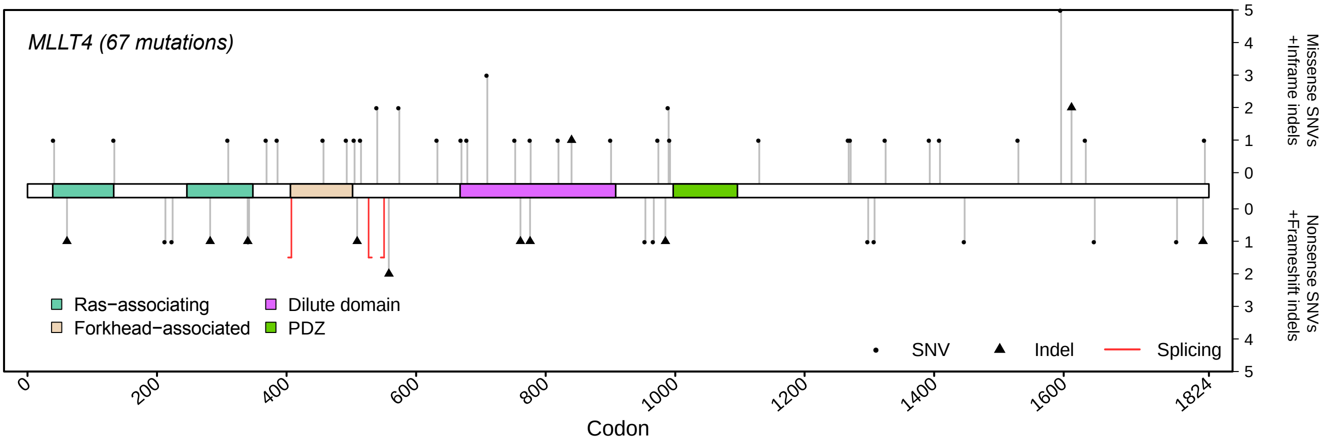

MLLT4 (67 mutations)
Ras−associating
Forkhead−associated
Dilute domain
PDZ
SNV
Indel
Splicing
Missense SNVs +Inframe indels
Nonsense SNVs +Frameshift indels
Codon

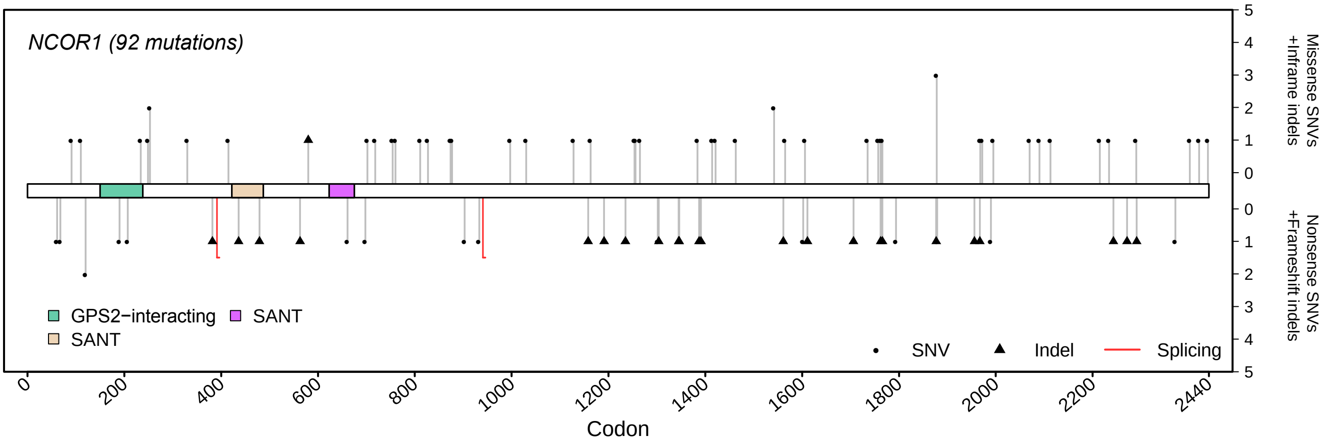

NCOR1 (92 mutations)
GPS2−interacting
SANT
SANT
SNV
Indel
Splicing
Missense SNVs +Inframe indels
Nonsense SNVs +Frameshift indels
Codon

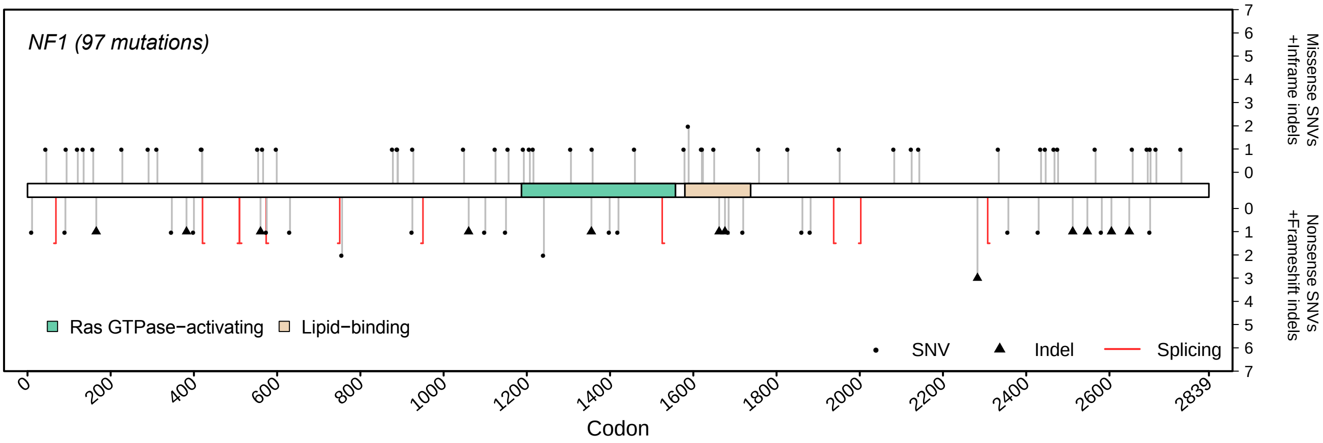

NF1 (97 mutations)
Ras GTPase−activating
Lipid−binding
SNV
Indel
Splicing
Missense SNVs +Inframe indels
Nonsense SNVs +Frameshift indels
Codon

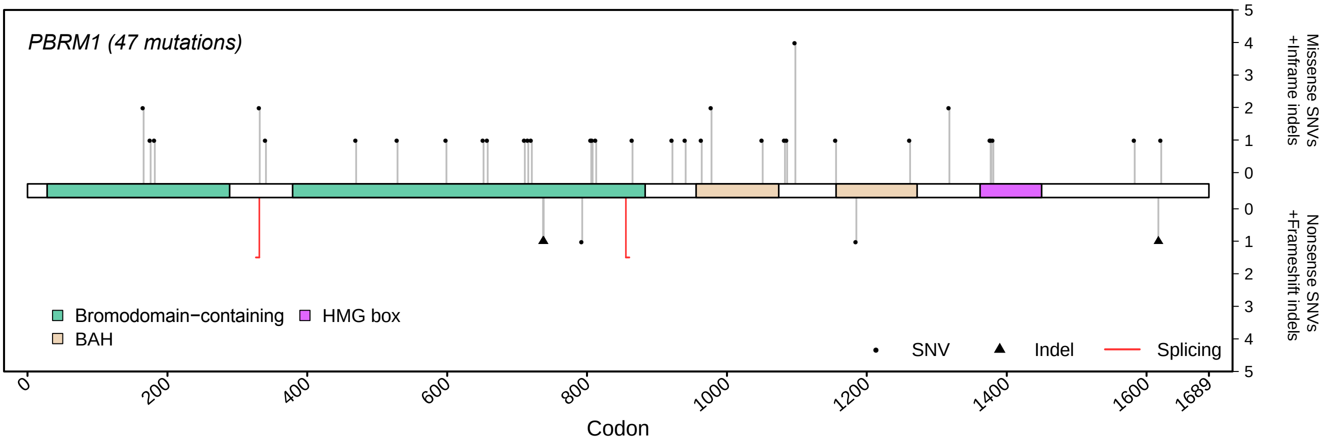

PBRM1 (47 mutations)
Bromodomain−containing
HMG box
BAH
SNV
Indel
Splicing
Missense SNVs +Inframe indels
Nonsense SNVs +Frameshift indels
Codon

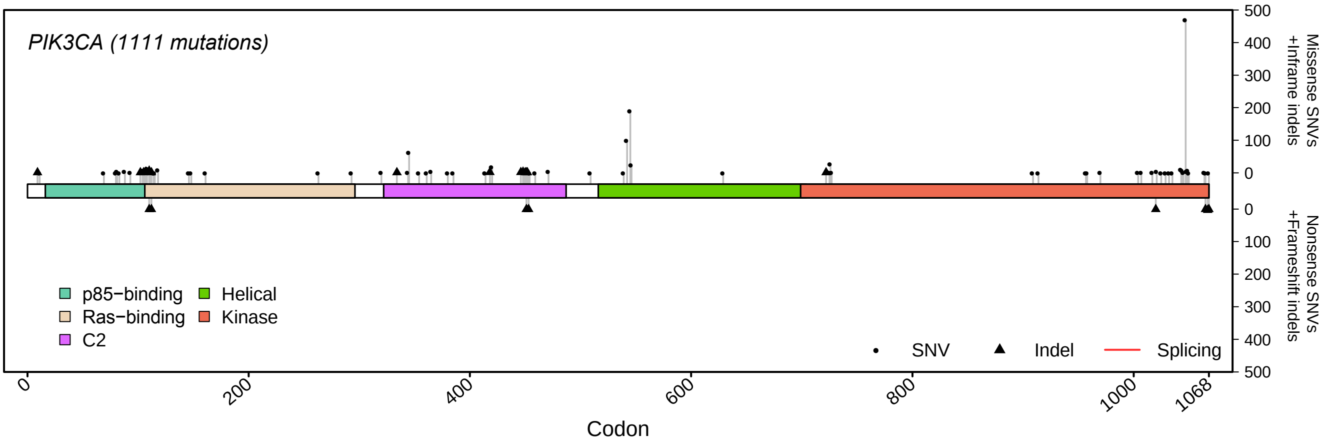
PIK3CA (1111 mutations)
p85−binding
Ras−binding
C2
Helical
Kinase
SNV
Indel
Splicing
Missense SNVs +Inframe indels
Nonsense SNVs +Frameshift indels
500 400 300 200 100 0
0 100 200 300 400 500
0 200 400 600 800 1000 1068
Codon

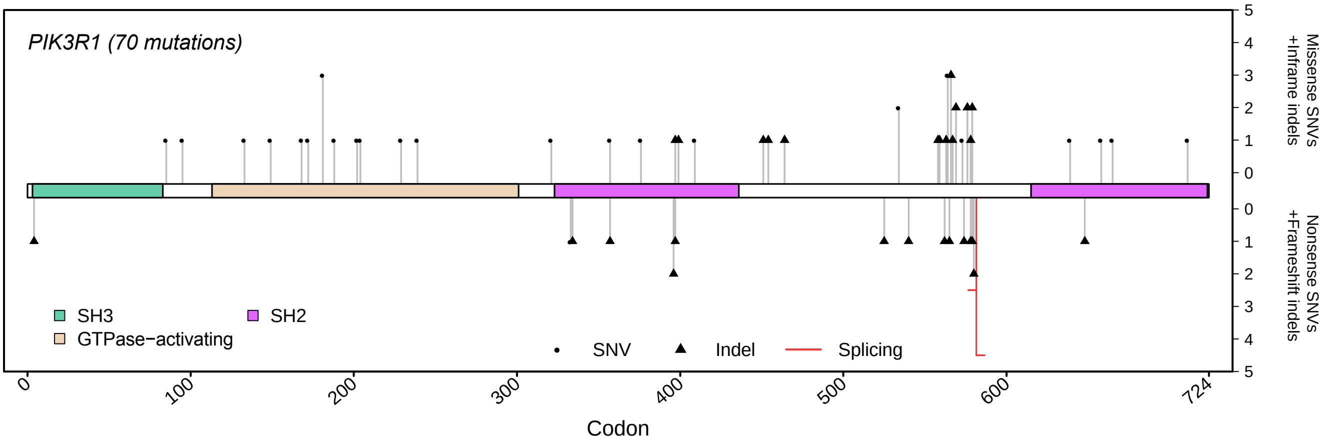
PIK3R1 (70 mutations)
SH3
GTPase−activating
SH2
SNV
Indel
Splicing
Missense SNVs +Inframe indels
Nonsense SNVs +Frameshift indels
5 4 3 2 1 0
0 1 2 3 4 5
0 100 200 300 400 500 600 724
Codon

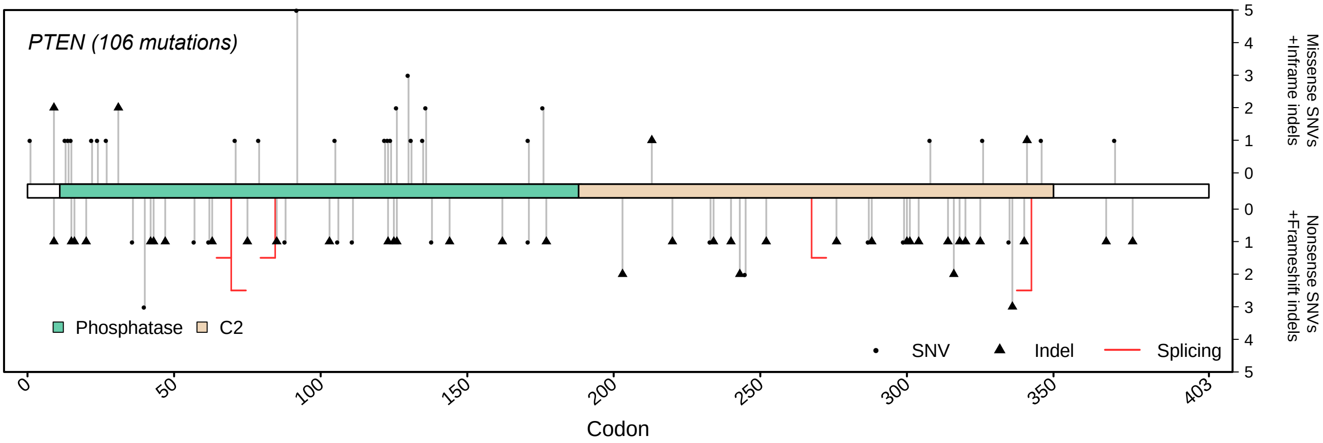
PTEN (106 mutations)
Phosphatase
C2
SNV
Indel
Splicing
Missense SNVs +Inframe indels
Nonsense SNVs +Frameshift indels
5 4 3 2 1 0
0 1 2 3 4 5
0 50 100 150 200 250 300 350 403
Codon

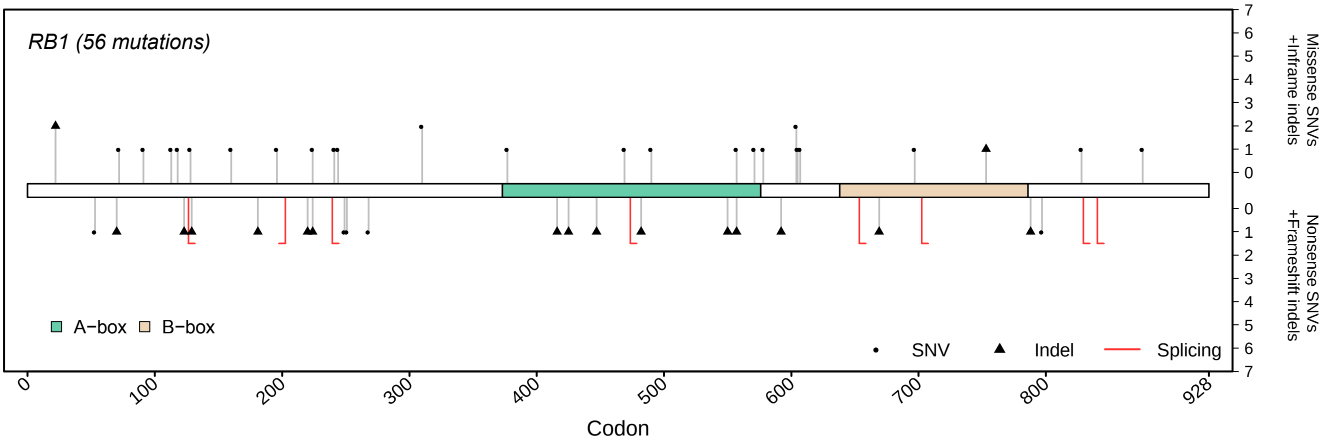
RB1 (56 mutations)
A−box
B−box
SNV
Indel
Splicing
Missense SNVs +Inframe indels
Nonsense SNVs +Frameshift indels
7 6 5 4 3 2 1 0
0 1 2 3 4 5 6 7
0 100 200 300 400 500 600 700 800 928
Codon

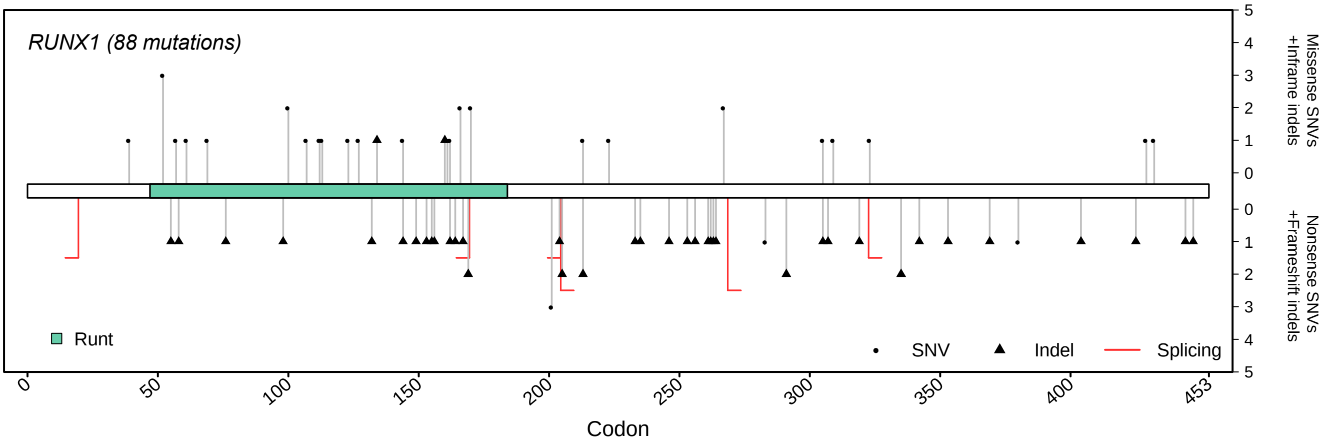
RUNX1 (88 mutations)
Runt
SNV
Indel
Splicing
Missense SNVs +Inframe indels
Nonsense SNVs +Frameshift indels
0 50 100 150 200 250 300 350 400 453
Codon

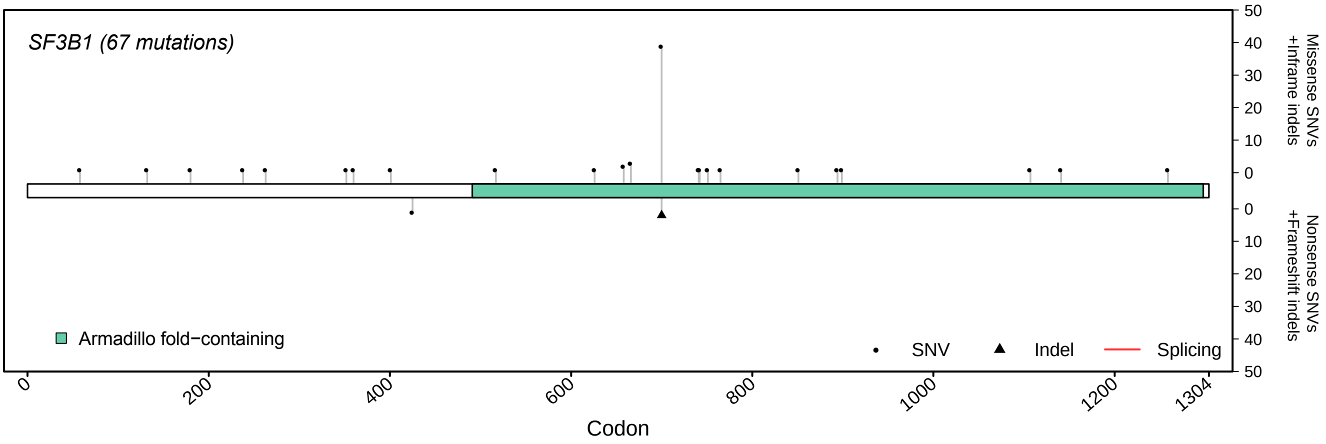
SF3B1 (67 mutations)
Armadillo fold−containing
SNV
Indel
Splicing
Missense SNVs +Inframe indels
Nonsense SNVs +Frameshift indels
0 200 400 600 800 1000 1200 1304
Codon

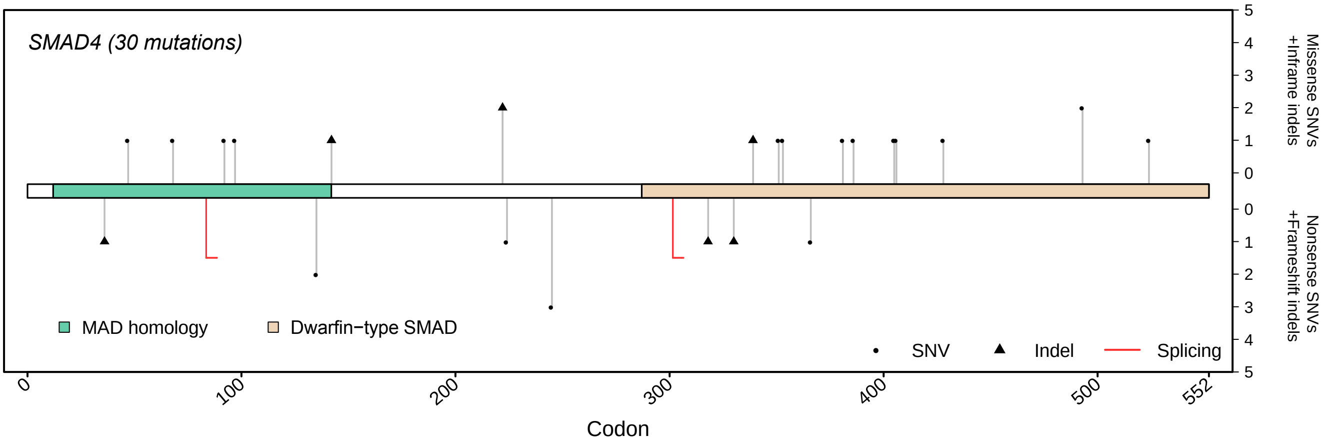
SMAD4 (30 mutations)
MAD homology
Dwarfin−type SMAD
SNV
Indel
Splicing
Missense SNVs +Inframe indels
Nonsense SNVs +Frameshift indels
0 100 200 300 400 500 552
Codon

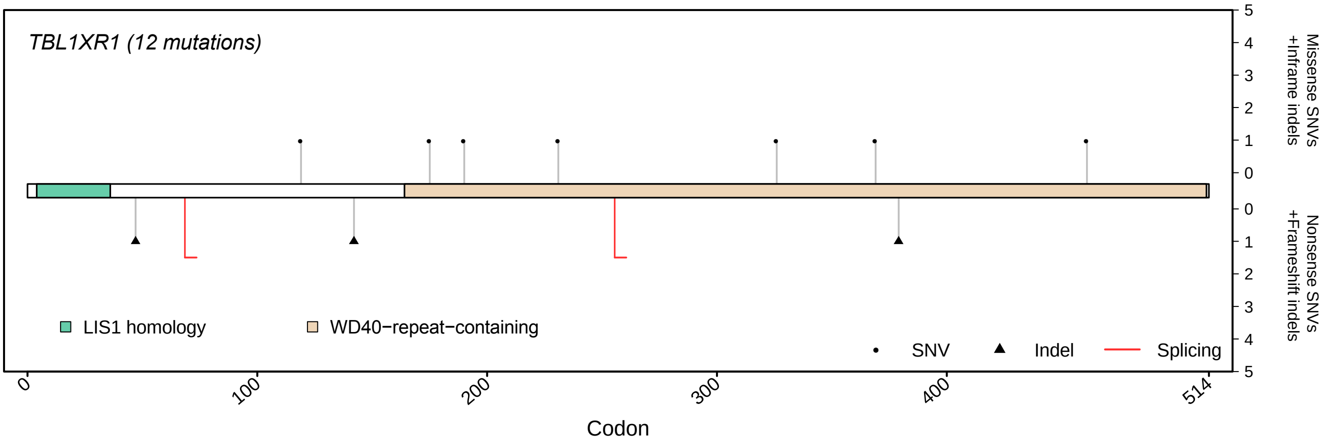
TBL1XR1 (12 mutations)
LIS1 homology
WD40−repeat−containing
SNV
Indel
Splicing
Missense SNVs +Inframe indels
Nonsense SNVs +Frameshift indels
0 100 200 300 400 514
Codon

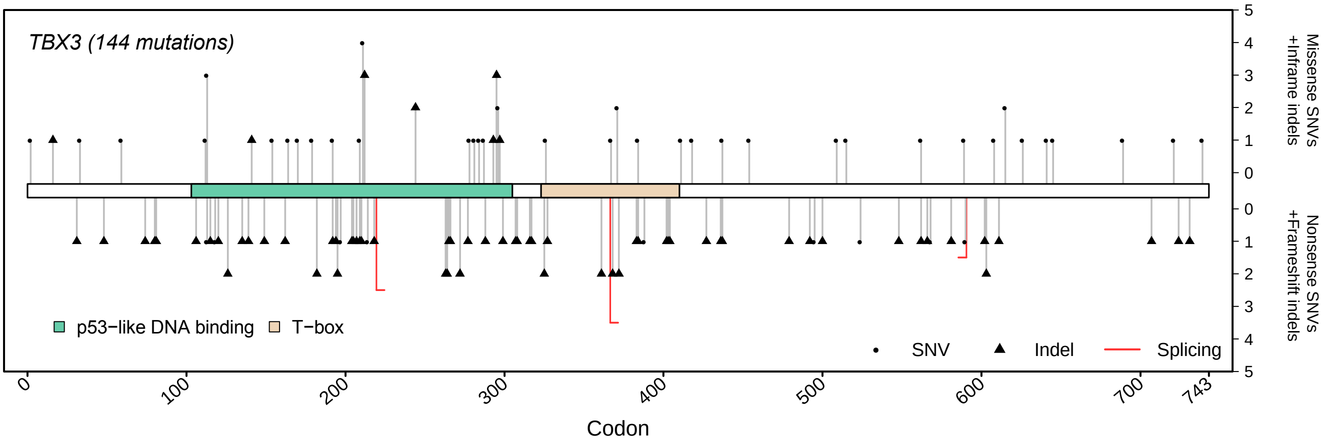
TBX3 (144 mutations)
p53−like DNA binding
T−box
SNV
Indel
Splicing
Missense SNVs +Inframe indels
Nonsense SNVs +Frameshift indels
0
100
200
300
400
500
600
700
743
Codon

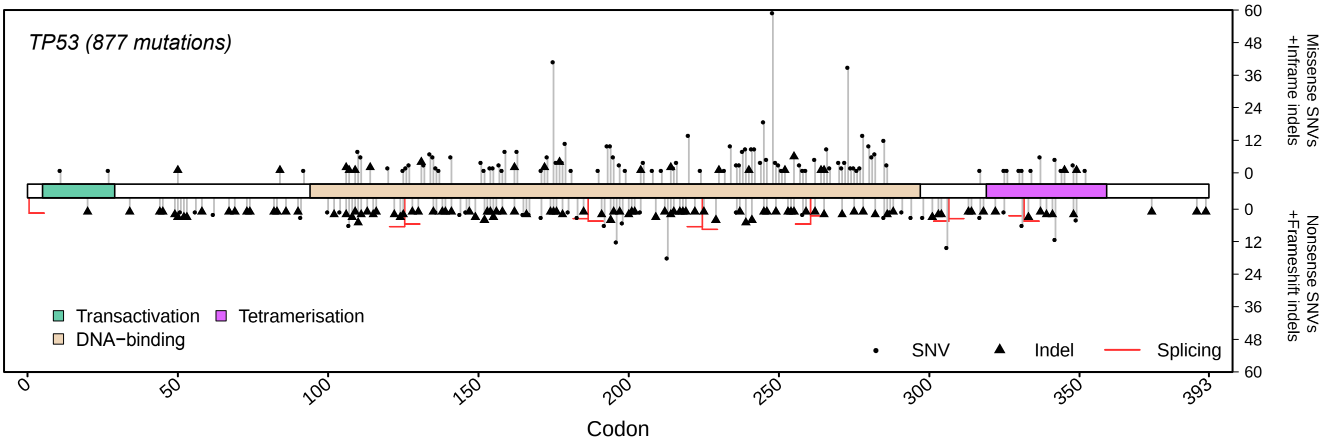
TP53 (877 mutations)
Transactivation
Tetramerisation
DNA−binding
SNV
Indel
Splicing
Missense SNVs +Inframe indels
Nonsense SNVs +Frameshift indels
0
50
100
150
200
250
300
350
393
Codon

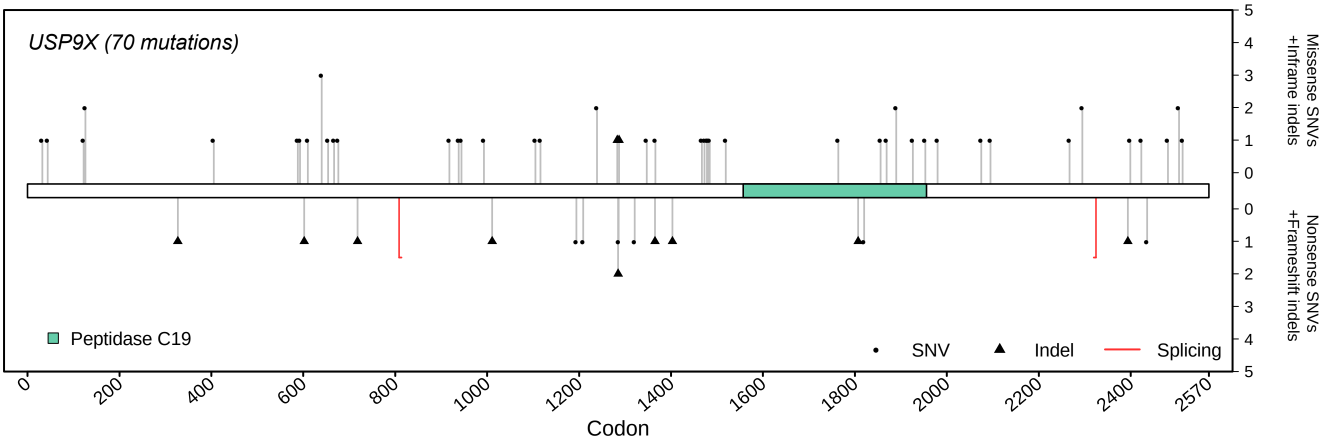
USP9X (70 mutations)
Peptidase C19
SNV
Indel
Splicing
Missense SNVs +Inframe indels
Nonsense SNVs +Frameshift indels
0
200
400
600
800
1000
1200
1400
1600
1800
2000
2200
2400
2570
Codon

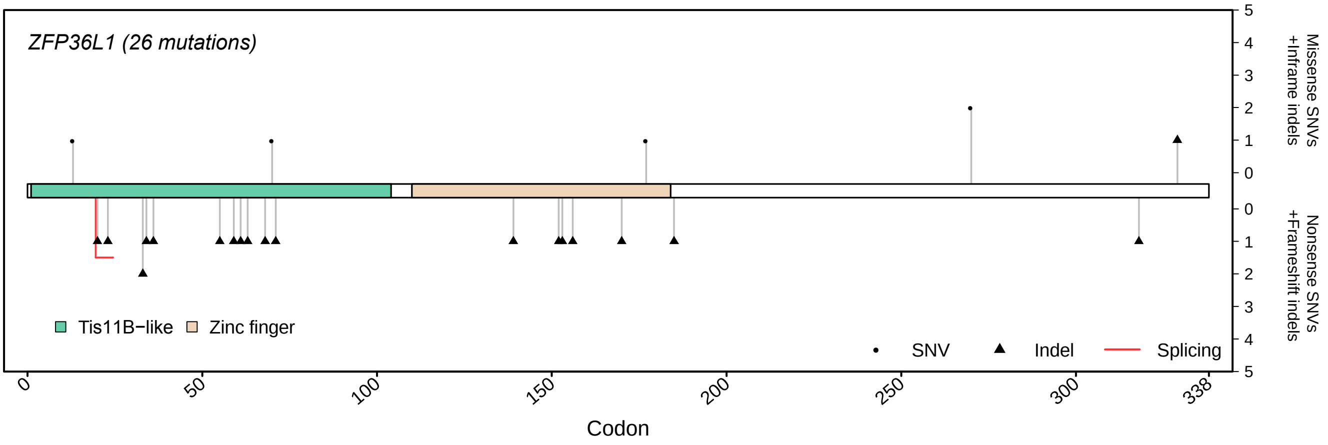
ZFP36L1 (26 mutations)
Tis11B−like
Zinc finger
SNV
Indel
Splicing
Missense SNVs +Inframe indels
Nonsense SNVs +Frameshift indels
0
50
100
150
200
250
300
338
Codon

Supplementary Fig. 5

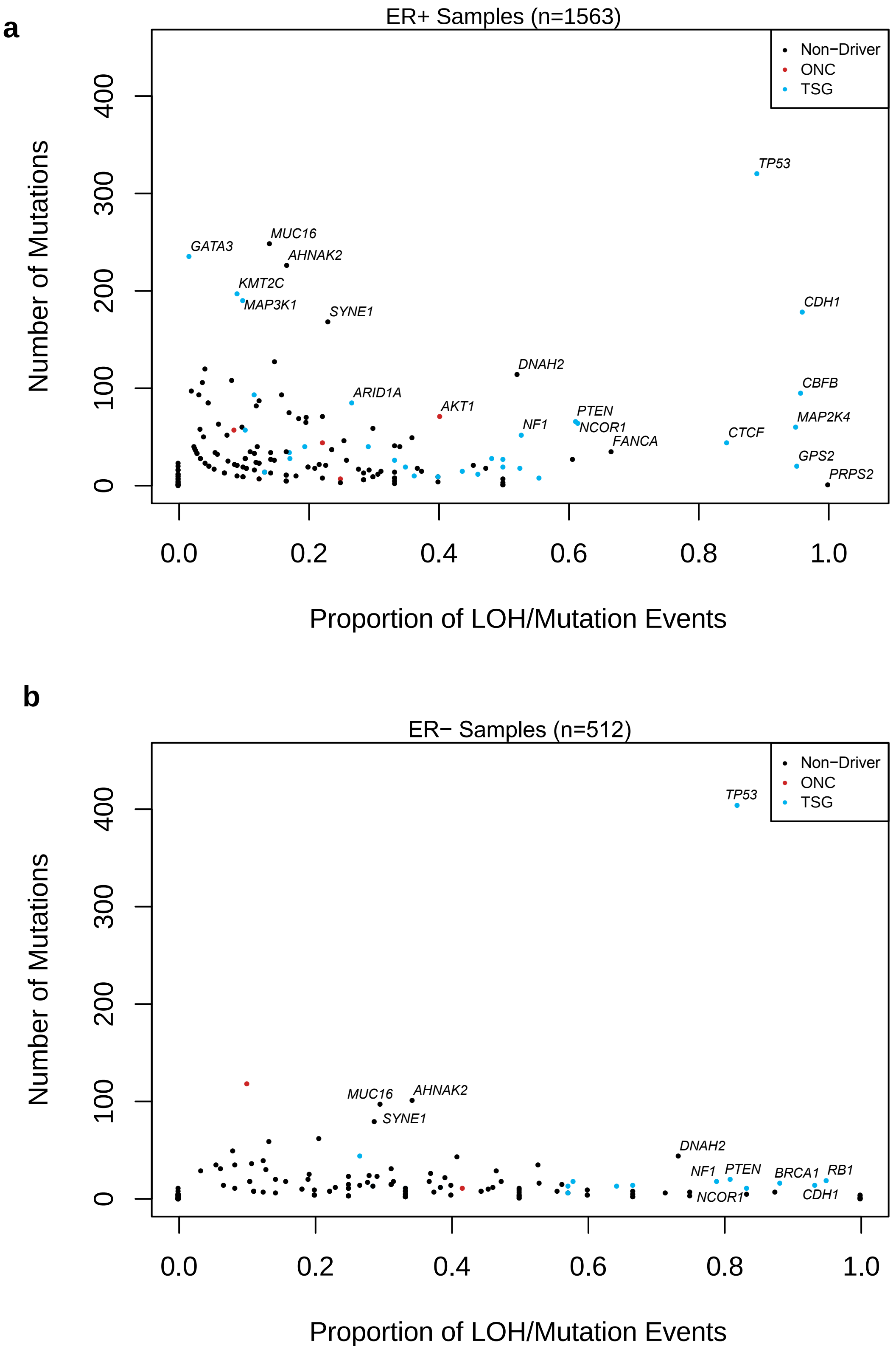

Supplementary Figure 5 – Proportions of mutations accompanied by LOH.
The numbers of mutations in each of the 173 genes and the proportions of these mutations accompanied by loss of heterozygosity (LOH) are shown for (a) ER+ and (b) ER- tumours. LOH was defined as any CNA in which one allele was entirely deleted **(*Methods*)**. Mut-driver genes from ***Figure 1a*** are indicated in red or blue respectively, and all other genes are represented by black dots. Genes with high numbers of mutations or displaying a high proportion of mutation/LOH events are named.

Supplementary Fig. 6

a

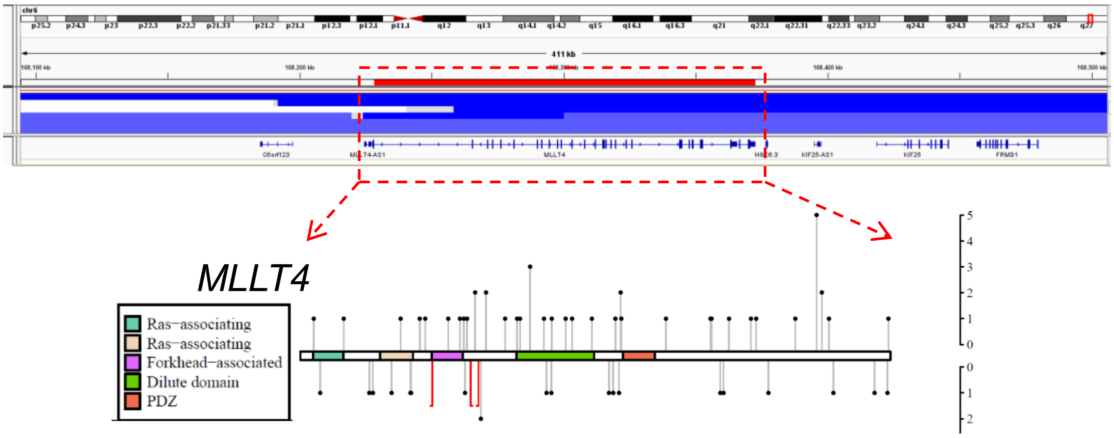

b

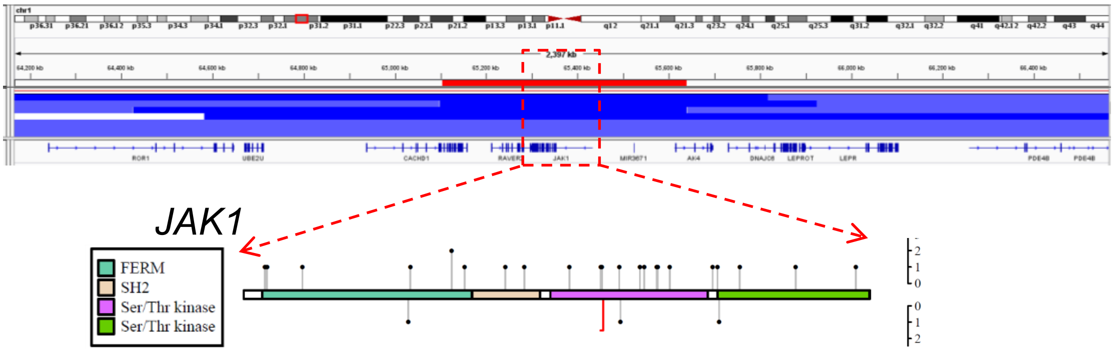

Supplementary Figure 6 – Homozygous deletions and truncating mutations identify tumour suppressor genes.
Tracks from the Integrative Genomics Viewer (IGV) showing regions of minimal deletion region (top panel) and the corresponding mutation spectra (bottom panel) for the targeted genes: a) *MLLT4* b) *JAK1*. Observing homozygous deletions and inactivating mutations in a gene provides evidence for a tumour suppressor role in breast cancer.

Supplementary Fig. 7

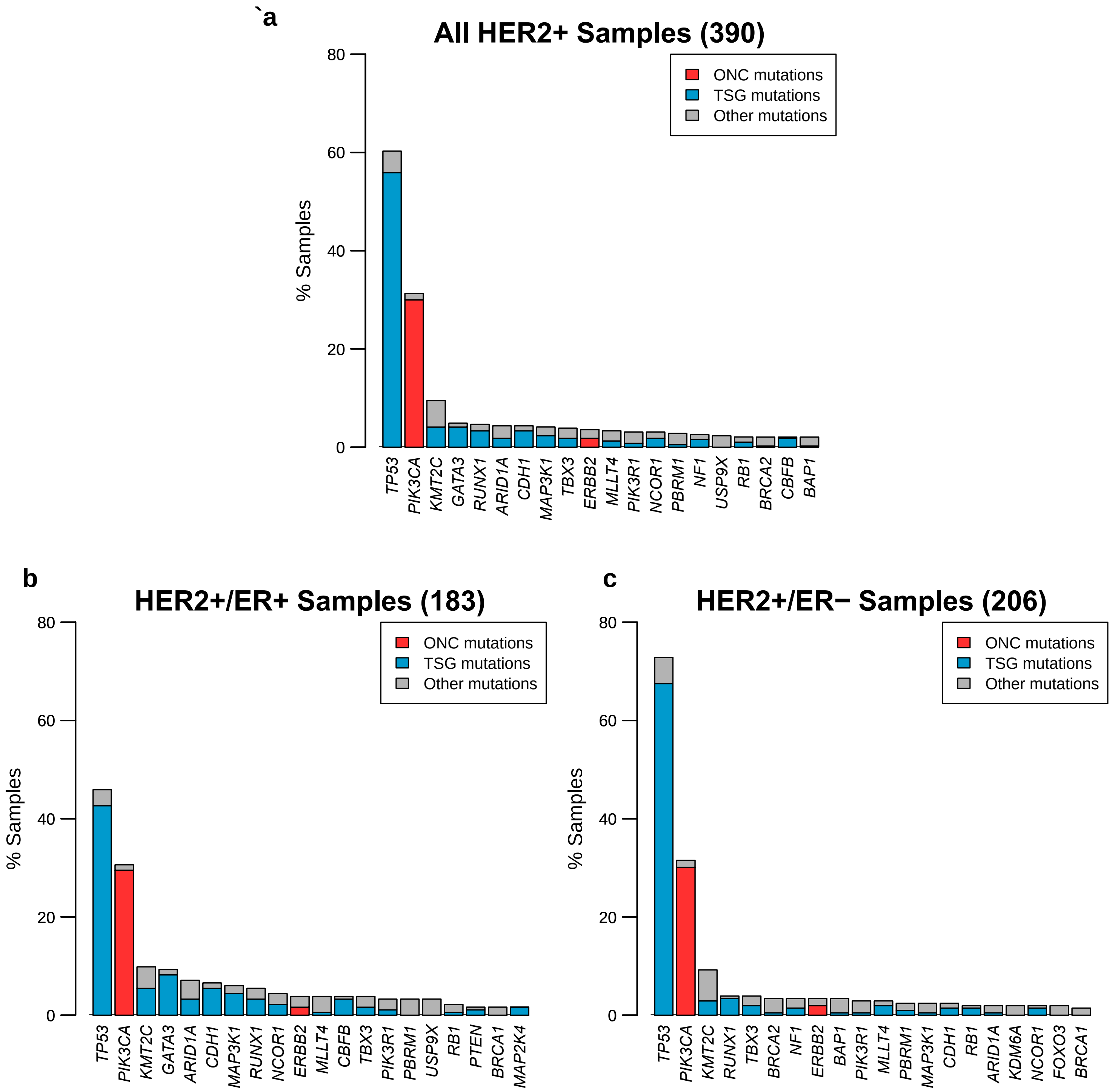

Supplementary Figure 7 – Mutation distributions in HER2+ tumours.
Bars indicate percentages of samples with mutations in the 20 most frequently mutated Mut-driver genes in the specified cohorts. The coloured part of the bars represents functional mutations. These were defined as recurrent mutations that contributed towards an oncogene's ONC score (red), or inactivating mutations that contributed towards a tumour suppressor gene's TSG score (blue; both recurrent and inactivating mutations were used for *TP53*). The numbers of samples in each cohort is indicated in brackets.

a) Mutations for all HER2+ tumours.
b) Mutations for the HER2+/ER+ subset.
c) Mutations for the HER2+/ER- subset.

Supplementary Fig. 8

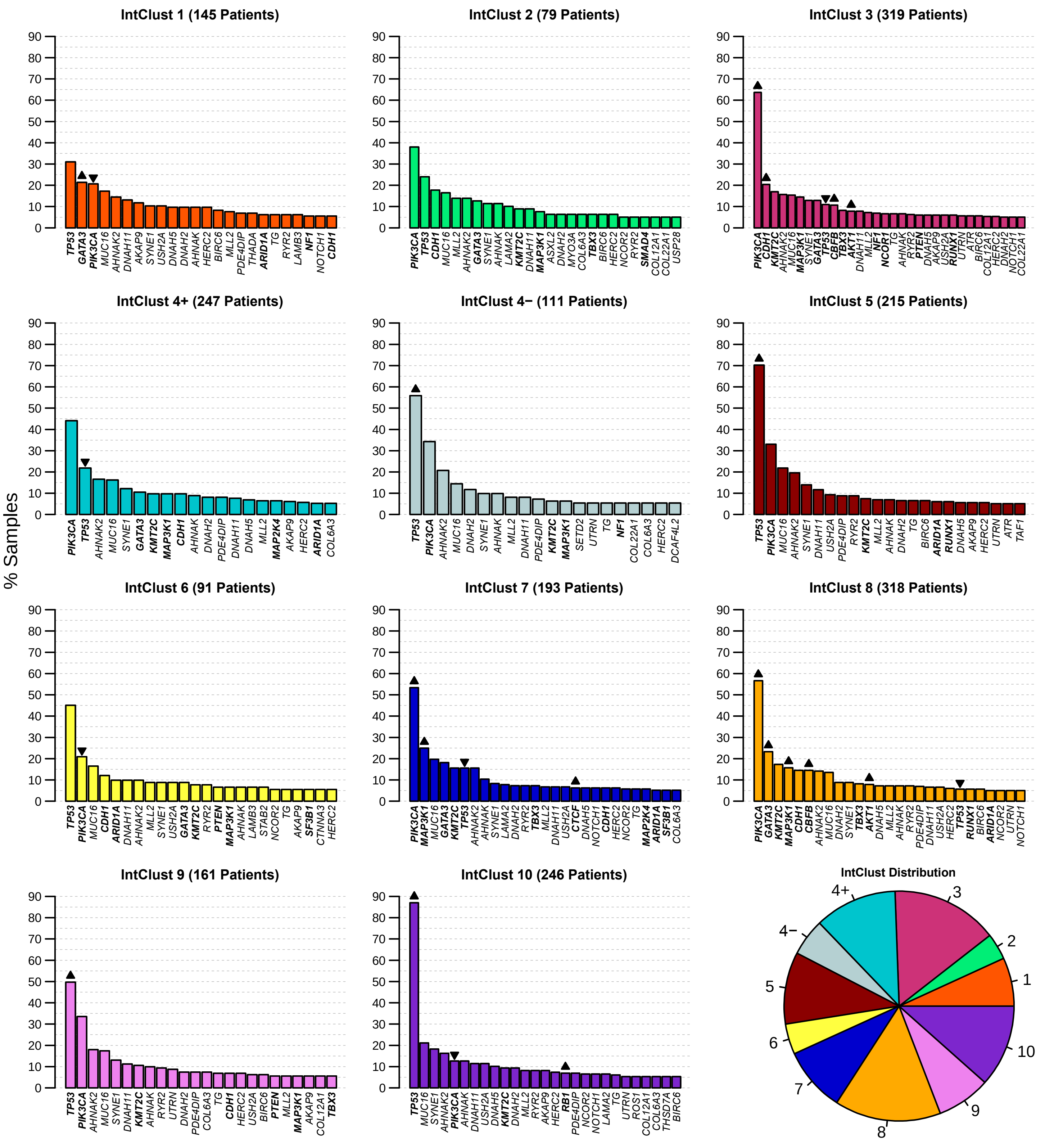

Supplementary Figure 8– Mutation frequencies across the IntClusts.
Bars represent percentages of samples with mutations in genes mutated in at least 5% of the tumours within each IntClust. All coding mutations are shown. ▲▼ represent mutations over- or under-represented in tumours belonging to a particular IntClust relative to all other tumours (Fisher's exact test; FDR=0.05). IntClust4 stratified by ER status: IntClust4+, ER+; and IntClust4-, ER- tumours. Gene names in bold were identified as Mut-driver genes. The pie chart depicts the distribution of tumours across the IntClusts.

Supplementary Fig. 9

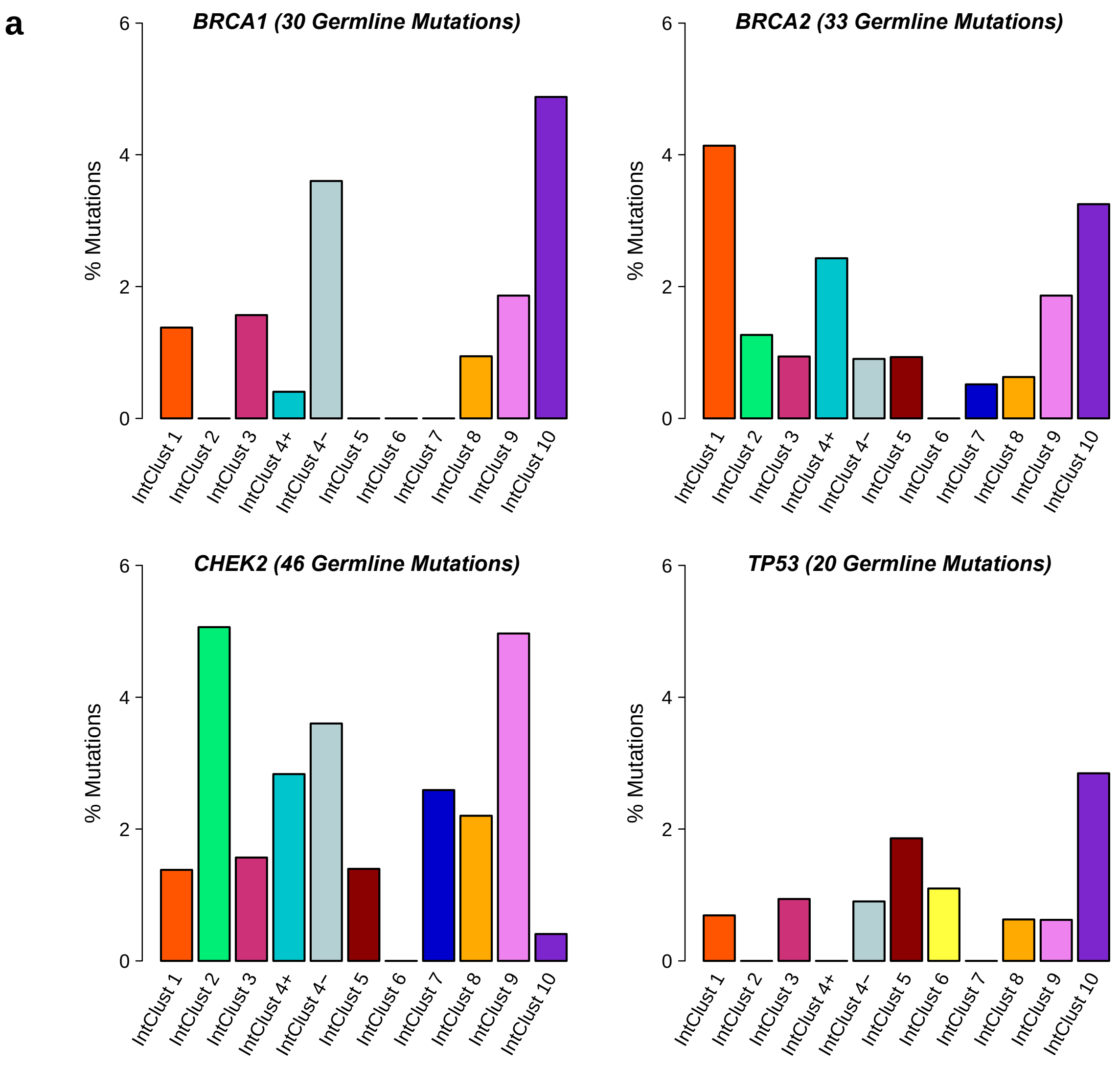

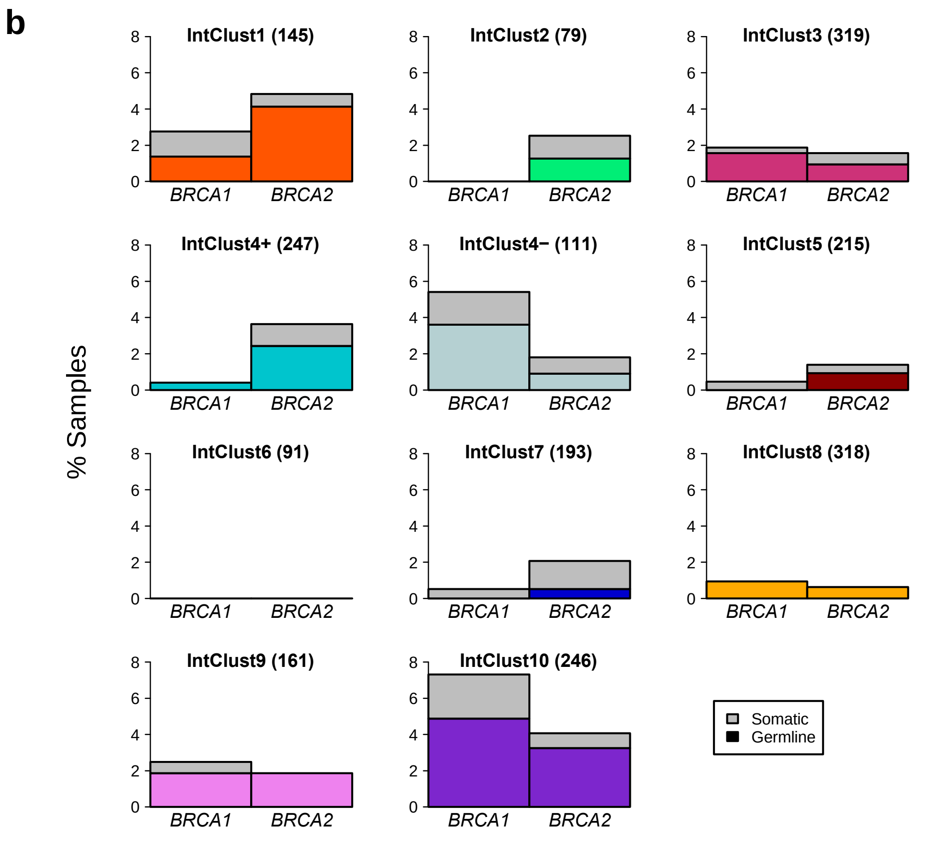

Supplementary Figure 9 – Pathogenic germline mutations in the IntClusts.

a) Distributions of pathogenic germline mutations for *BRCA1*, *BRCA2*, *CHEK2*, *TP53*.
b) Bars represent pathogenic germline (coloured) and somatic (gray) mutation frequencies for *BRCA1* and *BRCA2* across the IntClusts.

Supplementary Fig. 10

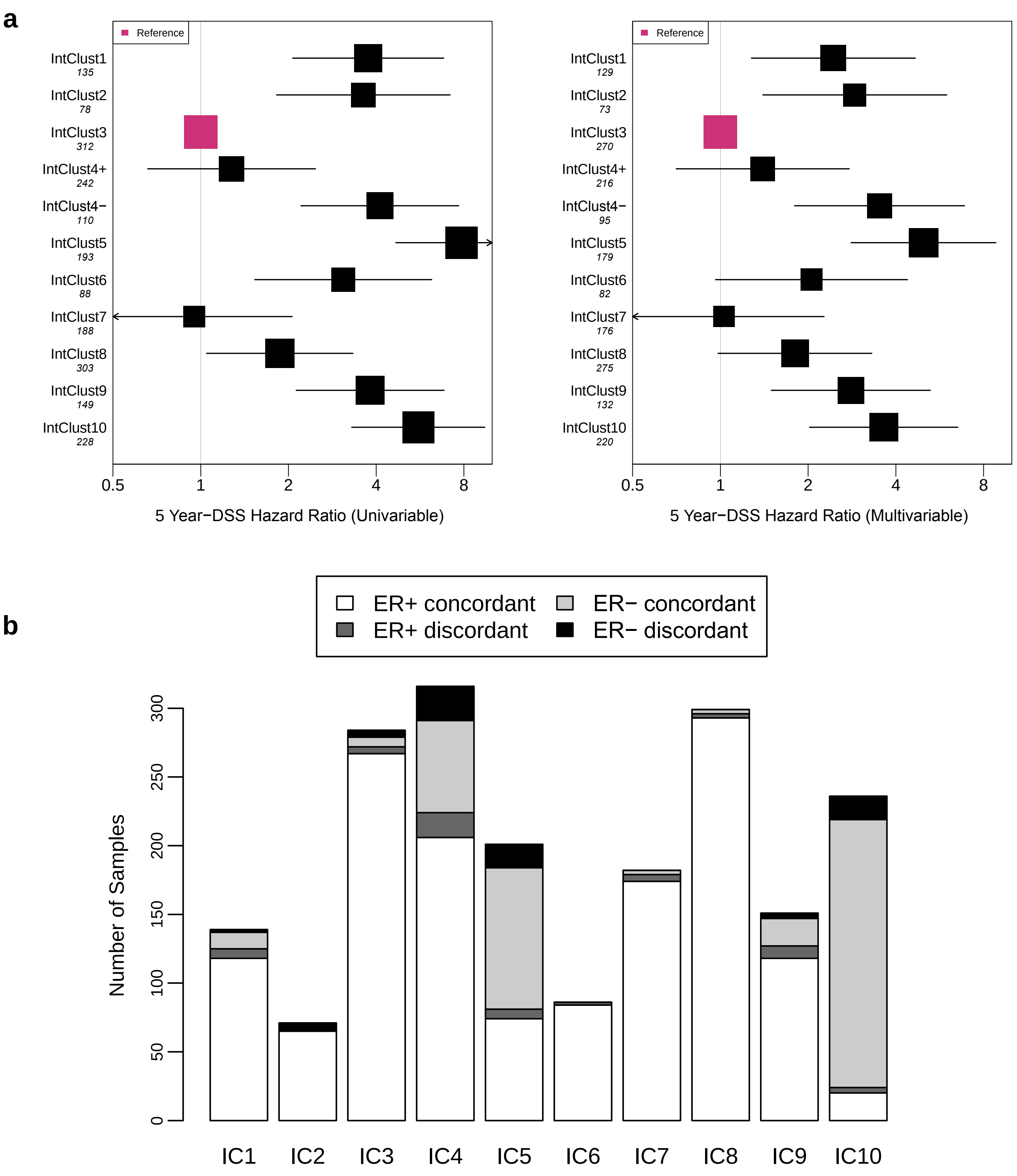

Supplementary Figure 10– Characteristics of the Integrative Clusters.

a) Hazard ratio plots. *Left*: A univariable Cox model was constructed to explore the effects on disease-specific survival (DSS) of IntClust membership. Lines depict 95% confidence intervals. IntClust3 (■) was used as reference. The size of the box corresponds to the inverse of the width of the confidence interval. *Right*: Hazard ratios for 5-year disease-specific survival were obtained using a multivariable Cox model that accounted for size, grade, age and lymph nodes status. Variables were coded as described in ***Methods***.

b) Fractions of tumours within the IntClusts that are ER+ and ER-. Fraction of samples for which *ESR1* gene expression corroborated the immunohistochemistry-based (IHC) ER status is indicated (concordant=expression matched IHC; discordant=expression different from IHC). In discordant cases, the IHC score was only over-ruled if the probability of belonging to the other category was high by gene expression. The criteria used for the final classification of ER status are described more fully in the ***Methods*** section.

Supplementary Fig. 11

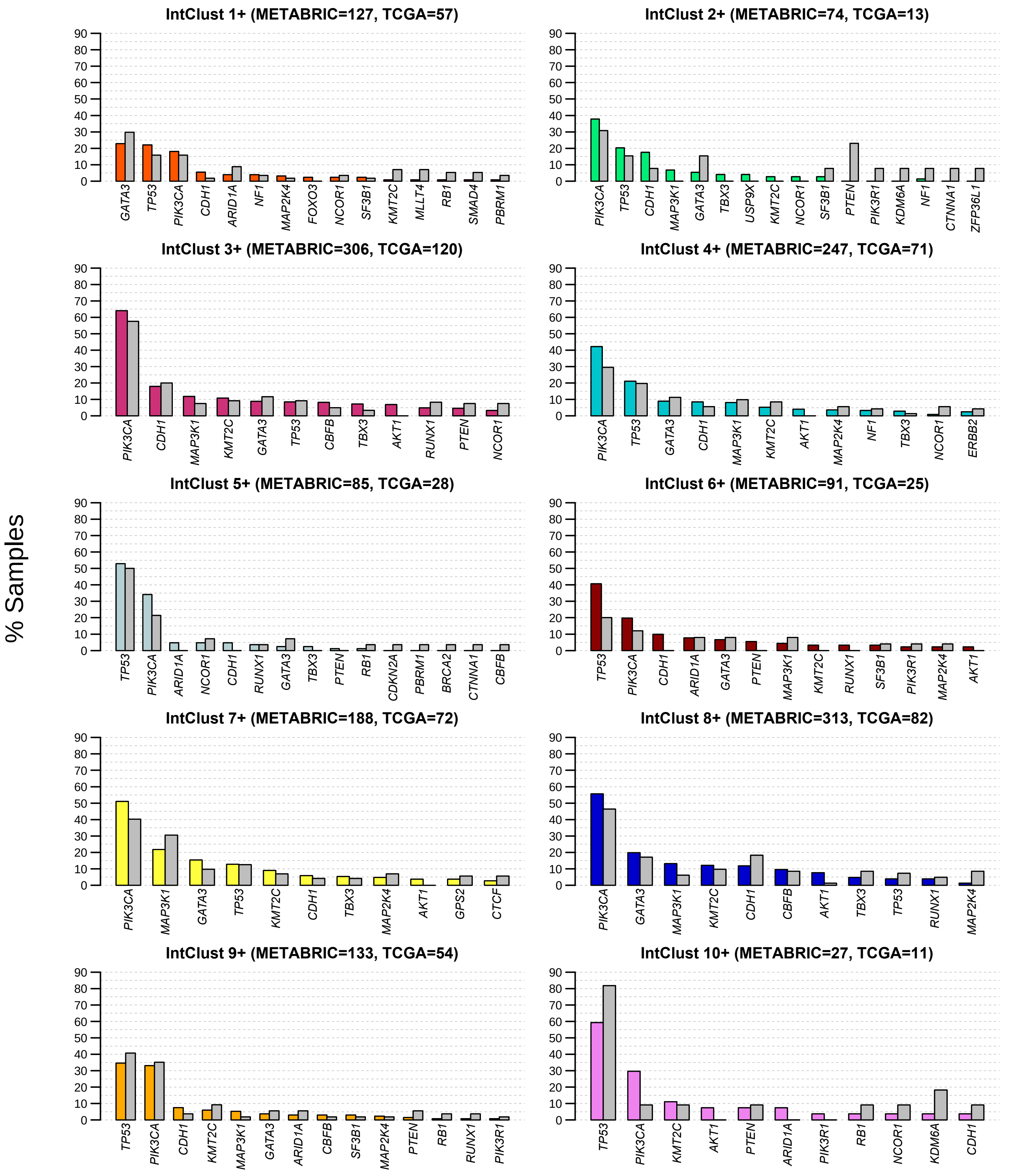

Supplementary Figure 11 – Mutations in Mut-driver genes in ER+ tumours stratified by IntClust.

Bars represent mutation frequencies in samples from METABRIC (coloured bars) and TCGA (gray bars) for the top 10 most frequently mutated genes across both datasets. Only functional mutations are shown for the METABRIC data, whereas all coding mutations are shown for TCGA.

Supplementary Fig. 12

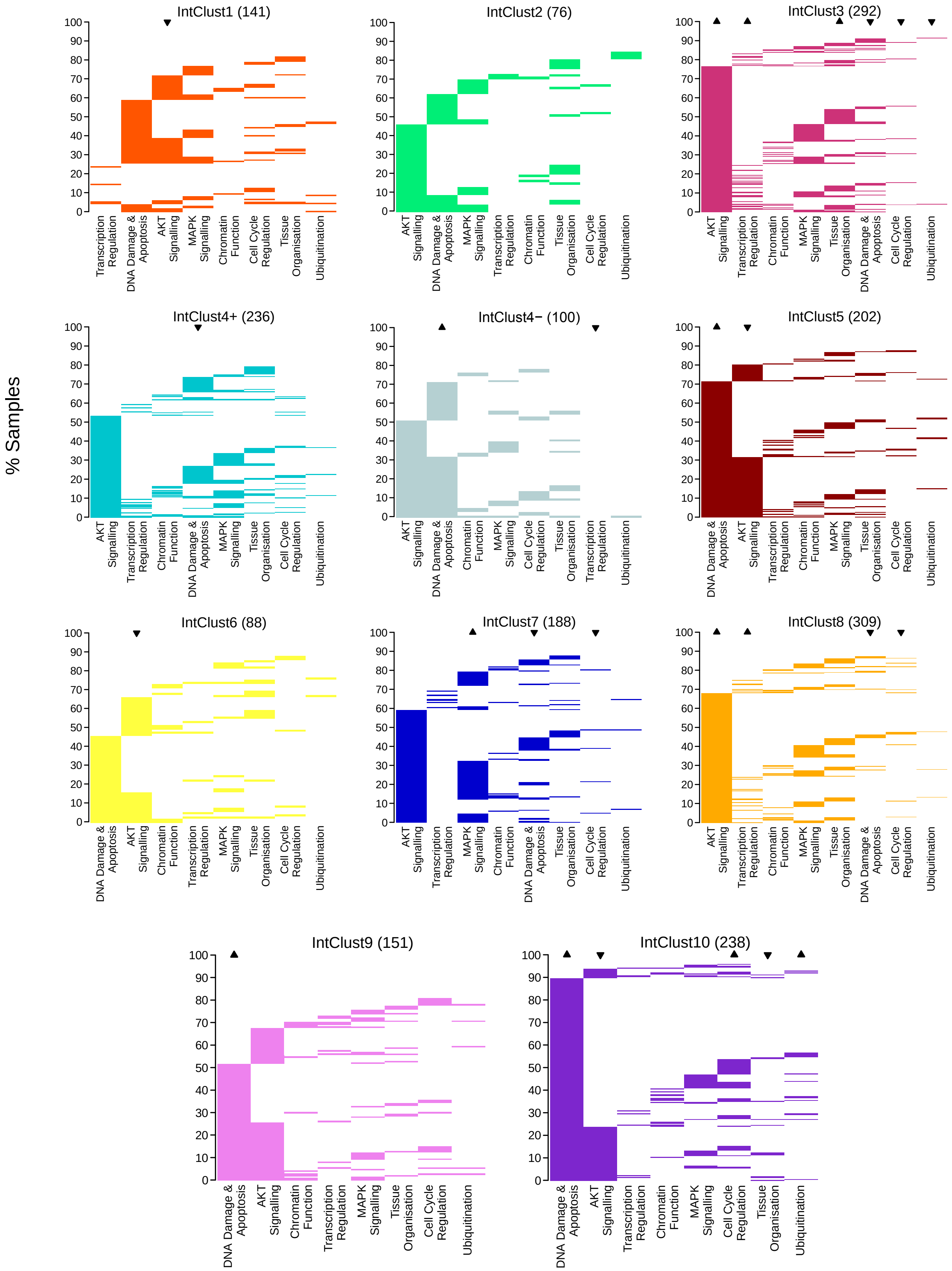

Supplementary Figure 12 – Aggregate distributions by pathways of Mut-driver gene mutations and CNAs.
All functional mutations, high-level amplifications (5+ copies) and homozygous deletions in the 40 Mut-driver genes were used, and a pathway was considered altered if at least one component gene was altered. The samples belonging to each IntClust are plotted along the y-axis. The ▲▼ represent pathways that are more or less frequently altered in a given IntClust relative to all other samples at FDR=0.05.

Supplementary Fig. 13

	IntClust1	IntClust2	IntClust3	IntClust4+	IntClust4–	IntClust5	IntClust6	IntClust7	IntClust8	IntClust9	IntClust10
PIK3CA	0.9	0.91	0.84	0.89	0.85	0.93	0.95	0.87	0.86	0.94	0.97
AKT1	1	1	1	1	1	1	1	0.83	0.92	1	1
PTEN	1	1	0.84	0.7	1	1	1	0.57	0.86	0.78	0.82
PIK3R1	1	1	0.88	1	0.5	1	0.75	1	0.91	0.67	0.89
FOXO3	1	0.67	1	0.8	1	1	1	1	0.8	1	1
RB1	1	NA	1	0.75	1	0.75	1	1	1	1	1
CDKN1B	1	NA	1	1	NA	1	NA	1	0.75	0.33	1
CDKN2A	NA	1	1	1	1	1	NA	1	1	0.5	NA
KMT2C	1	0.71	0.77	0.78	0.88	0.94	1	0.85	0.89	0.81	0.84
ARID1A	0.56	0.57	0.85	0.71	1	0.92	0.78	0.8	0.93	0.75	0.62
NCOR1	1	1	0.78	0.8	1	0.78	0.67	0.8	0.88	1	1
CTCF	1	1	0.7	0.83	NA	NA	1	1	0.75	1	1
KDM6A	1	NA	0.88	0.6	1	1	1	0.67	0.5	1	1
PBRM1	1	1	1	NA	1	1	1	1	1	0.75	0.71
TBL1XR1	1	NA	1	1	NA	NA	NA	0.5	1	1	NA
TP53	0.95	0.94	0.89	0.9	0.92	0.92	0.88	0.82	0.94	0.96	0.96
BRCA2	1	1	0.8	1	1	1	1	1	1	1	0.8
BRCA1	1	NA	1	1	1	1	1	0.67	1	1	0.9
CHEK2	1	NA	1	1	1	NA	1	1	1	1	NA
MAP3K1	1	1	0.77	1	1	1	0.75	0.94	0.89	0.9	1
NF1	1	0.67	0.86	1	0.83	0.8	1	0.83	1	1	0.71
MAP2K4	1	1	1	0.81	1	1	1	0.67	0.86	1	1
GPS2	NA	NA	1	1	NA	NA	1	1	0.75	NA	NA
KRAS	NA	NA	1	1	0	NA	NA	0	1	1	0.33
CDH1	1	1	0.84	0.81	1	0.9	0.9	0.85	0.98	1	NA
MLLT4	1	0.5	0.88	0.67	1	1	0.67	1	1	1	0.83
CTNNA1	1	NA	1	1	1	NA	NA	1	1	0.5	NA
GATA3	1	1	0.86	0.92	1	0.75	1	0.94	0.9	0.57	1
TBX3	1	1	0.93	0.91	1	1	1	1	0.85	0.9	1
CBFB	NA	NA	0.87	1	NA	1	NA	0.75	0.93	1	1
RUNX1	0.33	1	0.89	1	NA	0.92	0.67	1	0.9	1	0.88
ZFP36L1	1	NA	1	1	NA	1	1	1	0.5	0.67	0.8
MEN1	1	1	1	0.75	NA	1	1	0.8	1	1	1
FOXP1	NA	1	1	1	NA	1	NA	1	0.67	0	NA
USP9X	0.75	1	1	1	1	0.83	NA	0.83	1	0.83	1
BAP1	NA	1	1	1	1	0.75	1	1	0.5	1	1
ERBB2	0.75	1	0.91	1	1	1	1	1	0.85	1	1
SF3B1	0.67	1	0.86	0.67	NA	1	1	0.9	0.89	0.75	1
SMAD4	0.5	0.25	0.75	1	1	0.33	NA	0.75	0.67	NA	NA
AGTR2	1	0	1	NA	NA	1	0	1	NA	NA	1

Supplementary Figure 13 – Cancer cell fractions of Mut-driver gene mutations across the IntClusts.
The proportions of clonal mutations in Mut-driver genes are shown. We computed the cancer cell fractions (CCFs; ***Methods***) for each mutation and classified mutations as being ‘clonal’ if the 95% confidence interval for the CCF estimate included 1, and ‘subclonal’ otherwise. Darker boxes indicate that the proportion of clonal mutations for the specified gene in the specified IntClust is higher. All coding mutations are shown.

Supplementary Fig. 14

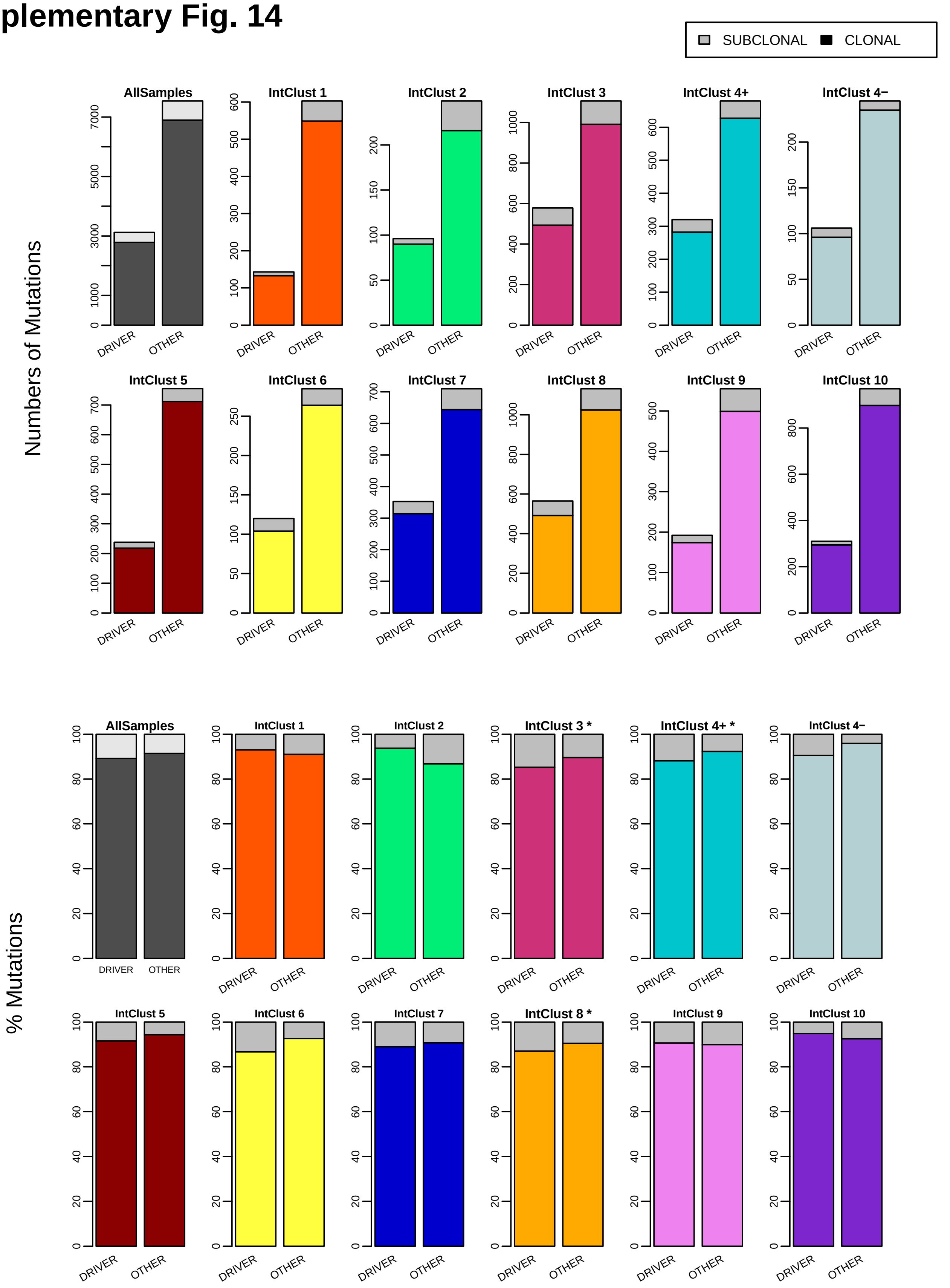

Supplementary Figure 14 – Clonal states of driver mutations.
Bars show the fractions of clonal (coloured) or subclonal (gray) mutations for driver mutations (recurrent or inactivating; as described in the main text) and non-driver coding mutations across the IntClusts. These are plotted as absolute numbers (a) and as percentages (b) of all coding mutations in the indicated category. The ***** in (b) indicate those IntClusts in which there is a significant difference in the proportions of clonal and subclonal mutations between driver and non-driver mutations.

Supplementary Fig. 15

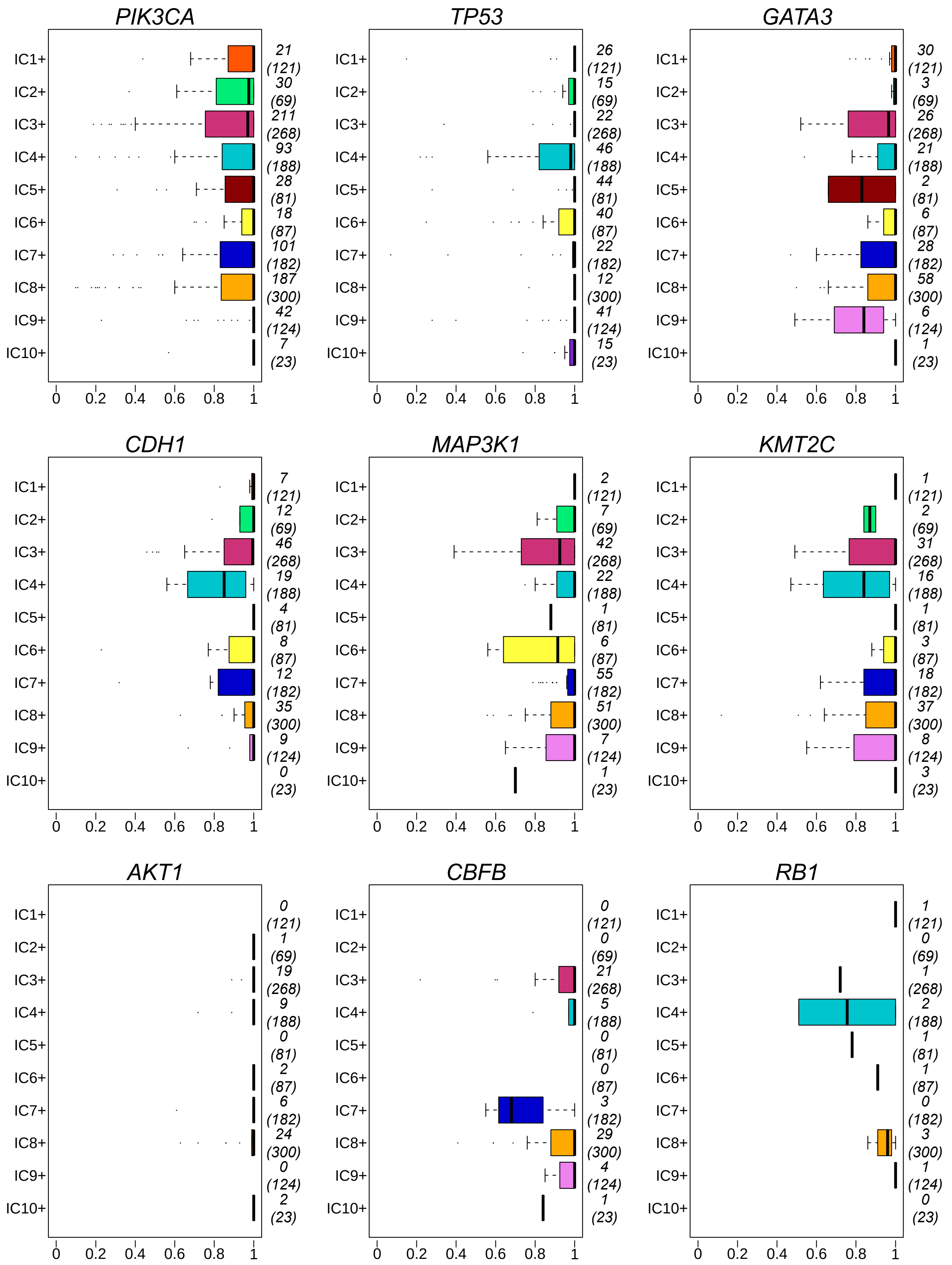

Supplementary Figure 15 – Cancer cell fractions of Mut-driver gene mutations in ER+ tumours. The cancer cell fractions (CCFs) of functional mutations in genes mutated at significantly different frequencies between IntClusts. The CCF distributions are shown for the ER+ tumours within the IntClusts; numbers indicate the number of mutations in a subgroup, whereas the numbers in brackets indicate the number of patients in the IntClust.

Supplementary Fig. 16

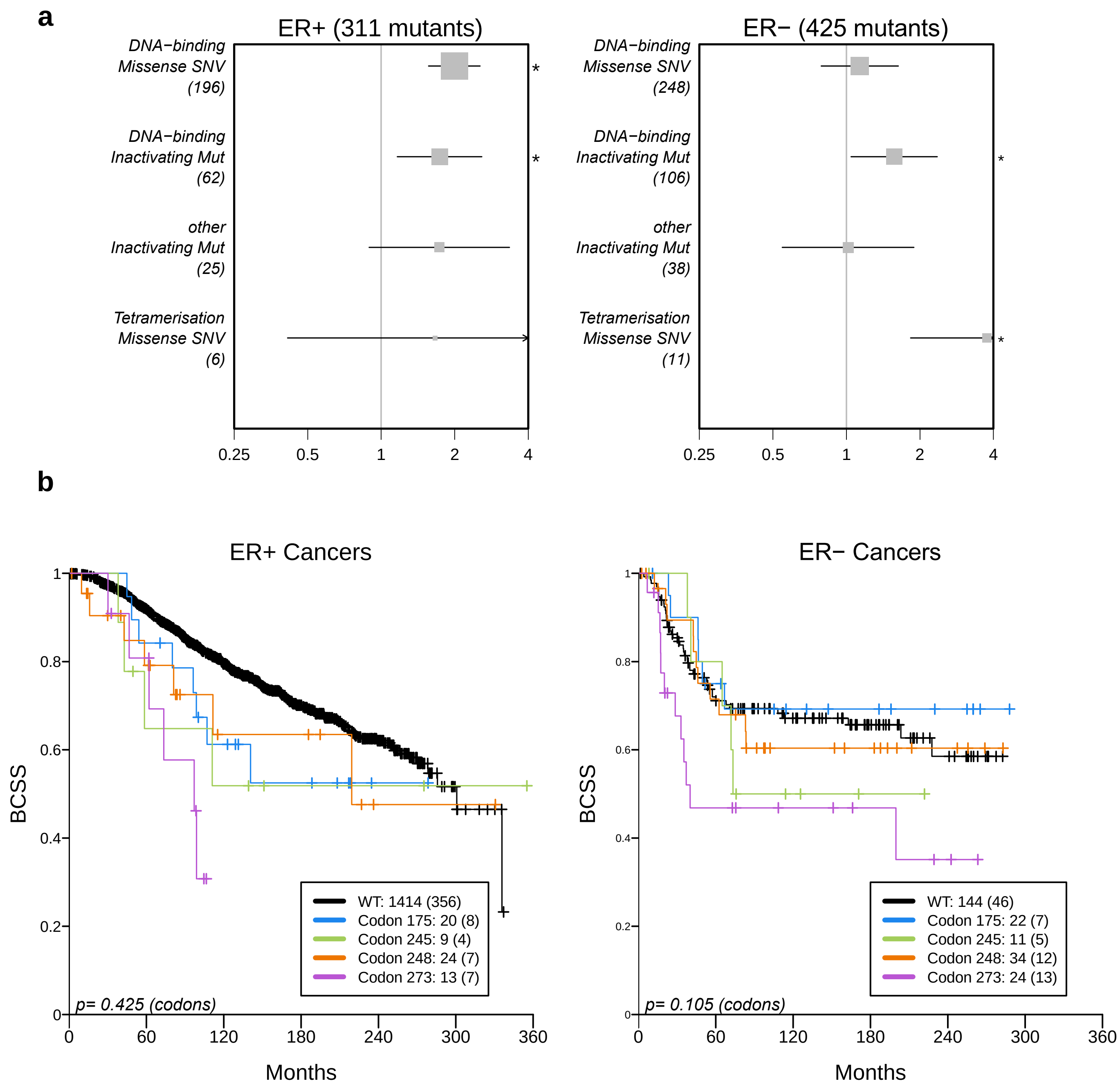

Supplementary Figure 16 – Associations between *TP53* mutations and breast cancer-specific survival.

a) Mutations occurring in *TP53* were categorised according the domains indicated, and Cox proportional hazards models were used to examine the effect on breast cancer-specific survival (BCSS) of mutations in different functional domains. The boxes indicate hazard ratios, and lines and box sizes represent confidence intervals. Numbers in brackets refer to the number of tumours harbouring a mutation in that class. All hazard ratios are shown relative to TP53 wild-type samples.

b) Within the DNA-binding domain, codons 175, 245, 248 and 273 are the most frequently mutated. Kaplan-Meier curves were constructed to explore the effects of mutations in these residues. The p-value was computed to check if there was a significant survival difference between the four codons, and does not take the wild-type curve into account.

Supplementary Fig. 17

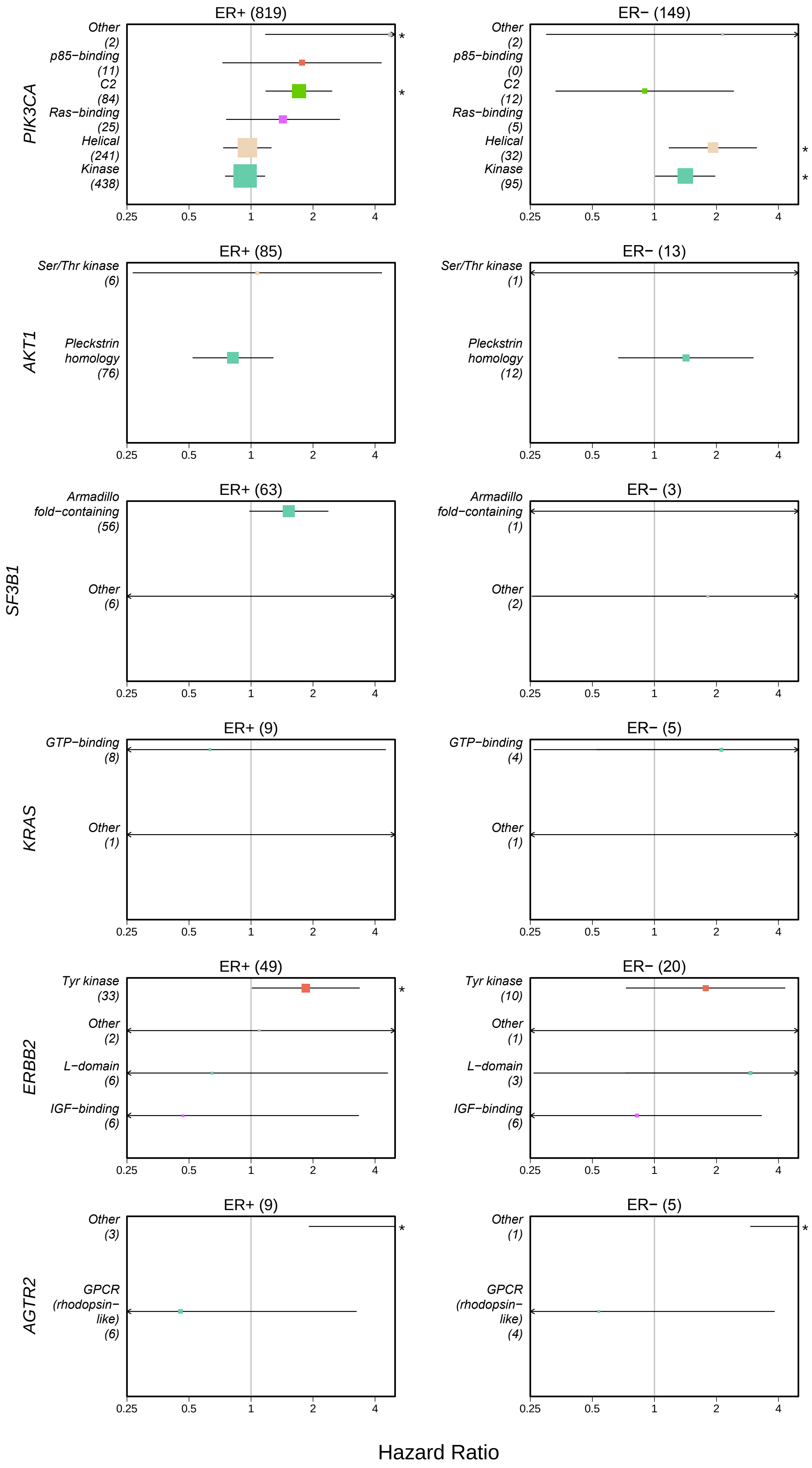

Supplementary Figure 17 – Associations between mutations in functional domains and breast cancer-specific survival for six oncogenes. Cox models were constructed as for *TP53* in Supp. Fig. 17 for the six oncogenes identified in the Mut-driver list. All hazard ratios are shown relative to wild-type samples for the particular gene.

Supplementary Fig. 18

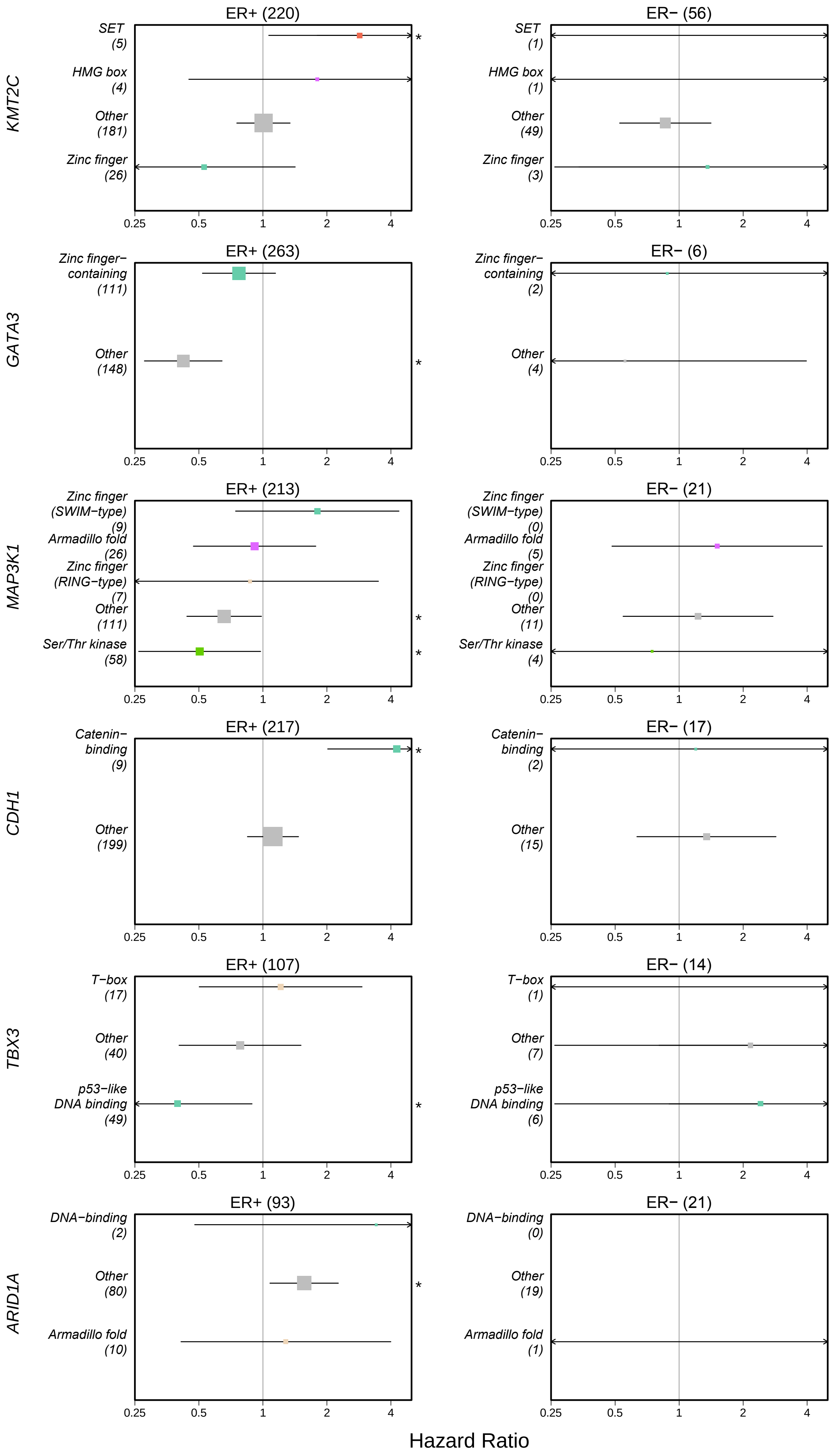

Supplementary Figure 18 – Associations between mutations in functional domains and breast cancer-specific survival for tumour suppressor genes. Cox models were constructed as for *TP53* in Supp. Fig. 17 for tumour suppressor genes mutated in at least 5% of ER+ or ER- samples (excluding *TP53*). All hazard ratios are shown relative to wild-type samples for the particular gene.

Supplementary Fig. 19

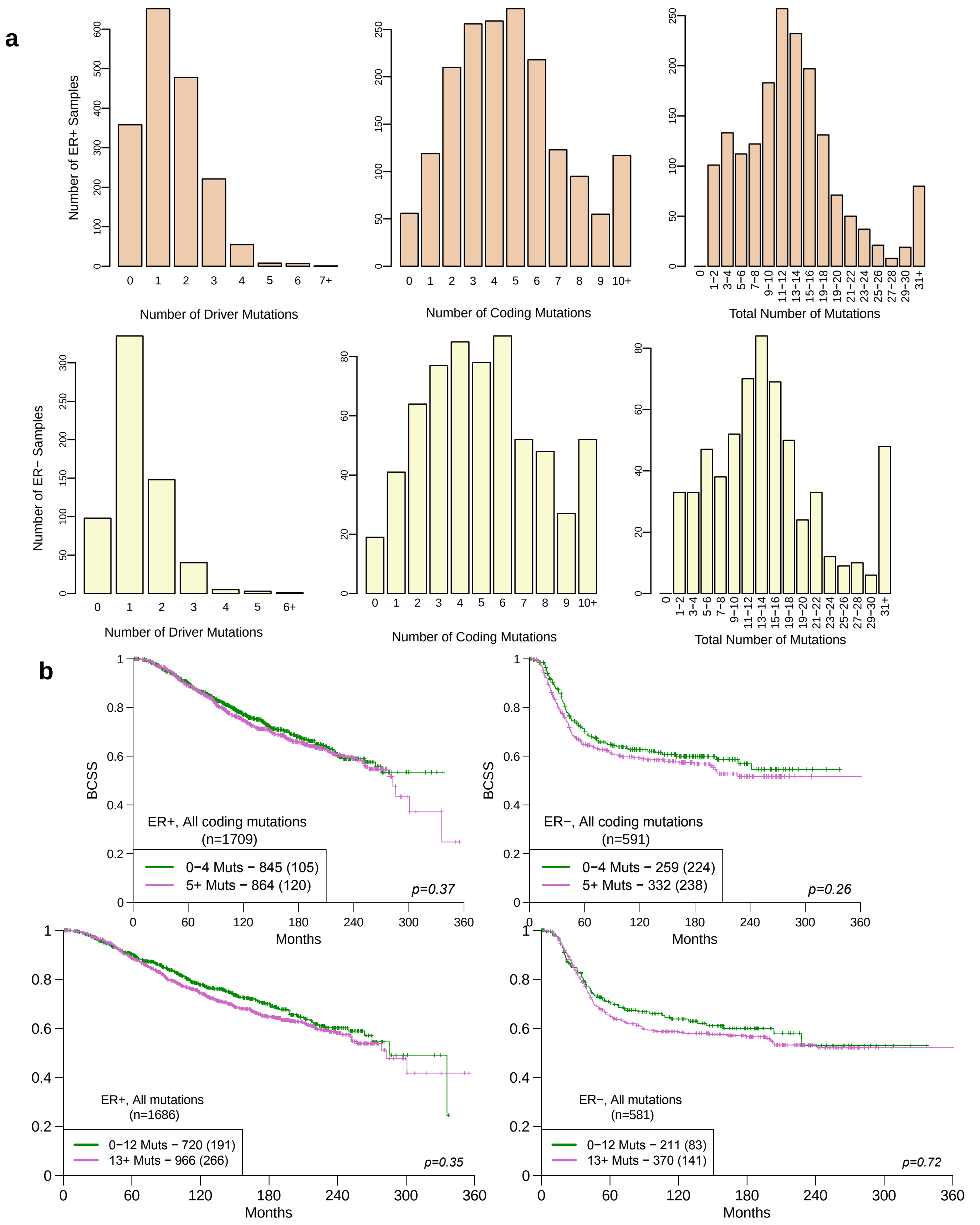

Supplementary Figure 19 – Prognostic effects of the number of mutations in Mut-driver genes.

a) Bars show the distribution of numbers of functional mutations in Mut-driver genes, numbers of all coding mutations, and numbers of all mutations in ER+ (top) and ER- (bottom) samples.

b) Univariable Cox models were constructed to determine the effect of mutation counts on breast cancer-specific survival (BCSS). Kaplan-Meier curves showing BCSS in ER+ (left panels) and ER- (right panels) disease according to number of coding mutations (top panels) and all mutations (bottom panels)

Supplementary Fig. 20

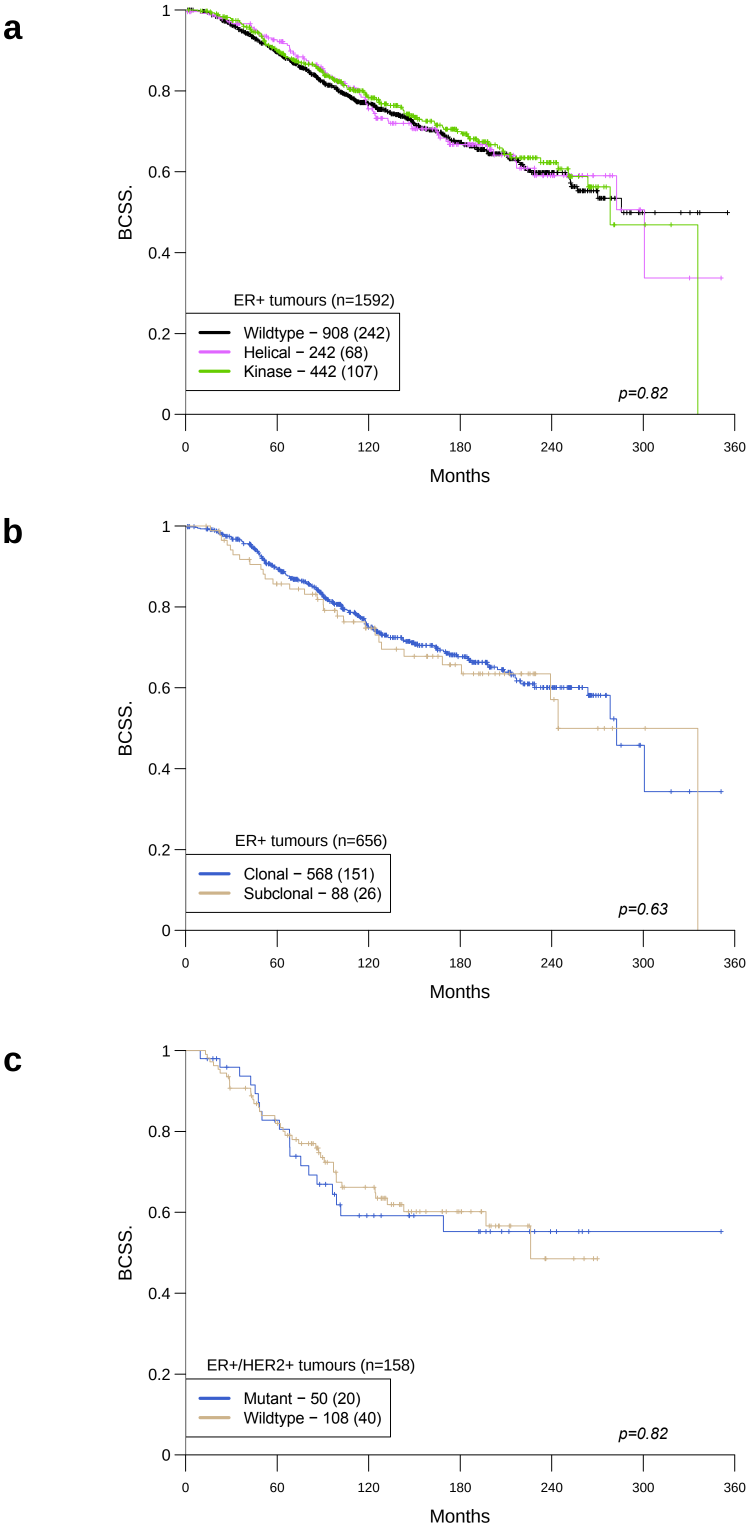

Supplementary Figure 20 – Prognostic value of *PIK3CA* mutations in ER+ tumours
Kaplan-Meier curves were constructed to examine the association of *PIK3CA* mutations with breast cancer-specific survival (BCSS) in ER+ tumours after stratifying by mutation position, CCF and HER2 status. Numbers in the bottom-left corner indicate numbers of patients falling within the specified category, and, in brackets, number of BCSS events within the group.

a) Associations between mutations in either the helical or kinase domains of *PIK3CA* and BCSS.
b) Associations between *PIK3CA* mutation CCF (***Methods***) and BCSS.
c) Associations between *PIK3CA* mutations and BCSS after stratifying by HER2 status.

Supplementary Fig. 21

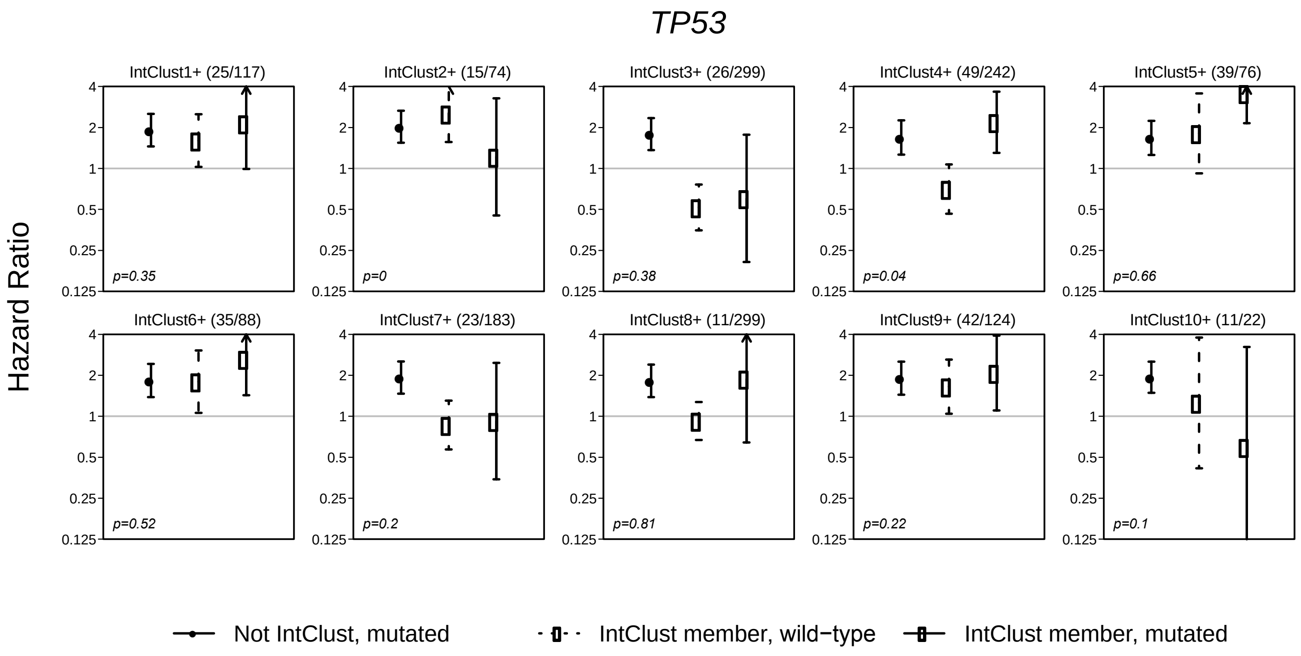

Supplementary Figure 21 – Prognostic impact of Mut-driver genes mutations in ER+ tumours across the IntClusts.
Univariable Cox models were constructed with interaction terms for IntClust membership (ER+ tumours within an IntClust) and mutation status as in ***Figure 6b***. The hazard ratios are shown for mutations (black circles), IntClust membership without mutation (coloured circles, dashed lines) and mutations within the IntClust (coloured circles, solid lines).

Class	TotalNumber	ER+	ER-	CopyNumber	UnivariableSurvival	MultivariableSurvival
Primary Tumours	2433	1780	630	2087	2315	2122
IntClust1	145	127	16	141	135	129
IntClust2	79	74	5	76	78	73
IntClust3	319	306	13	292	312	270
IntClust4	359	247	111	337	353	311
IntClust5	215	85	130	202	193	179
IntClust6	91	91	0	88	88	82
IntClust7	193	188	3	188	188	176
IntClust8	318	313	4	309	303	275
IntClust9	161	133	28	151	149	132
IntClust10	246	27	219	238	228	220

Supplementary Table 1 – Details of samples used in this study. The numbers of samples in each of the specified categories is shown. TotalNumber = total number of samples; ER+ = number of ER+ samples; ER- = number of ER- samples; CopyNumber = number of samples with copy number data available; UnivariableSurvival = number of samples with univariable survival data available (time until and status at censoring); MultivariableSurvival = as for UnivariableSurvival, but also with data for tumour size, grade, patient age, and lymph node involvement. Some tumours had missing data in certain categories. For example, 2433 primary tumours were used in this study, but IntClust classification was available for only 2126 (due to lack of copy number/gene expression data or failed classification).

Gene	Driver Type	Number of Recurrent Mutations (all samples)	Oncogene Score (all samples)	Hotspot Codons
PIK3CA	Oncogene	1044	93%	81, 88, 93, 104, 106, 107, 108, 110, 111, 118, 345, 365, 420, 448, 449, 453, 471, 542, 545, 546, 726, 727, 970, 1007, 1017, 1021, 1043, 1044, 1047, 1048, 1049
AKT1	Oncogene	87	76%	17, 52, 80
KRAS	Oncogene	11	79%	12
ERBB2	Oncogene	41	41%	310, 523, 754, 755, 769, 777, 842
SF3B1	Oncogene	42	45%	666, 700
AGTR2	Oncogene	6	43%	271
TP53	Tumour suppressor gene	468	52%	110, 111, 127, 131, 132, 134, 135, 141, 151, 157, 159, 163, 173, 175, 176, 177, 178, 179, 193, 194, 195, 197, 205, 216, 220, 234, 236, 237, 238, 239, 241, 242, 244, 245, 246, 248, 249, 250, 255, 257, 262, 266, 270, 272, 273, 278, 280, 281, 282, 285, 286, 337, 342, 348

Supplementary Table 2 – Recurrent mutations in the six identified oncogenes and *TP53*. Using the ratiometric method described in the text, we identified six oncogenes based on the proportion of recurrent mutations they harboured (oncogene score). *TP53* also had a high proportion of recurrent mutations, although the presence of inactivating mutations suggests that it is a tumour suppressor gene. Recurrent mutations were defined as missense SNVs and in-frame indels that affected the same codon. Mutations occurring at these codons were used in downstream analyses.

Supplementary Table 3 – Percentages (%) of tumours within the IntClusts harbouring functional mutations in Mut-driver genes. The number of tumours (N) is indicated for each IntClust. Mut-driver genes and functional mutations were defined as described in the main text.

IntClust	1	2	3	4+	4-	5	6	7	8	9	10
**N=**	145	79	319	247	111	215	91	193	318	161	246
AKT1	0	1.3	6.9	4	4.5	0.5	2.2	3.6	7.5	1.2	1.6
PIK3CA	18.6	36.7	62.7	42.1	34.2	31.6	19.8	51.3	55.3	31.7	11
PIK3R1	0	0	0.9	0.4	0.9	0	2.2	0	0.9	0.6	1.6
PTEN	0	0	4.4	1.6	4.5	0.5	5.5	1.6	2.8	3.1	2
CDKN1B	0	0	0.6	1.2	0	0	0	0	1.9	1.2	0.4
CDKN2A	0	1.3	0.3	0	0.9	0	0	0	0	1.2	0
RB1	0.7	0	0.3	0.8	0.9	0.5	1.1	0	0.9	1.2	5.3
ARID1A	3.4	1.3	1.9	2.4	2.7	2.3	7.7	2.1	3.5	2.5	0.8
CTCF	0	1.3	0.3	0.4	0	0.5	1.1	2.6	1.6	0	0
KDM6A	0	0	0.3	1.2	2.7	0	0	0.5	0.3	0	0.8
KMT2C	0.7	2.5	10.7	5.3	3.6	1.9	3.3	8.8	12.3	5.6	3.3
NCOR1	2.1	2.5	3.4	0.8	0.9	2.8	0	2.1	1.9	0	0.4
PBRM1	0.7	0	0	0	0	0.5	0	0	0	0	1.6
BRCA1	1.4	0	0.3	0	1.8	0.5	0	0.5	0	0.6	2.4
BRCA2	0.7	1.3	0.6	1.2	0.9	0.5	0	1.6	0	0	0.8
CHEK2	0.7	0	0.3	0	0	0	0	0	0.3	0.6	0
TP53	29	24.1	10	21.1	50.5	64.2	40.7	14	4.4	44.7	84.6
AGTR2	0.7	0	0.6	0	0	0	0	0.5	0	0	0.4
GPS2	0	0	1.6	1.2	0	0	2.2	3.6	0.9	0.6	0
KRAS	0	0	0.3	0.8	0.9	0	0	0.5	0.9	0.6	0.4
MAP2K4	2.8	1.3	2.2	3.6	0.9	0.9	2.2	4.7	1.3	1.9	0.4
MAP3K1	1.4	6.3	11.3	8.1	0.9	0.9	4.4	21.2	12.9	4.3	0.8
NF1	4.1	2.5	2.8	3.2	0.9	0.9	1.1	2.1	0.6	1.2	2.4
SF3B1	2.1	2.5	2.8	2.4	0	0	3.3	3.6	1.3	2.5	0

ERBB2	0.7	1.3	2.5	2.4	2.7	0.9	0	2.6	2.5	1.2	0.4
SMAD4	0.7	1.3	1.3	0.8	0	0.5	0	0.5	0.3	0	0
CDH1	4.8	17.7	18.2	8.5	2.7	2.8	9.9	5.7	11.9	6.8	0.4
CTNNA1	0	0	0.3	0	0.9	0	0	0	0.3	0.6	0
MLLT4	0.7	1.3	0.9	0.4	2.7	1.4	2.2	0.5	0.3	0	0.8
CBFB	0	1.3	7.8	1.6	0	0	0	1	9.7	2.5	0.4
FOXO3	2.1	1.3	0.3	1.6	0	0	0	2.6	0.3	0	0.4
FOXP1	0	0	0.6	0.4	0	0.5	0	0.5	0.6	0	0
GATA3	20	5.1	8.5	8.9	0.9	1.4	6.6	15	19.5	3.1	0.8
MEN1	0	1.3	0	0.8	0	0	1.1	0.5	0	0	0
RUNX1	0.7	0	4.7	0.4	0	4.2	3.3	1	3.8	0.6	1.2
TBX3	1.4	3.8	6.9	2.8	0	2.3	0	5.7	4.7	1.9	0
ZFP36L1	0	0	1.6	1.2	0	0.5	1.1	0.5	0.6	1.9	1.6
BAP1	0	0	0	0	0	0.5	0	0	0	0	1.2
USP9X	0.7	3.8	0.3	0.4	0.9	0.5	0	0.5	0.6	0.6	0.8
TBL1XR1	0	1.3	0	0.4	0	0	0	0.5	0.3	0	0

Supplementary Table 4 – Percentages (%) of tumours within the the ER+ populations of the IntClusts harbouring functional mutations in Mut-driver genes. As for ***Supplementary Table 3***, but considering only ER+ cancers.

IntClust	IntClust1+	IntClust2+	IntClust3+	IntClust4+	IntClust5+	IntClust6+	IntClust7+	IntClust8+	IntClust9+	IntClust10+
**N=**	127	74	306	247	85	91	188	313	133	27
AKT1	0	1.4	6.9	4	0	2.2	3.7	7.7	0	7.4
PIK3CA	18.1	37.8	64.1	42.1	34.1	19.8	51.1	55.6	33.1	29.6
PIK3R1	0	0	0.7	0.4	0	2.2	0	1	0.8	3.7
PTEN	0	0	4.6	1.6	1.2	5.5	1.6	2.9	1.5	7.4
CDKN1B	0	0	0.7	1.2	0	0	0	1.9	1.5	0
CDKN2A	0	1.4	0.3	0	0	0	0	0	1.5	0
RB1	0.8	0	0.3	0.8	1.2	1.1	0	1	0.8	3.7
ARID1A	3.9	1.4	2	2.4	4.7	7.7	2.1	3.2	3	7.4
CTCF	0	1.4	0.3	0.4	0	1.1	2.7	1.6	0	0
KDM6A	0	0	0.3	1.2	0	0	0.5	0.3	0	3.7
KMT2C	0.8	2.7	10.8	5.3	1.2	3.3	9	12.1	6	11.1
NCOR1	2.4	2.7	3.3	0.8	4.7	0	2.1	1.9	0	3.7
PBRM1	0.8	0	0	0	0	0	0	0	0	0
BRCA1	1.6	0	0.3	0	1.2	0	0.5	0	0	0
BRCA2	0.8	0	0.7	1.2	0	0	1.6	0	0	0
CHEK2	0.8	0	0.3	0	0	0	0	0.3	0.8	0
TP53	22	20.3	8.5	21.1	52.9	40.7	12.8	3.8	34.6	59.3
AGTR2	0.8	0	0.7	0	0	0	0.5	0	0	0
GPS2	0	0	1.6	1.2	0	2.2	3.7	1	0	0
KRAS	0	0	0.3	0.8	0	0	0.5	1	0.8	0
MAP2K4	3.1	1.4	2.3	3.6	1.2	2.2	4.8	1.3	2.3	0

MAP3K1	1.6	6.8	11.8	8.1	1.2	4.4	21.8	13.1	5.3	3.7
NF1	3.9	1.4	2.3	3.2	1.2	1.1	1.1	0.6	1.5	0
SF3B1	2.4	2.7	2.9	2.4	0	3.3	3.7	1.3	3	0
ERBB2	0	0	2	2.4	0	0	2.1	2.6	0.8	3.7
SMAD4	0.8	1.4	1.3	0.8	1.2	0	0.5	0.3	0	0
CDH1	5.5	17.6	18	8.5	4.7	9.9	5.9	11.8	7.5	3.7
CTNNA1	0	0	0.3	0	0	0	0	0.3	0.8	0
MLLT4	0.8	1.4	1	0.4	0	2.2	0.5	0.3	0	0
CBFB	0	1.4	8.2	1.6	0	0	1.1	9.6	3	3.7
FOXO3	2.4	1.4	0.3	1.6	0	0	2.7	0.3	0	0
FOXP1	0	0	0.7	0.4	1.2	0	0.5	0.6	0	0
GATA3	22.8	5.4	8.8	8.9	2.4	6.6	15.4	19.8	3.8	3.7
MEN1	0	1.4	0	0.8	0	1.1	0.5	0	0	0
RUNX1	0.8	0	4.9	0.4	3.5	3.3	1.1	3.8	0.8	0
TBX3	0.8	4.1	7.2	2.8	2.4	0	5.3	4.8	2.3	0
ZFP36L1	0	0	1.6	1.2	0	1.1	0.5	0.6	2.3	0
BAP1	0	0	0	0	0	0	0	0	0	0
USP9X	0.8	4.1	0.3	0.4	1.2	0	0.5	0.6	0	3.7
TBL1XR1	0	1.4	0	0.4	0	0	0.5	0.3	0	0

Gene	Hazard Ratio ER+	P-value ER+	Hazard Ratio ER-	P-value ER-
AGTR2	1 (0.14-7.3)	0.986	1.3e-06 (0-Inf)	0.993
AKT1	0.81 (0.51-1.3)	0.389	1.3 (0.58-3)	0.504
ARID1A	1.6 (1-2.5)	0.051	0.87 (0.12-6.2)	0.891
BAP1	2.8e-06 (0-Inf)	0.991	0.42 (0.059-3)	0.393
BRCA1	1.7 (0.23-12)	0.616	0.53 (0.13-2.1)	0.368
BRCA2	0.82 (0.2-3.3)	0.775	2.4 (0.85-6.5)	0.098
CBFB	0.69 (0.41-1.2)	0.157	NA (NA-NA)	NA
CDH1	1.2 (0.88-1.6)	0.258	1.3 (0.51-3.2)	0.59
CDKN1B	1.1 (0.35-3.4)	0.887	1.4e-06 (0-Inf)	0.994
CDKN2A	0.65 (0.091-4.6)	0.662	3.1e-07 (0-Inf)	0.993
CHEK2	0.74 (0.1-5.3)	0.765	NA (NA-NA)	NA
CTCF	0.54 (0.13-2.2)	0.382	NA (NA-NA)	NA
CTNNA1	0.5 (0.071-3.6)	0.493	1.3e-06 (0-Inf)	0.992
ERBB2	1.8 (0.84-3.8)	0.129	0.71 (0.17-2.9)	0.629
FOXO3	0.84 (0.27-2.6)	0.757	1.3e-06 (0-Inf)	0.993
FOXP1	2.5 (0.81-7.9)	0.11	NA (NA-NA)	NA
GATA3	0.58 (0.4-0.82)	0.002	2.6 (0.36-19)	0.339
GPS2	0.54 (0.17-1.7)	0.289	5.9 (0.81-43)	0.079
KDM6A	1.7 (0.42-6.8)	0.462	0.6 (0.084-4.3)	0.616
KMT2C	1 (0.72-1.5)	0.9	1.1 (0.44-2.6)	0.89
KRAS	0.66 (0.093-4.7)	0.682	2.2 (0.3-16)	0.447
MAP2K4	1.4 (0.81-2.3)	0.239	2.5 (0.62-10)	0.196
MAP3K1	0.56 (0.38-0.82)	0.003	1.1 (0.27-4.5)	0.894
MEN1	2.4 (0.76-7.5)	0.138	NA (NA-NA)	NA
MLLT4	2.1 (0.94-4.7)	0.072	0.9 (0.28-2.8)	0.854
NCOR1	0.66 (0.27-1.6)	0.364	1.2 (0.17-8.6)	0.856
NF1	1.5 (0.81-2.8)	0.194	2.7 (1.3-5.5)	0.006
PBRM1	NA (NA-NA)	NA	2 (0.62-6.3)	0.247
PIK3CA	1.1 (0.9-1.3)	0.418	1.4 (1-1.9)	0.033
PIK3R1	1.8 (0.74-4.3)	0.198	2.8e-07 (0-Inf)	0.99
PTEN	0.83 (0.43-1.6)	0.581	1.2 (0.52-2.6)	0.71
RB1	1.2 (0.55-2.8)	0.593	1.5 (0.7-3.2)	0.289
RUNX1	0.64 (0.26-1.5)	0.315	2.2 (0.92-5.5)	0.076
SF3B1	1.4 (0.8-2.4)	0.235	NA (NA-NA)	NA
SMAD4	3.4 (1.4-8.3)	0.007	NA (NA-NA)	NA
TBL1XR1	1.9 (0.6-5.8)	0.284	NA (NA-NA)	NA
TBX3	0.8 (0.45-1.4)	0.454	1.6 (0.4-6.6)	0.502
TP53	1.6 (1.3-2)	<0.001	1.1 (0.8-1.5)	0.551
USP9X	3 (1.2-7.2)	0.016	2.7 (0.66-11)	0.167
ZFP36L1	0.6 (0.15-2.4)	0.476	1.3 (0.4-4)	0.702

Supplementary Table 5 – Survival analysis of Mut-driver mutations. Multivariable Cox models were constructed as described in the text, and hazard ratios associated with functional mutations in Mut-driver genes are depicted by 'Hazard Ratio estimate (95% confidence interval)' in the table. The analysis was performed separately for ER+ and ER- tumours. Breast cancer-specific death was used as an endpoint. NAs are indicated where there is an insufficient number of mutations to inform the model.

IntClust	MATH_lower_quartile	MATH_median	MATH_upper_quartile
1	0.26	0.37	0.53
2	0.16	0.25	0.37
3	0.17	0.29	0.45
4+	0.16	0.24	0.40
4-	0.19	0.27	0.54
5	0.23	0.39	0.53
6	0.21	0.35	0.48
7	0.20	0.27	0.40
8	0.18	0.28	0.39
9	0.25	0.41	0.58
10	0.31	0.47	0.61

Supplementary Table 6– MATH score distributions. Mutant allele tumour heterogeneity (MATH) scores were calculated as described in the text for each tumour. The distributions of MATH scores (lower quartile, median, upper quartile) are shown for each IntClust. Only tumours with 5 or more mutations were used for this analysis.

Exons covered	Forward primer (5')	Reverse primer (3')	PCR fragment length (Bp)
2 and 3	GGAGTGCTTGGGTTGTGGT	CGGCAAGGGGGACTGTA	586
4	GACTTCCTGAAAACAACG	CACACATTAAGTGGGTAAAC	593
5 and 6	TTTCTT TGCTGC CGTCTTC	TTGCAC ATCTCATGGGGTTA	588
7	CTTGCCACAGGTCTCCCCAA	AGGGGTCAGCGGCAAGCAGA	237
8 and 9	TTTGGG ACCTCT TAACCTGT	CAGGCA AAGTCATAGAACCAT	733
10	CATGTTGCTTTTGTACCG TC	GGCAAGAATGTGGTTATAGGA	396
11	AAG GGAAGATTACGAGACT	TAAGCTGGTATGTCC TACTC	500

Supplementary Table 7 – Primers for validating *TP53* mutations by PCR and Sanger sequencing. Sanger sequencing was used to call *TP53* mutations for METABRIC tumours in the previous study[1]. We used this dataset to validate the mutation calls derived from our pipeline. Both the forward and reverse primers were linked to the universa M13 sequences in the 5' position. M13 forward primer: TGTAAAACGACGGCCAGT. M13 reverse primer sequence: CAGGAAACAGCTATGACC

Supplementary Reference

1. Silwal-Pandit, L. et al. TP53 Mutation Spectrum in Breast Cancer Is Subtype Specific and Has Distinct Prognostic Relevance 20(13); 1–12 (2014) *Clinical Cancer Res*
